# Cervical whole-slide images dataset for multiclass classification

**DOI:** 10.1093/gigascience/giaf144

**Published:** 2025-11-29

**Authors:** Mahnaz Mohammadi, Christina Fell, David Morrison, Sarah Bell, Gareth Bryson, Sheeba Syed, Prakash Konanahalli, David Harris-Birtill, Ognjen Arandjelovic, Clare Orange, Prishma Shahi, In Hwa Um, James D Blackwood, David J Harrison

**Affiliations:** School of Medicine, University of St Andrews, North Haugh, St Andrews, KY16 9TF, United Kingdom; School of Medicine, University of St Andrews, North Haugh, St Andrews, KY16 9TF, United Kingdom; School of Medicine, University of St Andrews, North Haugh, St Andrews, KY16 9TF, United Kingdom; Department of Pathology, Queen Elizabeth University Hospital, Glasgow, G51 4TF, United Kingdom; Department of Pathology, Queen Elizabeth University Hospital, Glasgow, G51 4TF, United Kingdom; Department of Pathology, Queen Elizabeth University Hospital, Glasgow, G51 4TF, United Kingdom; Department of Pathology, Queen Elizabeth University Hospital, Glasgow, G51 4TF, United Kingdom; School of Computer Science, University of St Andrews, North Haugh, St Andrews, KY16 9SX, United Kingdom; School of Computer Science, University of St Andrews, North Haugh, St Andrews, KY16 9SX, United Kingdom; School of Medicine, University of St Andrews, North Haugh, St Andrews, KY16 9TF, United Kingdom; Department of Pathology, Queen Elizabeth University Hospital, Glasgow, G51 4TF, United Kingdom; School of Medicine, University of St Andrews, North Haugh, St Andrews, KY16 9TF, United Kingdom; School of Medicine, University of St Andrews, North Haugh, St Andrews, KY16 9TF, United Kingdom; Pathology, Division of Laboratory Medicine, Royal Infirmary of Edinburgh, Edinburgh, EH16 4SA, United Kingdom; School of Medicine, University of St Andrews, North Haugh, St Andrews, KY16 9TF, United Kingdom; School of Medicine, University of St Andrews, North Haugh, St Andrews, KY16 9TF, United Kingdom; Pathology, Division of Laboratory Medicine, Royal Infirmary of Edinburgh, Edinburgh, EH16 4SA, United Kingdom

**Keywords:** whole-slide imaging, histopathology, cervix, cervical cancer, digital image database, machine learning, deep learning, health care dataset

## Abstract

**Background:**

The clinical pathway for the prevention and treatment of cervical cancer depends on cytology and then the assessment of biopsy specimens, fragments of tissue removed for histological examination. This can be a significant workload and is an obvious exemplar to explore triage based on machine learning analysis of slides. Limited access to large annotated datasets of human diseased tissue is a major obstacle to developing standards and algorithms that can assist diagnosis.

**Results:**

We present a dataset comprising 2,539 whole-slide images of cervical biopsy specimens, each annotated by several pathologists and consensus on diagnosis and individual features agreed. Each whole-slide image represents 1 slide per patient in iSyntax format, with manual annotations by pathologists in Jason format. Each whole-slide image is assigned a category label, which is the final diagnosis of the image, and a subcategory label, which declares in which subcategory the image is found.

**Conclusion:**

This dataset has been used to build a model that accurately predicts diagnosis, allowing the possibility of automatically triaging biopsy specimens, so that the most significant pathologies can be identified rapidly and those patients selected for immediate treatment. The level of annotation, at the subslide level, and the number of cases are unique in public databases and should allow investigators to explore multiple aspects of computer vision relevant to human tissue diagnosis, with no limitation placed on access to the whole-slide images.

## Data Description

This large collection of cervical whole-slide image (WSI) data serves as a vital resource to improve cervical cancer detection, diagnosis, and research, ultimately contributing to the overall goal of reducing the burden of this preventable and treatable disease on a global scale. Cervical WSI data provide detailed, high-resolution views of cervical tissue samples, enabling health care providers to detect abnormalities and precancerous lesions with greater precision. This improved accuracy can lead to earlier intervention and improved patient outcomes. Researchers and data scientists utilize this comprehensive dataset to develop and fine-tune machine learning (ML) algorithms and artificial intelligence (AI) tools. These tools can automate and streamline the screening process, making it more accessible and cost-effective, particularly in regions with limited health care resources.

The dataset consists of a total of 2,539 hematoxylin and eosin (H&E) cervical WSIs, with 1 slide per patient, in iSyntax format requiring 731 GB of storage. This dataset was originally created for the Gynaecological Cancer AI project, which is part of the Industrial Centre for Artificial Intelligence Research in Digital Diagnostics (iCAIRD) [1] and was used to develop and train AI for the diagnosis of cervical biopsy specimens as either benign dysplastic (precancerous) or neoplastic (cancer). For abnormal biopsy specimens, classification was as follows: (i) invasive squamous carcinoma or adenocarcinoma and (ii) low-grade (including Human papilloma virus (HPV) infection and cervical intraepithelial neoplasia (CIN) 1) or high-grade (including CIN 2 and CIN 3) intraepithelial neoplasia.

## Context

As the demand for AI tools for diagnosis continues to grow, so does the need for high-quality datasets. Datasets are a critical component of AI development as they provide the training data that enable the machine learning (ML) models to learn patterns and relationships, as well as make predictions. Datasets can be used for training, evaluating, and testing different ML models. They also can serve as benchmarks for comparing different algorithms and models. Datasets contribute to the advancement of ML research by providing a foundation for exploring new algorithms, techniques, and models. Hence, quality, diversity, representativeness, size, balance, and potential biases of the dataset are factors that can significantly impact the performance and generalization of ML applications.

Digital pathology offers several benefits and addresses various challenges in traditional pathology practice, making it a valuable and increasingly essential component of modern health care. AI has become increasingly important in the field of digital pathology due to its potential to revolutionize the way medical professionals analyze and interpret pathology slides. AI can help identify patterns that may be difficult to spot with the human eye, leading to faster and more accurate diagnoses. It can aid in the early detection of diseases like cancer by analyzing subtle changes in images over time, which can lead to earlier interventions and improved patient outcomes.

Histopathology is the microscopic examination of tissue samples to diagnose diseases and understand their underlying causes. Histopathology WSIs are indispensable resources in the field of medical image analysis and ML applications. Staining techniques are essential in histopathology to enhance the visualization of cellular structures and specific components within tissues. Different staining techniques are used to highlight various tissue elements and help pathologists differentiate between normal and abnormal structures. The digital representations of tissue samples stained with H&E provide a comprehensive view of cellular and tissue structures at a microscopic level. The color contrast given to different cell types and tissue components aids pathologists in diagnosing and characterizing diseases and provides a rich source of visual and contextual information, making them an ideal input for various ML tasks.

ML algorithms trained on a large dataset of annotated H&E WSIs can be useful in diagnostic assistance; predicting disease progression, drug discovery, and development; educational tools; and many more. Trained ML algorithms on WSIs can assist pathologists in diagnosing diseases and identifying patterns and anomalies in tissue samples, which can lead to more accurate and efficient diagnoses. Detecting and segmenting tumor regions within H&E WSIs is another example that aids in quantifying tumor size, density, and distribution, which are essential factors in disease prognosis and treatment planning. Meaningful features extracted from H&E WSIs, such as texture, shape, and color information, can be used to characterize tissue structures, helping researchers and clinicians understand tissue composition and potentially uncover new insights.

A comprehensive survey of cervical histopathology image analysis using machine vision approaches is presented [2]. This article reviews all the related works of cervical histopathology image analysis using machine vision techniques from 1988 to 2020. In this survey, more than 60 related works are summarized from 1988 to 2019. Li et al. [3] propose a graph-based unsupervised learning approach to describe the topological information of different tissues in the histopathological images, and 2 stages of unsupervised leaning processes are applied to group the tissues into relevant types. A weakly supervised survival convolutional neural network approach equipped with a visual attention mechanism for predicting overall survival is presented in [4]. The inclusion of visual attention provides insights into regions of the tumor microenvironment with the pathological interpretation, which may improve understanding of the disease pathomechanism. This analysis is performed on 2 independent, multicenter patient datasets of lung carcinoma (which are publicly available data) and bladder urothelial carcinoma. The presented results highlight the significance of computational pathology algorithms for predicting prognosis using H&E-stained images alone and underpin the use of computational methods to improve the efficiency of clinical trial studies.

In the gynecological cancer AI project at iCAIRD, AI algorithms were trained and evaluated on cervical biopsy specimens for automated reporting of digital diagnostics, with the aim to increase the overall efficiency of pathological diagnoses and to have the performance tuned to a high sensitivity for malignant cases. The algorithms were trained and validated on 1,738 cervical WSIs. On the independent test set of 811 WSIs, the trained algorithm achieved 93.4% malignant sensitivity for classifying slides [5].

## Methods

### Data collection

The cervical tissue blocks were originally collected from the achieves of the Glasgow Royal Infirmary (NG), Southern General Hospital (SG), Royal Alexandria Hospital (RAH), and Queen Elizabeth University Hospital (QEUH) (all in Glasgow, Scotland), each with independent tissue handling, including fixation and tissue processing. The number of tissue blocks obtained from each of the above sites was as follows: 829 from QEUH, 729 from NG, 647 from SG, and 334 from RAH. Since some of these slides were faded, new tissue sections were cut from the tissue blocks at 1 of 2 different thicknesses (3 or 4 microns) and then stained with 1 of 4 different H&E protocols (routine H&E, muscle biopsy protocol, neuro protocol, and pediatric tissues protocol). Together, these combinations gave 8 different labs maximizing WSI variance, thereby decreasing the likelihood of overfit to any one lab (combination of tissue processing, cutting, and staining protocol). All slides were scanned at QEUH using a Phillips Ultra Fast Scanner with resolution equivalent to 40× or, more specifically, 0.25 microns/pixel and stored in the iSyntax file format. WSIs were subsequently converted to OME-Tiffs format using Glencoe Software  [6] to ensure compatibility with QuPath [7] (version v0.2.3) for annotation. The code utilized for this conversion is publicly available from Zenodo  [8]. The annotation procedure involved defining the main slide category, then manually annotating any additional subcategories that could have been available on the WSI.

### Data split to train and test sets

The split percentages were calculated based on the case labels associated with the samples recorded in the system, and the numbers per each set were agreed on by all the data scientist team members and the pathologists. All slides from 2 labs and 10% of randomly selected slides from the other 6 labs were set aside as the test set and never used in the training and validation process. The remaining 90% of the slides, from the 6 other labs, were used as the training set. To retain the same proportion of classes in the train and test sets that were present in the entire original dataset, the dataset was split in a stratified fashion and balanced over categories, subcategories, and staining by different laboratories for the training and test sets. During the annotation process, labels were double-checked by independent pathologists, and in approximately 5% of the cases, the final label associated with the scanned slide differed. This was either because the new slice taken from the biopsy tissue block did not show the same pathological features as the original slide, or the original label had been inaccurately recorded. The corrected labels (i.e., the consensus label agreed on after annotation) were used for training and testing. This means the final number of slides of each type in Table 1 may not match the original proportions of cases selected for study.

**Table 1:** Distribution of samples in training and test sets for the cervical dataset

Category	Subcategory	Count	Training	Test	Total
Malignant	Squamous carcinoma	268	184	81	
	Adenocarcinoma	107	69	38	520
	CGIN	92	60	32	
	Other*	59	44	15	
Total			360	166	
High grade	CIN 2	320	212	108	
	CIN 3	321	221	100	641
Total			433	208	
Low grade	HPV	420	293	127	
	CIN 1	362	253	109	782
Total			546	236	
Normal/inflammation	Normal/inflammation	590	399	131	590
Total			1,738	801	2,539

* Other subcategory represents biopsy specimens with a malignant diagnosis that do not fall under adenocarcinoma or squamous carcinoma. Examples are involvement of the cervix by endometrial tumors or metastases spread from tumors in other parts of the body or other types of malignant tumors that are not carcinoma (e.g., sarcoma).

### Annotation process

Each slide was randomly assigned to 1 of 4 participating consultant pathologists for annotation. Each of the participating pathologists had a subspecialist interest in gynecological pathology and participated in the UK National Gynaecological Pathology External Quality Assurance Scheme. Primary annotation was performed by 1 of the 4 pathologists, or by a biomedical scientist, specifically trained for this project. All annotations done by a biomedical scientist were checked and signed off by one of the study pathologists. When there was a discrepancy, a third reviewer was used and a consensus agreed.

The annotation process stratified slides into 4 main diagnostic categories, which include malignant, high grade, low grade, and normal/inflammation. Each diagnostic category has subcategories. The categories and their subcategories are defined as follows:

1. Malignant: Squamous cell cervical cancer and adenocarcinoma are the most common types of cervical cancer. Both of these are capable of local spread and metastasis. Cervical glandular intraepithelial neoplasia (CGIN) is an uncommon preinvasive dysplastic lesion of glandular cells that can develop into adenocarcinoma (AC) . There is histological overlap with some well-differentiated AC, and this lesion tends to be treated more aggressively.
2. High grade: CIN is graded to determine the risk of developin cancer and to guide further management. Most countries have now moved to a 2-tier classification for CIN (high grade and low grade). In the United Kingdom, pathologists still often refer to the old 3-tier classification (CIN 1/2/3). For the purposes of this algorithm, we classified “high-grade” lesions as those with morphological features of CIN 2 or 3.
3. Low grade: A slide is labeled as low grade if it contains slightly abnormal cells on the surface of the cervix (CIN 1) or low-grade changes that are usually caused by a human papillomavirus (HPV) infection. CIN 1 and HPV are not cancer and usually go away on their own without treatment, but sometimes they can become cancer and spread into nearby tissue.
4. Normal/inflammation: Cervicitis is inflammation of the cervix. Cervicitis is common and may be caused by a number of factors, including infections, chemical or physical irritation, and allergies. Both normal tissue and cervicitis fall within this category, which is not malignant.

A total of 2,539 WSIs, with only 1 slide per patient, in iSyntax format, an annotation file per WSI in JSON format, and a metadata file containing formations about each file, such as categories, subcategories, and staining sites, were delivered at the end of the annotation process.

**Figure 1:**
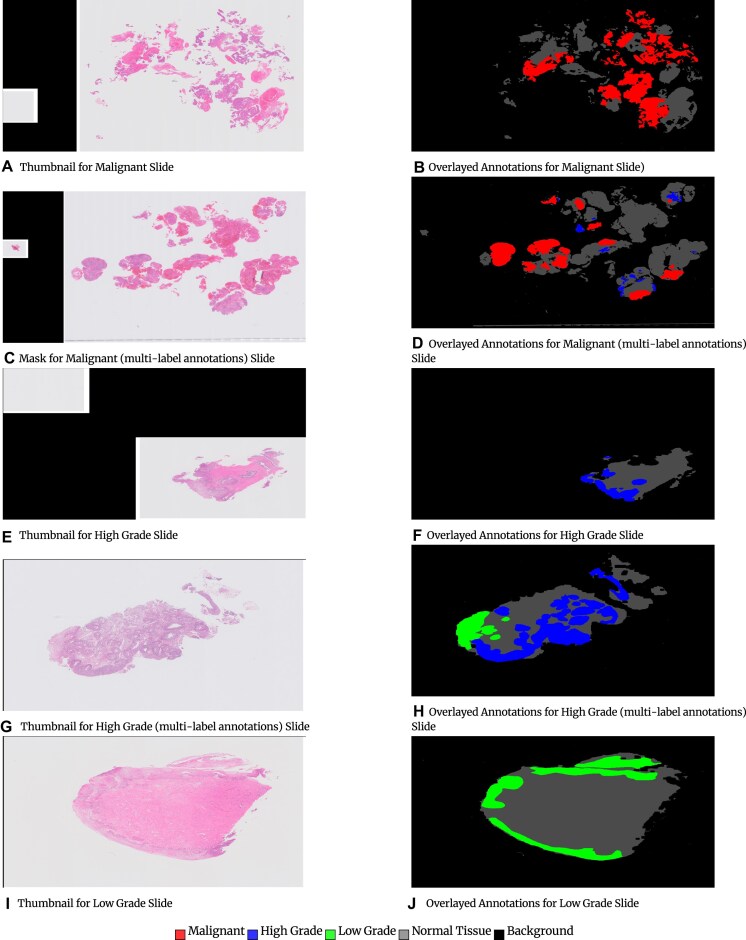
Examples of overlaying annotations on the down-sampled whole-slide images. Malignant high-grade, low-grade, and normal tissue background.

Figure 1 shows examples of overlaying annotations on the thumbnail of the image (downsampled WSIs at level 5) for different categories.

### Interobserver variations

Despite the establishment of strict guidelines by the Bethesda System 2001 (TBS 2001) for reporting cervical smears, intra- and interobserver variations remain unavoidable. These variations are inherent to the diagnostic process and can introduce complexities in training AI models, particularly for grading CIN. CIN represents a morphological continuum, but biopsy specimens are categorized into 2 or 3 distinct grades, making consistency in diagnosis challenging.

To assess interobserver variability, 200 cervical biopsy samples were independently reannotated by 3 pathologists involved in the original annotation process. The results indicate that disagreements among pathologists were more pronounced at the subcategory level, sometimes leading to disagreements in overall category classification  [5].

To evaluate the reliability of annotations, Cohen’s κ statistic was used to measure agreement between observers. For multiple observers, Cohen’s κ was calculated for each pair and averaged. The analysis revealed a Cohen’s κ score of 89.56% for categories and 87.24% for subcategories, indicating a high level of agreement among observers  [5].

## Data Validation and Quality Control

The images submitted were obtained directly from cases undergoing clinical histopathological diagnosis and were subject to rigorous scrutiny by the specialist team of diagnostic histopathologists who undertook the manual annotations of selected features. The diagnosis was available from the original clinical report, and so annotation further confirmed features already commented upon by an expert specialist. Annotations were added afterward as a separate exercise, not linked to primary clinical diagnosis. The gold standard was the pathologists’ diagnosis and when there were discrepancies, by consensus review.

Using the H&E cervical WSI dataset and their annotations, ML algorithms can be applied to assist in various aspects of cervical health analysis. Data collection and preprocessing is the first step in illustrating how ML algorithms can utilize these data, described in  [5].

### Gynaecological Cancer AI project

This dataset, as discussed earlier, was originally created for Gynaecological Cancer AI project, which is part of iCAIRD. The dataset preparation, cleaning and the annotation process of this dataset is described in details in  [5]. In this study, two-thirds of the data described in Table 1 were used as training and validation sets. Table 2 illustrates the number of slides for each subcategory and category in training, validation, and test sets for this study. A patch-level classifier was then trained on the patches extracted from the WSIs in the training set and evaluated on the patches in the validation set. Predictions are probabilities per category for each patch on the slide. A binary heatmap is generated per category per slide using the patch probabilities. Higher probabilities are shown as brighter pixels in a heatmap. The computed probabilities are used to compute the final prediction at the patch level for each slide and to create the patch-level confusion matrices for training and validation datasets. The features extracted from the heatmaps generated at the patch level are used for training an ML classifier to form the final slide-level predictions for each slide.

**Table 2:** Distribution of samples in training, validation, and test sets for a cervical dataset in the iCAIRD Gynaecological Cancer AI project

Category	Subcategory	Count	Training	Validation	Test
Malignant	Squamous carcinoma	268	127	60	81
	Adenocarcinoma	107	243	23	38
	CGIN	92	41	19	32
	Other*	59	29	15	15
High grade	CIN 2	320	141	71	108
	CIN 3	321	146	75	100
Low grade	HPV	420	197	96	127
	CIN 1	362	169	84	109
Normal/inflammation	Normal/inflammation	590	268	191	131
Total		2,539	1,164	574	801

* Other subcategory represents biopsy specimens with a malignant diagnosis that do not fall under adenocarcinoma or squamous carcinoma. Examples are involvement of the cervix by endometrial tumors or metastases spread from tumors in other parts of the body or other types of malignant tumors that are not carcinoma (e.g., sarcoma).

**Figure 2:**
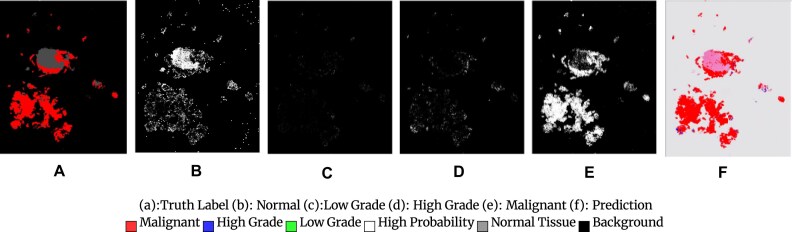
Patch-level heatmaps for a malignant slide. (A) Truth label. (B) Normal. (C) Low grade. (D) High grade. (E) Malignant. (F) Prediction of malignant, high-grade, low-grade, high-probability, and normal tissue background.

Figure 2 shows patch-level generated heatmaps for a malignant slide. The code and the trained models for this project are available at [5, 8].

### Extracting nuclear morphological features using Indica Halo AI

**Figure 3:**
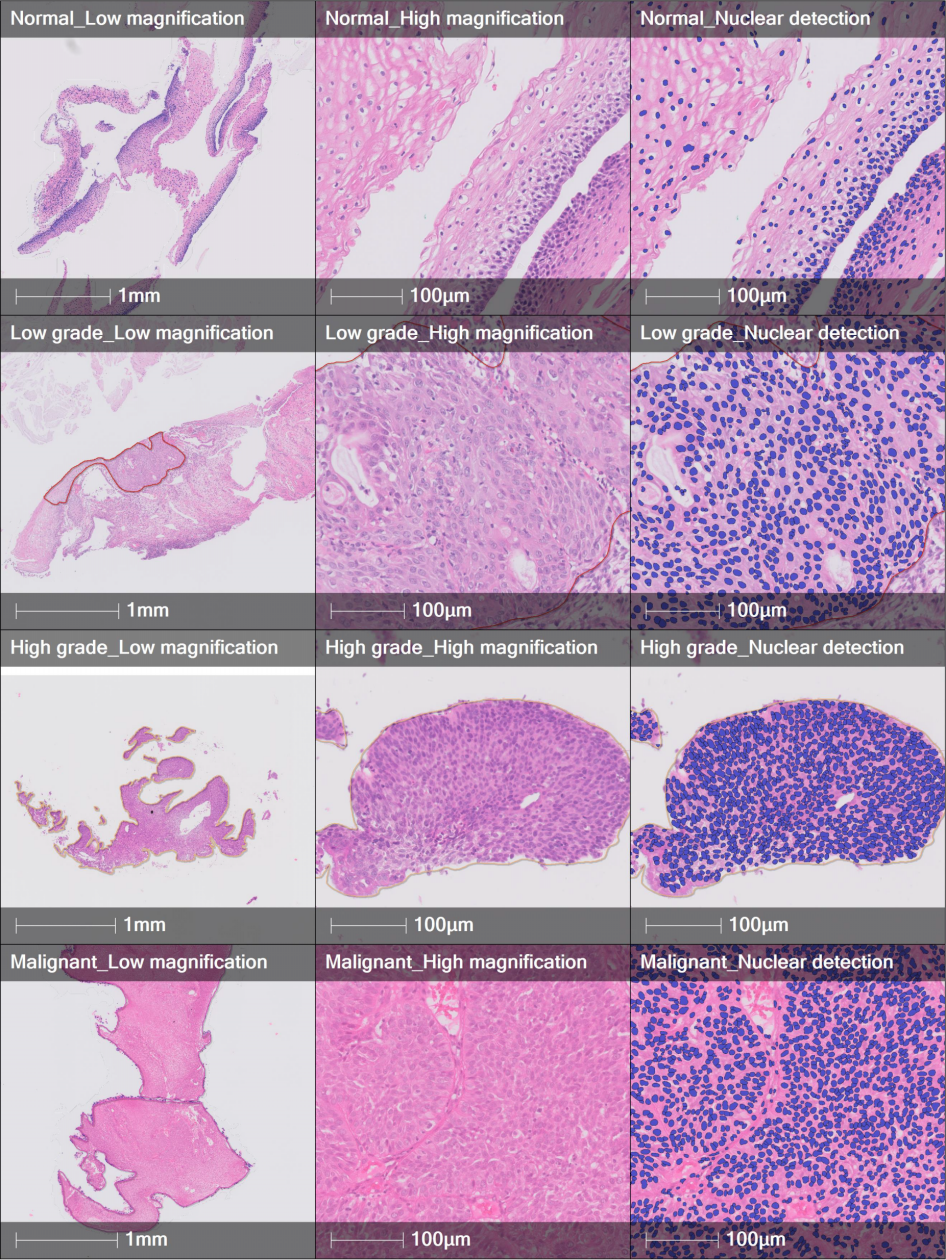
Examples of segmented nuclei (colored blue) of different categories using the Indica HALO AI platform.

In another experiment, WSI images were imported into Indica HALO and HALO AI (v.3.6.4134), along corresponding annotation files. A nuclei segmentation classifier, underpinned by advanced deep learning neural network algorithms, was trained with examples from multiple different cases, as shown in Fig. 3, for different cases. An analysis algorithm, Multiplex IHC v.3.2.3, was utilized to segment individual nuclei to extract nuclear morphological features such as area, perimeter, and roundness within the annotation. The tabular data from the individual nuclear morphological features, along with their x and y coordinates, were exported into CSV file format. A multiplex immunohistochemistry analysis algorithm was used to segment an individual nucleus and to extract its morphological features in 4 different annotations such as normal, low grade, high grade, and malignant.

The data split for this project was done based on the original data split in Table 2, with the only difference that the validation set has been added to training set, and we just have training and test sets for the experiment. Different machine learning algorithms (decision trees, stochastic gradient descent (SGD) optimiser , and random forest classifier) were trained on the morphological features extracted, and finally the trained model was tested with the features extracted from the slides in the test set.

## Reuse potential

In some areas of diagnostic histopathology, large datasets have been made available, particularly in breast and colon cancer, where they have spawned competitions and collaborations to rapidly improve algorithms, as well as provide real-world data for researchers and students. The current dataset is unique and includes a spectrum of histological changes in annotated slides of cervical disease, which can be used for training algorithms, workshops, and validating preexisting algorithms with reference to cervical abnormalities, from normal through dysplasia to carcinoma. The annotations have been applied by expert pathologists and thus can be used for training to identify particular features that are already labeled. Furthermore, inclusion of nuclear morphological features obtained by AI-enabled image analysis may augment accuracy in distinguishing between normal, low-grade, high-grade, and malignant conditions. Cervical disease remains a worldwide problem, and biopsy specimens are frequently small, poorly orientated, and can be a significant part of a laboratory’s clinical workload. Improving accuracy, increasing workflow, and enabling selection of high-risk cases for urgent attention would make a considerable difference to a pathologist’s working day.

## Abbreviations

AC: Adenocarcinoma; AI: artificial intelligence; CIN: cervical intraepithelial neoplasia; CGIN: cervical glandular intraepithelial neoplasia; GB: gigabyte; H&E: hematoxylin and eosin; HPV: human papillomavirus; iCAIRD: Industrial Centre for Artificial Intelligence Research in Digital Diagnostics; ML: machine learning; NG: Glasgow Royal Infirmary; QEUH: Queen Elizabeth University Hospital; RAH: Royal Alexandria Hospital; SG: Southern General Hospital; SGD: stochastic gradient descent; WSI: whole-slide image.

## Ethical Approval

Ethics approval for the study was granted by NHS Greater Glasgow and Clyde Biorepository and Pathology Tissue Resource (REC reference 16/WS/0207) on 4 April 2019. Biorepository approval was obtained (application number 511). Local approval was obtained from the School of Computer Science Ethics Committee, acting on behalf of the University Teaching and Research Ethics Committee (UTREC) [approval code CS15840].

## Competing Interests

The authors declare that they have no competing interests.

## Funding

This work is supported by the Industrial Centre for AI Research in digital Diagnostics (iCAIRD), which is funded by Innovate UK on behalf of UK Research and Innovation (UKRI) [project number: 104690] and in part by Chief Scientist Office, Scotland.

## Author Contributions

M.M. wrote the manuscript and supervised data preprocessing, together with C.F. and I.H.U. P.S. imported annotations in Indica Halo AI platform and measured nuclear morphological features. G.B. initiated the project, and S.B., S.S, and P.K. annotated the whole-slide images. D.H.B. and O.A. supervised machine learning experiments. C.O. arranged data release from the Glasgow Biorepository. J.B. oversaw governance procedures, established digital pathology services, and supervised data deidentification and release. D.H. obtained funding, reviewed results, and helped to draft the manuscript. All authors have seen and approved the manuscript.

## Supplementary Material

giaf144_Authors_Response_To_Reviewer_Comments_Original_Submission

giaf144_Authors_Response_To_Reviewer_Comments_Revision_1

giaf144_GIGA-D-24-00162_Original_Submission

giaf144_GIGA-D-24-00162_Revision_1

giaf144_GIGA-D-24-00162_Revision_2

giaf144_Reviewer_1_Report_Original_Submission Elima Hussain -- 7/22/2024

giaf144_Reviewer_2_Report_Original_Submission Weimiao Yu -- 8/3/2024

giaf144_Reviewer_3_Report_Original_Submission Tristan Lazard -- 8/14/2024

giaf144_Reviewer_3_Report_Revision_1 Tristan Lazard -- 2/27/2025

giaf144_Reviewer_3_Report_Revision_2 Tristan Lazard -- 6/15/2025

## Data Availability

All cervical whole-slide images and their annotation files, binary masks and a metadata file (2,539 images in iSyntax format, 2,539 annotation files in JSON format, 2,539 binary masks in PNG format, and a metadata file in CSV format), and the morphological features extracted from them in Halo are openly available in the BioImage Archive [S-BIAD1168] [9].
