# Supplementary material for: Cervical whole-slide images dataset for multiclass classification
Source: Gigascience. 2025 Nov 29;14:giaf144. doi: 10.1093/gigascience/giaf144 (PMC12751090; doi:10.1093/gigascience/giaf144)
Supplement: giaf144_GIGA-D-24-00162_Revision_1 [file giaf144_giga-d-24-00162_revision_1.pdf]

GigaScience

Cervical Whole Slide Images Dataset for Multi-class Classification

--Manuscript Draft--

**Manuscript Number:**	GIGA-D-24-00162R1
**Full Title:**	Cervical Whole Slide Images Dataset for Multi-class Classification
**Article Type:**	Data Note
**Funding Information:**	Innovate UK (104690) — Prof. David Harrison
**Abstract:**	The clinical pathway for prevention and treatment of cervical cancer depends on cytology and then the assessment of biopsies, fragments of tissue removed for histological examination. This can be a significant workload and is an obvious exemplar to explore triage based on machine learning analysis of slides. Limited access to large annotated datasets of human diseased tissue is a major obstacle to developing standards and algorithms that can assist diagnosis. We present a dataset comprising 2539 whole slide images of cervical biopsies, each annotated by several pathologists and consensus on diagnosis and individual features agreed. Each whole slide image represents one slide per patient, in iSyntax format with manual annotations by pathologists in Jason format. Each whole slide image is assigned a category label which is the final diagnosis of the image, and a subcategory label which declares in which subcategory the image is found. This dataset has been used to build amodel that accurately predicts diagnosis, allowing the possibility of automatically triaging biopsies, so that themost significant pathologies can be identified rapidly and those patients selected for immediate treatment. The level of annotation, at sub-slide level, and the number of cases is unique in public databases and should allow investigators to exploremultiple aspects of computer vision relevant to human tissue diagnosis, with no limitation placed on access to the whole slide images.
**Corresponding Author:**	In Hwa Um, Ph.D.<br>St Andrews University<br>St Andrews, Scotland UNITED KINGDOM
**Corresponding Author Secondary Information:**	
**Corresponding Author's Institution:**	St Andrews University
**Corresponding Author's Secondary Institution:**	
**First Author:**	In Hwa Um, Ph.D.
**First Author Secondary Information:**	
**Order of Authors:**	In Hwa Um, Ph.D.<br>Mahnaz Mohammadi<br>Christina Fell<br>David Morrison<br>Sarah Bell<br>Gareth Bryson<br>Sheeba Syed<br>Prakash Konanahalli<br>Clare Orange

Powered by Editorial Manager® and ProduXion Manager® from Aries Systems Corporation

	Prishma Shahi
	David Harrison
	David Harris-Birtill, PhD
	Ognjen Arandjelovic, PhD
	James D Blackwood
**Order of Authors Secondary Information:**	
**Response to Reviewers:**	Reviewer #2:<br>1.This work is pretty well-structured. Please consider summarizing repetitive information to enhance readability. The abstract is informative but can be more succinct. Focus on the core contributions and potential impacts in fewer words.<br><br>We have amended the abstract to be more succinct as requested.<br><br>2.The annotation process is crucial for the dataset's quality. However, the paper lacks detailed information on the inter-observer variability and consensus-building among pathologists. Including metrics on annotation agreement and methods to resolve discrepancies would strengthen the dataset's reliability.<br><br>We have added a section to explain how we dealt with interobserver variation, based on our original research paper (Mohammadi et al, “Automated reporting of cervical biopsies using artificial intelligence”, https://doi.org/10.1371/journal.pdig.0000381) which presented that Cohen’s kappa (p.11-12, 3.Inter observer variations) was utilised to calculate the score between three observers in discrepant cases and the higher level of agreement was selected.<br><br>3.The details on the selection criteria for pathologists, such years of experience and if they are the specialists, in the annotation process and any training they received to ensure consistency.<br><br>The manuscript summarises relevant details about the annotation team and their qualifications. All histopathologists who annotated biopsies are registered with GMC and Royal College of Pathologists, with subspecialty expertise in gynaecological pathology. Each participating pathologist has a minimum of 5-10 years of post-specialist training experience, with some having over 15 years of expertise in gynaecological pathology. To ensure high-quality and standardised annotations, pathologists reviewed a detailed annotation protocol based on internationally recognised guidelines (WHO classification of tumours, 5th Edition).<br><br>4.The paper mentions various staining protocols and tissue handling methods and such variations are crucial to build a robust model. Discussion on how these variations can be quantified and/or normalized or accounted for during data preprocessing.<br><br>The data from different laboratories were combined before selecting the training set, on the basis as we state, that mixing these slides from different sources minimises the chance of over fitting related to technical differences which are not controllable.<br><br>5.The paper could benefit from a detailed evaluation of the dataset's utility. Including results from preliminary experiments using the dataset to demonstrate its effectiveness in training machine learning models.<br><br>Preliminary results are present in the primary research paper (Mohammadi et al, “Automated reporting of cervical biopsies using artificial intelligence”, https://doi.org/10.1371/journal.pdig.0000381) in which we reported an AI model to assist in the diagnosis of cervical biopsies. The model was trained on a dataset of whole slide images (WSIs) of cervical biopsies, which were annotated by expert pathologists. The AI systems was designed to identify and classify various cervical lesions, aiming to reduce the workload of pathologists by highlighting areas of interest and potentially decreasing the time required for diagnosis. The study demonstrated that the AI model could effectively distinguish between different types of cervical tissue abnormalities, suggesting its potential utility in clinical settings to support pathologists in the diagnostic process. We believe that reference to this paper should help the user

of the dataset to formulate their own plans and experiments.

6.Authors can compare this dataset with existing cervical image datasets. Highlight unique aspects and advantages, such as size, diversity, or annotation quality.

This is the only large study with images of cervical biopsies as opposed to cytological preparations: there is no comparable dataset available as far as we know. We have brought this point out.

7.The authors can include information on the licensing and ethical considerations for using the dataset in research and clinical applications. If this dataset can be used for commercialization purpose? If there is any legal liability of using the given dataset to build the diagnostic models.

As we described in the section “Data Availability”, this dataset can be freely accessed for any purpose without requirement of permission from the authors. It is ethically approved for release and is completely anonymised. As regards the question of legal liability, this is a generic issue, but it is most unlikely that a commercial developer could or would rely on a single dataset upon which to base an application for regulatory approval.

8.Authors can outline and discussion potential future directions for expanding the dataset or improving its annotations.

We are unclear what additional discussion would be valuable. For any clinically useful tool to be developed and implemented, further equivalent datasets would need to be made available.

9.The paper does not address the potential for inter-observer variability in annotations and quality control. This is a critical aspect that can affect the dataset's reliability and should be discussed in detail, while this does not impair the current value of the dataset.

Please see the answers of Q2 and Q3. We believe we have addressed these issues and that pathologists advising any future projects would be very aware of the subjective nature of pathological classification.

10.The technical processes, such as the conversion of image formats and the specifics of machine learning algorithms used, are not sufficiently detailed. Readers would benefit from a deeper understanding of these processes.

The process is described in Mohammadi et al, “Automated reporting of cervical biopsies using artificial intelligence”, https://doi.org/10.1371/journal.pdig.0000381, including how to convert image formats and the specifics of machine learning algorithms. Our experience in discussion with investigators requesting access to data is that they wish to develop their own methodologies depending on the purpose for which they require the data.

11.Understand this is mainly a database paper, but the lack of preliminary results demonstrating the dataset's utility in machine learning applications is a significant omission. Including such results would provide concrete evidence of the dataset's value. If the author has a research paper published, in preparation or archived, can indicate in the manuscripts, which will not affect the sharing this database through a paper in the community.

We already have included Reference 5, which describes the original research which we believe provides a useful framework for other investigators to use this dataset. In our preliminary discussions and sharing drafts with Dr Chris Armit and colleagues, we understood that providing the largest dataset of cervical biopsies without any restrictions as to use would be seen as sufficient value.

12.A comprehensive guideline for researchers on how to use the dataset effectively is very much helpful to increase the impact of this database, including best practices for preprocessing and annotation usage.

In discussions with colleagues, it is clear there is no uniform approach to using data. We believe this is out of scope for a data note and would require consensus across pathology as a discipline. We hope that by providing completely free access to the data that a community will use the information thus inform and refine guidelines and best use principles. That is our primary reason for making these data available.

13.Please ensure that the dataset and any accompanying code are accessible through public repositories. Provide clear instructions for accessing and using the dataset.

This has been done and referenced, following guidance from Dr Chris Armit when he was working with GigaScience.

Reviewer #3:
14.The slides are provided in the iSyntax format, which is not compatible with the OpenSlide library - the most commonly used in the ML community. Offering the slides in a more universal format, such as TIFF, could greatly enhance the accessibility and usability of the dataset for a broader audience.

Glencoe software was used to convert iSyntax format to ome-tiff.
https://www.glencoesoftware.com/blog/2019/12/09/converting-whole-slide-images-to-OME-TIFF.html as described in the text.
In preliminary discussions with Dr Armit, we agreed that although iSYNTAX can be an irritation, it is possible to convert images, and some investigators want access to Philips-originated images precisely to resolve its relative difficulty of accessibility.

15.The proposed dataset-split deliberately places WSIs from the same lab (or at least the same preparation protocol) in both the training and testing sets. I would be interested in understanding the rationale behind this choice, as it could potentially lead to inflated test accuracy.

Please see the answer of reviewer2 -Q4. We believe the mixing actually reduces the chance of bias and overfitting. In addition, a key principle of the ethical approval granted for completely open access, freely available data was its complete de-identifying of images, linking it to individual pathologists or sites. The alternative was a much smaller database being made available or to restrict access. As there is no equivalent set of cases, we felt it was more important to make as large a cohort as possible freely available.

16.The writing in the manuscript could be improved for clarity. At times, the current phrasing makes it challenging to understand the authors' intentions. Below, I've outlined some paragraphs where clarity could be improved:

"Context" Section:
The main point of this paragraph is somewhat unclear. It presents mostly WSI analysis algorithms (presenting in detail two of those works - maybe you could detail why those works are important in this context?). However, because this paper is presenting a new dataset, I would find very useful to present here the other available public datasets of cervical biopsies as well as their sizes and characteristics, for instance.

Another dataset - “Robust whole slide image analysis for cervical cancer screening using deep learning”, Nature communications, 2021 Sep 24; 12(1):1–0, https://doi.org/10.1038/s41467-021-25296-x was described in Mohammadi et al, “Automated reporting of cervical biopsies using artificial intelligence”, https://doi.org/10.1371/journal.pdig.0000381.
17."Data Split to Train and Test Sets" Section:
The sentence, "During the annotation process, these labels were double-checked, and in approximately 5% of the cases, the final label associated with the scanned slide was different," could be clarified. In particular, it is unclear what the initial labels.

We have clarified the text

Powered by Editorial Manager® and ProduXion Manager® from Aries Systems Corporation

18.The phrase, "The corrected labels post-annotation were the labels used for training and testing," would benefit from further clarification. Does this imply that the ML algorithm (« training and testing.. ») was trained before the slides were fully annotated? The reference to "[…] described above" is somewhat vague. To which percentages are you referring to ?

We have clarified this section

19."Annotation Process" Section:
I understand from this section that each slide is assigned both a main label and a sub-label. If pixel-level annotation masks are also available, it would be useful to provide additional details on this process also. The statement, "Primary annotation was performed either by one of the four pathologists or by a biomedical scientist specifically trained for this project," seems to conflict with the earlier mention that each slide is randomly assigned to one of the four pathologists. Additionally, it would be helpful to clarify whether the "primary annotation" is the same as the "main label" mentioned later. If so, further information on who performed the sub-category annotations would be valuable.

We have attempted to clarify this. First annotations were undertaken by expert biomedical scientists. Then they were allocated randomly to the pathologists to review the annotations. If there was no consensus a third review was undertaken. Histopathology is by its very essence subjective. By having multiple reviews and consensus we have tried to minimise variation.

20.Including the identity of the annotator (encoded as a discrete value) in the metadata could be beneficial, as it might help account for this potential source of bias.

Discussion of interobserver variation has been improved. As discussed above, we were encouraged by the ethics committee to release minimum information that might lead to identifiability. We made a judgement call that having a freely available large dataset outweighed more personalised information about reviewers, even though it would have been anonymous. We have attempted to reduce bias by always ensuring there is a consensus diagnosis from at least two observers, rather than relying on a single observer.

21."Data Validation and Quality Control" Section:
This paragraph could be clearer, particularly the sentence: "The gold standard was the pathologists' diagnosis and where there was discrepancies, by consensus review. » I did not understand it.

We have altered wording.

22.The relevance of the subsequent paragraphs, "Gynaecological Cancer AI Project" and "Extracting Nuclear Morphological Features Using Indica Halo AI," is not clear to me. In the former, the algorithm is described with insufficient detail for reproduction or comprehension ("The features extracted from the heatmaps generated at the patch level are used for training an ML classifier to form the final slide-level predictions for each slide." What final ML classifier is used? Which features are extracted from the heatmaps?). In the latter, while the idea of extracting nuclear morphology per pathology class is promising, it's unclear why it is described here. Do you provide this extraction? What are the performance metrics for the ML models trained on these features?

The specific questions are addressed in reference 5. We have attempted to clarify the text.

23.Other Remarks:
There are some minor errors in the text (e.g., "the achieves of the Glasgow Royal Infirmary," or nested parentheses errors like "(low grade (including …()") that could benefit from careful proofreading.
Some figures, such as "architecture.png," are of very low quality and could be improved to better support the manuscript.

Powered by Editorial Manager® and ProduXion Manager® from Aries Systems Corporation

	Thank you for drawing our attention to these points
**Additional Information:**	
**Question**	**Response**
Are you submitting this manuscript to a special series or article collection?	No
**Experimental design and statistics**<br><br>Full details of the experimental design and statistical methods used should be given in the Methods section, as detailed in our Minimum Standards Reporting Checklist. Information essential to interpreting the data presented should be made available in the figure legends.<br><br>Have you included all the information requested in your manuscript?	Yes
**Resources**<br><br>A description of all resources used, including antibodies, cell lines, animals and software tools, with enough information to allow them to be uniquely identified, should be included in the Methods section. Authors are strongly encouraged to cite Research Resource Identifiers (RRIDs) for antibodies, model organisms and tools, where possible.<br><br>Have you included the information requested as detailed in our Minimum Standards Reporting Checklist?	Yes
**Availability of data and materials**<br><br>All datasets and code on which the conclusions of the paper rely must be either included in your submission or deposited in publicly available repositories (where available and ethically appropriate), referencing such data using a unique identifier in the references and in the "Availability of Data and Materials"	Yes

section of your manuscript.<br><br>Have you have met the above requirement as detailed in our Minimum Standards Reporting Checklist?	

```
This is pdfTeX, Version 3.141592653-2.6-1.40.26 (TeX Live 2024)
(preloaded format=pdflatex 2024.8.2)  13 FEB 2025 11:10
entering extended mode
 restricted \write18 enabled.
 %&-line parsing enabled.
**"main cervical.tex"
(./main cervical.tex
LaTeX2e <2024-06-01> patch level 2
L3 programming layer <2024-05-27>

! LaTeX Error: File `oup-contemporary.cls' not found.

Type X to quit or <RETURN> to proceed,
or enter new name. (Default extension: cls)

Enter file name:
! Emergency stop.
<read *>

l.11 ^^M

*** (cannot \read from terminal in nonstop modes)


Here is how much of TeX's memory you used:
 19 strings out of 473583
 482 string characters out of 5732343
 1925908 words of memory out of 5000000
 23012 multiletter control sequences out of 15000+600000
 558069 words of font info for 36 fonts, out of 8000000 for 9000
 1141 hyphenation exceptions out of 8191
 19i,0n,29p,106b,17s stack positions out of
10000i,1000n,20000p,200000b,200000s
!  ==> Fatal error occurred, no output PDF file produced!
```

Click here to access/download;Manuscript;Final Cervical_Data_Note_GigaScience-final-13Feb25.pdf

GigaScience, 2023, 1–7

doi: xx.xxxx/xxxx
Manuscript in Preparation
Data Note

DATA NOTE

Cervical Whole Slide Images Dataset for Multi-class Classification

Mahnaz Mohammadi[1,*,§], Christina Fell[1,*], David Morrison[1,*], Sarah Bell[2,†], Gareth Bryson[2,†], Sheeba Syed[2,†], Prakash Konanahalli[2,†], David Harris-Birtill[4,*], Ognjen Arandjelovic[4,*], Clare Orange[1,2,*], Prishma Shahi[1,*], In Hwa Um[1,3,*,§], James D Blackwood[1,*] and David J Harrison[1,3,*]

[1]School of Medicine, University of St Andrews, North Haugh, KY16 9TF, United Kingdom and [2]Department of Pathology, Queen Elizabeth University Hospital, Govan Road, G51 4TF, Glasgow, United Kingdom and [3]Pathology, Division of Laboratory Medicine, Royal Infirmary of Edinburgh, Old Dalkeith Road, EH16 4SA, United Kingdom and [4]School of Computer Science, University of St Andrews, North Haugh, KY16 9SX, United Kingdom

[*]mm459, cmf21, dm236, dcchb, oa7, celo1, ps289, ihu, jdb20, david.harrison@st-andrews.ac.uk
[†]Sarah.Bell, Gareth.Bryson, sheeba.syed, prakash.konanahalli@ggc.scot.nhs.uk
[§]Corresponding author: mahnaz.mohammadi0@gmail.com, ihu@st-andrews.ac.uk

Abstract

The clinical pathway for prevention and treatment of cervical cancer depends on cytology and then the assessment of biopsies, fragments of tissue removed for histological examination. This can be a significant workload and is an obvious exemplar to explore triage based on machine learning analysis of slides. Limited access to large annotated datasets of human diseased tissue is a major obstacle to developing standards and algorithms that can assist diagnosis. We present a dataset comprising 2539 whole slide images of cervical biopsies, each annotated by several pathologists and consensus on diagnosis and individual features agreed. Each whole slide image represents one slide per patient, in iSyntax format with manual annotations by pathologists in Jason format. Each whole slide image is assigned a category label which is the final diagnosis of the image, and a subcategory label which declares in which subcategory the image is found. This dataset has been used to build a model that accurately predicts diagnosis, allowing the possibility of automatically triaging biopsies, so that the most significant pathologies can be identified rapidly and those patients selected for immediate treatment. The level of annotation, at sub-slide level, and the number of cases is unique in public databases and should allow investigators to explore multiple aspects of computer vision relevant to human tissue diagnosis, with no limitation placed on access to the whole slide images.

Key words: Whole slide imaging; histopathology; cervix; cervical cancer; digital image database; machine learning; deep learning; healthcare dataset.

Data Description

This large collection of cervical Whole Slide Image (WSI) data serves as a vital resource to improve cervical cancer detection, diagnosis, and research, ultimately contributing to the overall goal of reducing the burden of this preventable and treatable disease on a global scale. Cervical WSI data provides detailed, high-resolution views of cervical tissue samples, enabling healthcare providers to detect abnormalities and precancerous lesions with greater precision. This improved accuracy can lead to earlier intervention and improved patient outcomes. Researchers and data scientists utilize this comprehensive dataset to develop and fine-tune Machine Learning (ML) algorithms and Artificial Intelligence (AI) tools. These tools can automate and streamline the screening process, making it more

Compiled on: February 13, 2025.
Draft manuscript prepared by the author.

accessible and cost-effective, particularly in regions with limited healthcare resources.

The dataset consists of a total of 2539 Haematoxylin and Eosin (H&E) cervical WSIs, with one slide per patient, in iSyntax format requiring 731 Giga Bytes (GB) storage. This dataset was originally created for Gynaecological Cancer AI project which is part of Industrial Centre for Artificial Intelligence Research in Digital Diagnostics (iCAIRD) [1] and was used to develope and train AI for diagnosis of cervical biopsies as either benign dysplastic (pre-cancerous), or neoplastic (cancer). For abnormal biopsies, classification was as: invasive squamous or adeno-carcinoma, intraepithelial neoplasia (low grade (including HP and CIN 1 and high grade (including CIN 2 and CIN 3).

Context

As the demand for AI tools for diagnosis continues to grow, so does the need for high-quality datasets. Datasets are a critical component of AI development as they provide the training data that enables the ML models to learn patterns, relationships, and make prediction. Datasets can be used for training, evaluation and testing different ML models. They also can serve as benchmarks for comparing different algorithms and models. Datasets contribute to the advancement of ML research by providing a foundation for exploring new algorithms, techniques, and models. Hence, quality, diversity, representativeness, size, balance, and potential biases of the dataset are factors that can significantly impact the performance and generalization of ML applications.

Digital pathology offers several benefits and addresses various challenges in traditional pathology practice, making it a valuable and increasingly essential component of modern healthcare. AI has become increasingly important in the field of digital pathology due to its potential to revolutionize the way medical professionals analyse and interpret pathology slides. AI can help identify patterns that may be difficult to spot with the human eye, leading to faster and more accurate diagnoses. It can aid in the early detection of diseases like cancer, by analysing subtle changes in images over time which can lead to earlier interventions and improved patient outcomes.

Histopathology is the microscopic examination of tissue samples to diagnose diseases and understand their underlying causes. Histopathology WSIs are indispensable resources in the field of medical image analysis and ML applications. Staining techniques are essential in histopathology to enhance the visualization of cellular structures and specific components within tissues. Different staining techniques are used to highlight various tissue elements and help pathologists differentiate between normal and abnormal structures. The digital representations of tissue samples stained with Hematoxylin and Eosin (H&E) provide a comprehensive view of cellular and tissue structures at a microscopic level. The color contrast given to different cell types and tissue components, aids pathologists in diagnosing and characterizing diseases and provides rich source of visual and contextual information, making them an ideal input for various ML tasks.

ML algorithms trained on a large dataset of annotated H&E WSIs, can be useful in diagnostic assistance, predicting disease progression, drug discovery and development, educational tools and many more. Trained ML algorithms on WSIs, can assist pathologists in diagnosing diseases, identify patterns and anomalies in tissue samples and consequently lead to more accurate and efficient diagnoses. Detecting and segmenting tumor regions within H&E WSIs is another example which aids in quantifying tumor size, density, and distribution, which are essential factors in disease prognosis and treatment planning. Meaningful features extracted from H&E WSIs, such as texture, shape, and color information can be used to characterize tissue structures, helping researchers and clinicians understand tissue composition and potentially uncover new insights.

A comprehensive survey of cervical histopathology image analysis using machine vision approaches is presented [2]. This paper reviews all the related works of cervical histopathology image analysis using machine vision techniques from 1988 to 2020. In this survey, more than 60 related works are summarized from 1988 to 2019. Authors of [3] propose a GBUL approach to describe the topological information of different tissues in the histopathological images and two stages of unsupervised leaning processes are applied to group the tissues into relevant types. A weakly supervised survival convolutional neural network approach equipped with a visual attention mechanism for predicting overall survival is presented in [4]. The inclusion of visual attention provides insights into regions of the tumor microenvironment with the pathological interpretation which may improve understanding of the disease pathomechanism. This analysis is performed on two independent, multi-center patient data sets of lung (which is publicly available data) and bladder urothelial carcinoma. The presented results highlight the significance of computational pathology algorithms for predicting prognosis using H&E stained images alone and underpin the use of computational methods to improve the efficiency of clinical trial studies.

In gynaecological cancer AI project in iCAIRD, Artificial Intelligence (AI) algorithms were trained and evaluated on cervical biopsies for automated reporting of digital diagnostics with aim is to increase overall efficiency of pathological diagnosis and to have the performance tuned to high sensitivity for malignant cases. The algorithms were trained and validated on 1738 cervical WSIs. On the independent test set of 811 WSIs, the trained algorithm achieved 93.4% malignant sensitivity for classifying slides [5].

Methods

Data collection

The cervical tissue blocks were originally collected from the achieves of Glasgow Royal Infirmary (NG), Southern General Hospital (SG), Royal Alexandria Hospital (RAH) and Queen Elizabeth University Hospital (QEUH) (all in Glasgow, Scotland) each with independent tissue handling including fixation and tissue processing. The number of tissue blocks obtained from each of the above sites were: 829 from QEUH, 729 from NG, 647 from SG and 334 from RAH. Since some of these slides were faded new tissue sections were cut from the tissue blocks at one of two different thicknesses (3 microns or 4 microns) and then stained with one of four different H&E protocols (routine H&E, muscle biopsy protocol, neuro protocol and paediatric tissues protocol). Together, these combinations gave eight different labs maximising WSI variance and thereby decrease the likelihood of overfit to any one lab (combination of tissue processing, cutting and staining protocol). All the slides were scanned at QEUH using a Phillips Ultra Fast Scanner (UFS) with resolution equivalent to 40x or more specifically 0.25 microns/pixel, and stored in the iSyntax file format. WSIs were then converted to OME-Tiffs using Glencoe Software [6] to make them compatible with QuPath [7] (Version v0.2.3) for annotation. The annotation procedure involved defining the main slide category, then manually annotating any additional subcategories that could have been available on the WSI.

Data split to train and test sets

The split percentages were calculated based on the case labels associated with the samples recorded in the system and the numbers per each set were agreed on by all the data scientist team members and the pathologists. All slides from two of the labs and 10% randomly selected slides from the other six labs were set aside as test set and never used in the training and validation process. The remaining 90% of the slides, from the 6 other labs were used as training set. To retain the same proportion of classes in the train and test sets

Table 1. Distribution of samples in training and test sets for cervical dataset.

Category	SubCategory	Count	Training	Test	Total
Malignant	- Squamous carcinoma	268	184	81	
	- Adenocarcinoma	107	69	38	520
	- CGIN	92	60	32	
	- Other*	59	44	15	
	Total		360	166	
High Grade	- CIN 2	320	212	108	
	- CIN 3	321	221	100	641
	Total		433	208	
Low Grade	- HPV	420	293	127	
	- CIN 1	362	253	109	782
	Total		546	236	
Normal/inflammation	- Normal/inflammation	590	399	131	590
	Total		1738	801	2539

* Other subcategory in malignant are biopsies with a malignant diagnosis that don't fall under adenocarcinoma or squamous carcinoma. Examples are involvement of the cervix by endometrial tumours or metastases spread from tumours in other parts of the body or other types of malignant tumour that are not carcinoma (e.g. sarcoma).

that were present in the entire original dataset, the dataset was split in stratified fashion balanced over categories, subcategories and staining by different laboratories for training and test sets. During the annotation process labels were doubl-checked and by independent pathologists in approximately 5% of the cases the final label associated with the scanned slide differed. This was either because the new slice taken from the biopsy tissue block did not show the same pathological features as the original slide, or that the original label had been inaccurately recorded. The corrected labels, that is the consensus label agreed after annotation were used for training and testing. This means the final numbers of slides of each type in Table 1 may not match the original proportions of cases selected for study.

Annotation process

Each slide was randomly assigned to one of four participating Consultant Pathologists for annotation. Each of the participating pathologists had a sub-specialist interest in Gynaecological Pathology, and participated in the UK National Gynaecological Pathology External Quality Assurance Scheme. Primary annotation was performed either by one of the four pathologists, or by a biomedical scientist, specifically trained for this project. All annotations done by a biomedical scientist were checked and signed off by one of the study pathologists. Where there was discrepancy, a third reviewers was used and a consensus agreed.

The annotation process stratified slides into four main diagnostic categories which include malignant, high grade, low-grade and Normal/inflammation. Each diagnostic category has subcategories. The categories and their sub-categories are defined as follows:

i. Malignant: Squamous cell cervical cancer and adenocarcinoma are the most common types of cervical cancer. Both of these are capable of local spread and metastasis. CGIN is an uncommon pre-invasive dysplastic lesion of glandular cells which can develop into AC. There is histological overlap with some well differentiated AC and this lesion tends to be treated more aggressively.
ii. High Grade: CIN is graded to determine risk of development of cancer and to guide further management. Most countries have now moved to a two tier classification for CIN (high grade and low grade). In the UK, pathologists still often refer to the old three tier classification (CIN1/2/3). For the purposes of this algorithm, we classified 'high grade' lesions as those with morphological features of CIN 2 or 3.
iii. Low Grade: A slide is labelled as low grade if it contains slightly abnormal cells on the surface of the cervix (CIN 1) or low-grade changes that are usually caused by an HPV infection (HPV). CIN 1 and HPV are not cancer and usually go away on their own without treatment, but sometimes they can become cancer and spread into nearby tissue.
iv. Normal/inflammation: Cervicitis is inflammation of the cervix. Cervicitis is common and may be caused by a number of factors, including infections, chemical or physical irritation, and allergies. Both normal tissue and cervicitis fall within this category which is not malignant.

Total of 2539 whole slide images (WSIs), with only one slide per patient, in iSyntax format, an annotation file per WSI in JSON format and a metadata file containing formations about each files, such as categories, subcategories, staining sites, etc was delivered at the end of the annotation process.

Figure 1 shows examples of overlaying annotations on the thumbnail of the image (downsampled whole slide images at level 5) for different categories.

Inter-observer variations

Despite the establishment of strict guidelines by the Bethesda System 2001 (TBS 2001) for reporting cervical smears, intra- and inter-observer variations remain unavoidable. These variations are inherent to the diagnostic process and can introduce complexities in training AI models, particularly for grading Cervical Intraepithelial Neoplasia (CIN). CIN represents a morphological continuum, but biopsies are categorised into two or three distinct grades, making consistency in diagnosis challenging .

To assess inter-observer variability, 200 cervical biopsy samples were independently re-annotated by three pathologists involved in the original annotation process. The results indicate that disagreements among pathologists were more pronounced at the subcategory level, sometimes leading to disagreements in overall category classification [5].

To evaluate the reliability of annotations, Cohen's kappa statistic was used to measure agreement between observers. For multiple observers, Cohen's kappa was calculated for each pair and averaged. The analysis revealed a Cohen's kappa score of 89.56 % for categories and 87.24% for subcategories, indicating a high level of agreement among observers [5].

Data validation and quality control

The images submitted were obtained directly from cases undergoing clinical histopathological diagnosis and were subject to rigorous scrutiny by the specialist team of diagnostic histopathologists who undertook the manual annotations of selected features. The diagnosis was available from the original clinical report and so annotation was a further confirmation of features already commented upon by an expert specialist. Annotations were added afterwards as a

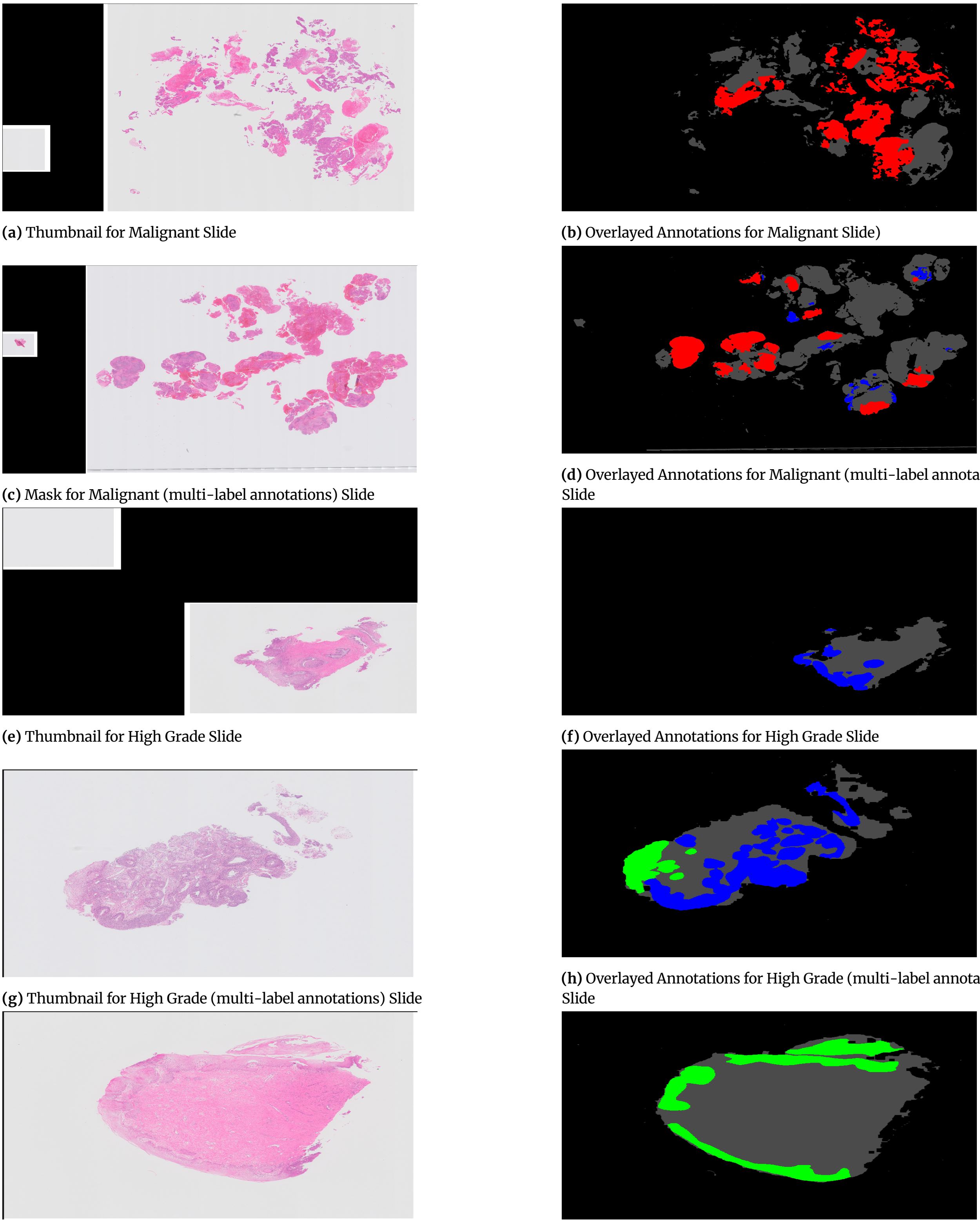

(a) Thumbnail for Malignant Slide

(b) Overlayed Annotations for Malignant Slide)

(c) Mask for Malignant (multi-label annotations) Slide

(d) Overlayed Annotations for Malignant (multi-label annotations) Slide

(e) Thumbnail for High Grade Slide

(f) Overlayed Annotations for High Grade Slide

(g) Thumbnail for High Grade (multi-label annotations) Slide

(h) Overlayed Annotations for High Grade (multi-label annotations) Slide

(i) Thumbnail for Low Grade Slide

(j) Overlayed Annotations for Low Grade Slide

Figure 1. Examples of overlaying annotations on the down-samples whole slide images

Malignant High Grade Low Grade Normal Tissue Background

separate exercise, not linked to primary clinical diagnosis. The gold standard was the pathologists' diagnosis and where there were discrepancies, by consensus review.

Using H&E cervical WSI dataset and their annotations, ML algorithms can be applied to assist in various aspects of cervical health analysis. Data collection and preprocessing is the first step in illustrating how ML algorithms can utilise this data, described in [5].

Gynaecological Cancer AI project

This dataset as discussed earlier was originally created for Gynaecological Cancer AI project which is part of iCAIRD. The dataset

Table 2. Distribution of samples in training, validation, and test sets for cervical dataset in iCAIRD gynaecological cancer AI project.

Category	SubCategory	Count	Training	Validation	Test
Malignant	- Squamous carcinoma	268	127	60	81
	- Adenocarcinoma	107	243	23	38
	- CGIN	92	41	19	32
	- Other*	59	29	15	15
High Grade	- CIN 2	320	141	71	108
	- CIN 3	321	146	75	100
Low Grade	- HPV	420	197	96	127
	- CIN 1	362	169	84	109
Normal /inflammation	- Normal /inflammation	590	268	191	131
	Total	2539	1164	574	801

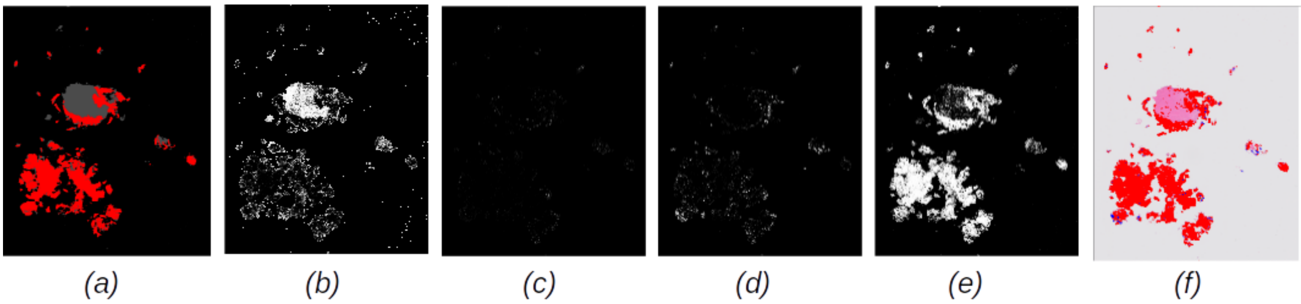

Figure 2. Patch level heatmaps for a malignant slide
(a):Truth Label (b): Normal (c):Low Grade (d): High Grade (e): Malignant (f): Prediction
Malignant High Grade Low Grade High Probability Normal Tissue Background

preparation, cleaning and the annotation process of this dataset is described in details in [5]. In this study two thirds of the data described in Table 1 was used as training and validation set. Table 2 illustrates the number of slides for each subcategory and category in training, validation and test sets for this study. A patch-level classifier was then trained on the patches extracted from the WSIs in the training set and evaluated on the patches in the validation set. Predictions are probabilities per category for each patch on the slide. A binary heatmap is generated per category per slide using the patch probabilities. Higher probabilities are shown as brighter pixels in a heatmap. The computed probabilities are used to compute the final prediction at patch level for each slide and to create the patch level confusion matrices for training and validation datasets. The features extracted from the heatmaps generated at patch level are used for training a ML classifier to form the final slide level predictions for each slide.

Figure 2 shows patch level generated heatmaps for a malignant slide. The code and the trained models for this project is available at [8].

Extracting nuclear morphological features using Indica Halo AI

In another experiment, WSI images were imported into Indica HALO and HALO AI (v.3.6.4134), along with corresponding annotation files. A nuclei segmentation classifier, underpinned by advanced deep learning neural network algorithms, was trained with examples from multiple different cases as shown in Figure 3 for different cases. An analysis algorithm, Multiplex IHC v.3.2.3 was utilised to segment individual nuclei to extract nuclear morphological features such as area, perimeter, and roundness within the annotation. The tabular data from the individual nuclear morphological features, along with their x and y coordinates, was exported into CSV file format. Multiplex IHC analysis algorithm was used to segment individual nucleus and to extract its morphological features in four different annotations such as normal, low grade, high grade, and malignant. The data split for this project was done based on the original data split in Table 2 with only difference that the validation set has been added to training set and we just have training and test sets for the experiment. Different machine learning algorithms (Decision Trees, SGD and Random Forest classifier) were trained on the morphological features extracted and finally the trained model was tested with the features extracted from the slides in the test set.

Re-use potential

In some areas of diagnostic histopathology large datasets have been made available, particularly in breast and colon cancer, where they have spawned competitions and collaborations to rapidly improve algorithms as well as providing real world data for researchers and students. The current dataset is unique, and includes a spectrum of histological changes in annotated slides of cervical disease, that can be used for training algorithms, workshops and validation of pre-existing algorithms with reference to cervical abnormalities, from normal, through dysplasia to carcinoma. The annotations have been applied by expert pathologists and thus can be used for training to identify particular features that are already labelled. Furthermore, inclusion of nuclear morphological features obtained by AI-enabled image analysis may augment accuracy in distinguishing between normal, low-grade, high-grade, and malignant conditions. Cervical disease remains a worldwide problem and biopsies are frequently small, poorly orientated and can be a significant part of a laboratory's clinical workload. Improving accuracy, increasing workflow and enabling selection of high risk cases for urgent attention would make a considerable difference to a pathologist's working day.

Data availability

All cervical whole slide images, their annotation files, binary masks and a metadata file (2539 images in iSyntax format, 2539 annotation files in JSON format, 2539 binary masks in PNG format and a metadata file in CSV format) and the morphological features extracted from them in Halo are openly available in the GigaScience

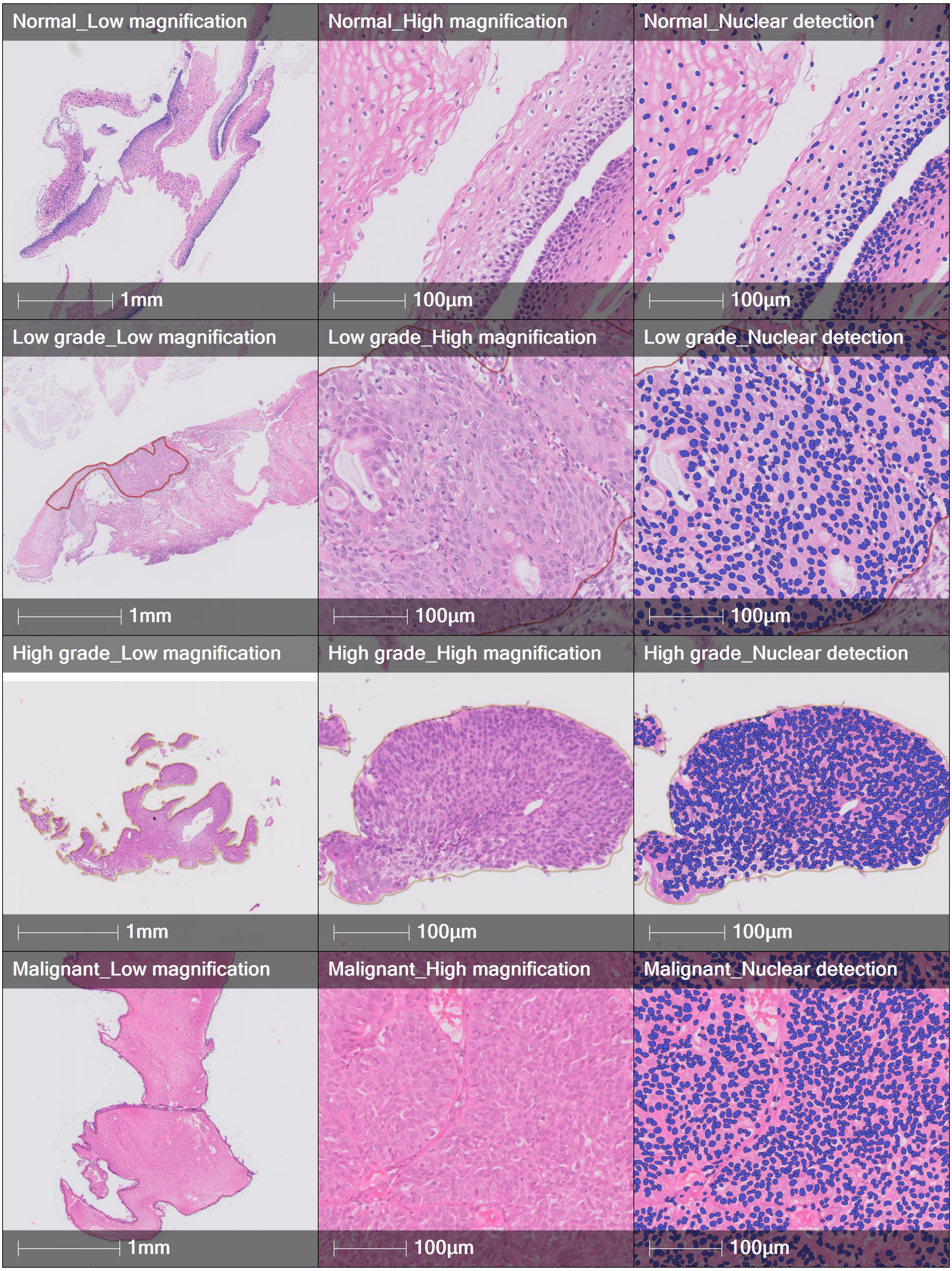

Figure 3. Examples of segmented nuclei (colored blue) of different categories using Indica HALO AI platform

repository, GigaDB [S-BIAD1168] [9].

Declarations

List of abbreviations

- Artificial Intelligence (AI)
- Cervical Intraepithelial Neoplasia (CIN)
- Cervical Glandular Intraepithelial Neoplasia (CGIN)
- Giga Byte (GB)
- Graph Based Unsupervised Learning (GBUL)
- Human Papilloma Virus (HPV)
- Industrial Centre for Artificial Intelligence Research in Digital Diagnostics (iCAIRD)
- Machine Learning (ML)
- Quantitative Pathology (QuPath)
- Whole Slide Image (WSI)

Ethical Approval

- Ethics approval for the study was granted by NHS Greater Glasgow and Clyde Biorepository and Pathology Tissue Resource (REC reference 16/WS/0207) on 4th April 2019.
- Biorepository approval was obtained (application number 511)
- Local approval was obtained from the School of Computer Science Ethics Committee, acting on behalf of the University Teaching and Research Ethics Committee (UTREC) [Approval code-CS15840].

Consent for publication

Not applicable.

Competing Interests

The authors declare that they have no competing interests.

Funding

This work is supported by the Industrial Centre for AI Research in digital Diagnostics (iCAIRD) which is funded by Innovate UK on behalf of UK Research and Innovation (UKRI) [project number: 104690], and in part by Chief Scientist Office, Scotland.

Author's Contributions

Mahnaz Mohammadi wrote the manuscript and supervised data preprocessing, together with Christina Fell and In Hwa Um. Prishma Shahi imported annotations in Indica Halo AI platform and measured nuclear morphological features.
Gareth Bryson initiated the project, and Sarah Bell, Sheeba Syed, and Prakash Konanahalli annotated the whole slide images. David Harris Birtill and Ognjen Arandjelovic supervised machine learning experiments.
Clare Orange arranged data release from Glasgow Biorepository. James Blackwood oversaw governance procedures, established digital pathology services and supervised data de-identification and release. David Harrison is Director of iCAIRD, obtained funding, reviewed results and helped to draft the manuscript. All authors have seen and approved the manuscript.

Acknowledgements

We acknowledge the support of NHS Research Scotland (NRS) Greater Glasgow and Clyde Biorepository. We acknowledge the support of the biomedical scientists, Tim Prosser, Lucy Irving, Jennifer Campbell and Jennifer Faulkner, from the Pathology Department, NHS Greater Glasgow and Clyde for technical support.

Authors' information

MM, CF, and DM hold a PhD degree and are data scientists in the School of Medicine, University of St Andrews.
PS is a research technician at the School of Medicine, University of St Andrews.
Dr Gareth Bryson is a Consultant Pathologist and Clinical Director for Laboratory Medicine at the Queen Elizabeth University Hospital, Glasgow. Drs Sarah Bell, Prakash Konanahalli and Sheeba Syed are consultant gynaecological pathologists at Queen Elizabeth University Hospital, NHS Greater Glasgow and Clyde, UK. Ognjen Arandjelovic and David Harris Birtill are Reader and Senior Lecturer respectively in Computer Science, University of St Andrews.
In Hwa Um is a postdoctoral research fellow in pathology AI in the University of St Andrews. Clare Orange is Biorepository Manager in NHS Greater Glasgow and Clyde, and a doctoral candidate in the University of St Andrews.
James Blackwood is Chief Technical Officer of iCAIRD and Innovation Fellow in University of St Andrews. David Harrison is Professor of Pathology at the University of St Andrews, and Director of iCAIRD.

References

1. Gynaecological Cancer AI.;. https://icaird.com/wp9-gynaecological-cancers/.
2. Li C, Chen H, Li X, Xu N, Hu Z, Xue D, et al. A review for cervical histopathology image analysis using machine vision approaches. Artificial Intelligence Review 2020;53:4821–4862.
3. Li C, Hu Z, Chen H, Ai S, Zhang J, Zhang Y, et al. A cervical histopathology image clustering approach using graph based features. SN Computer Science 2021;2:1–20.
4. Qaiser T, Lee CY, Vandenberghe M, Yeh J, Gavrielides MA, Hipp J, et al. Usability of deep learning and H&E images predict disease outcome-emerging tool to optimize clinical trials. NPJ precision oncology 2022;6(1):37.
5. Mohammadi M, Fell C, Morrison D, Syed S, Konanahalli P, Bell S, et al. Automated reporting of cervical biopsies using artificial intelligence. PLOS Digital Health 2024;3(4):e0000381.
6. Mellisa Linkert, Chris Allan, Converting Whole Slide Images to OME-TIFF: A New Workflow; 2019. https://www.glencoesoftware.com/blog/2019/12/09/converting-whole-slide-images-to-OME-TIFF.html, Last accessed on 2022-08-12.
7. Bankhead P, Loughrey MB, Fernández JA, Dombrowski Y, McArt DG, Dunne PD, et al. QuPath: Open source software for digital pathology image analysis. Scientific reports 2017;7(1):1–7.
8. Fell C, Mohammadi M, Morrison D. StAndrewsMedTech/icairdpath-public: Release for publication 2023 Feburary;https://zenodo.org/record/7674764.
9. In Hwa Um CFDMSBGBSSPKCOPS Mahnaz Mohammadi, Harrison D, Cervical Whole Slide Images Dataset ;.

Click here to access/download;Figure;IC-CX-00027-01_mask.png

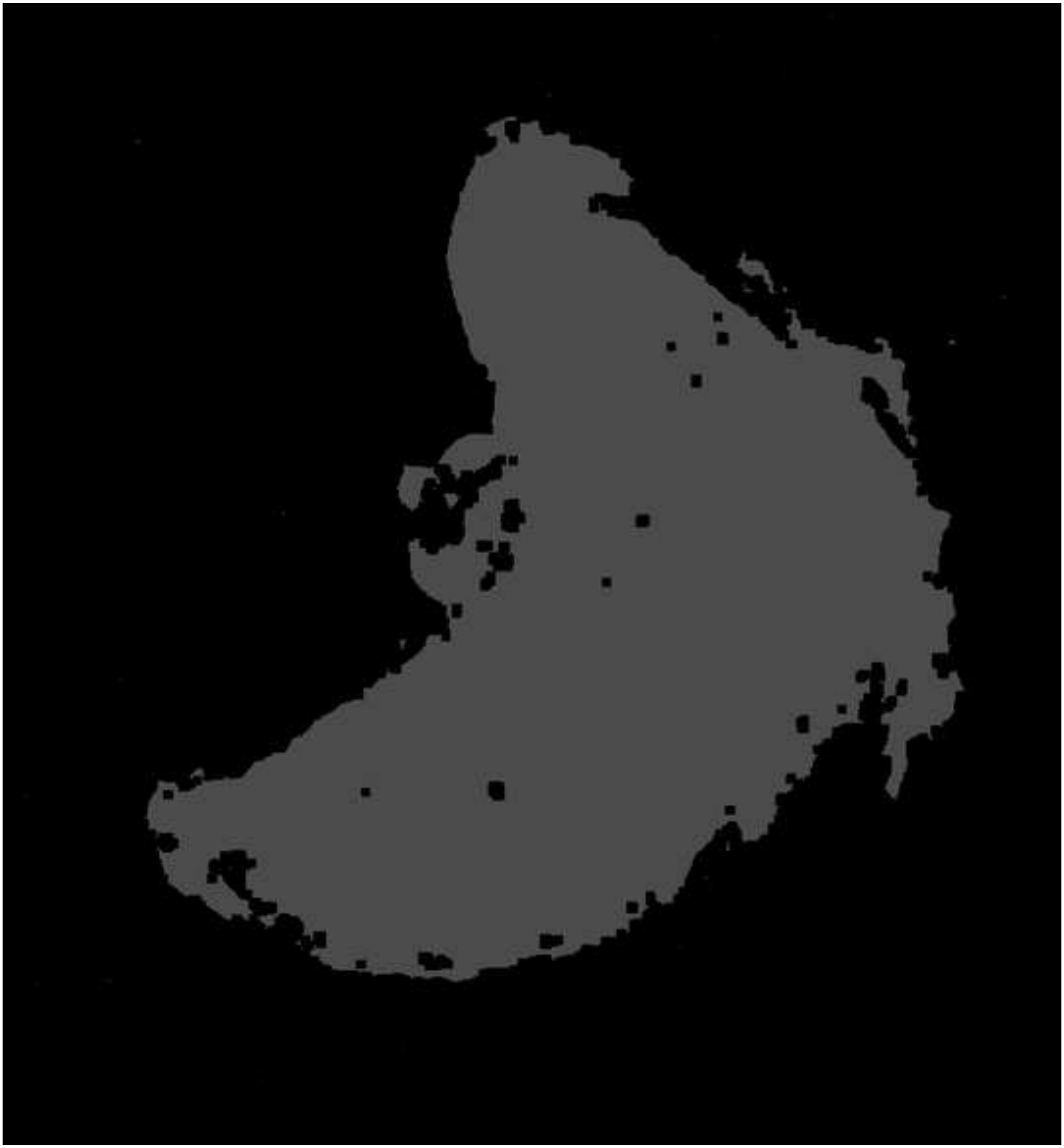

Click here to access/download;Figure;IC-CX-00027-01_thumbnail.png

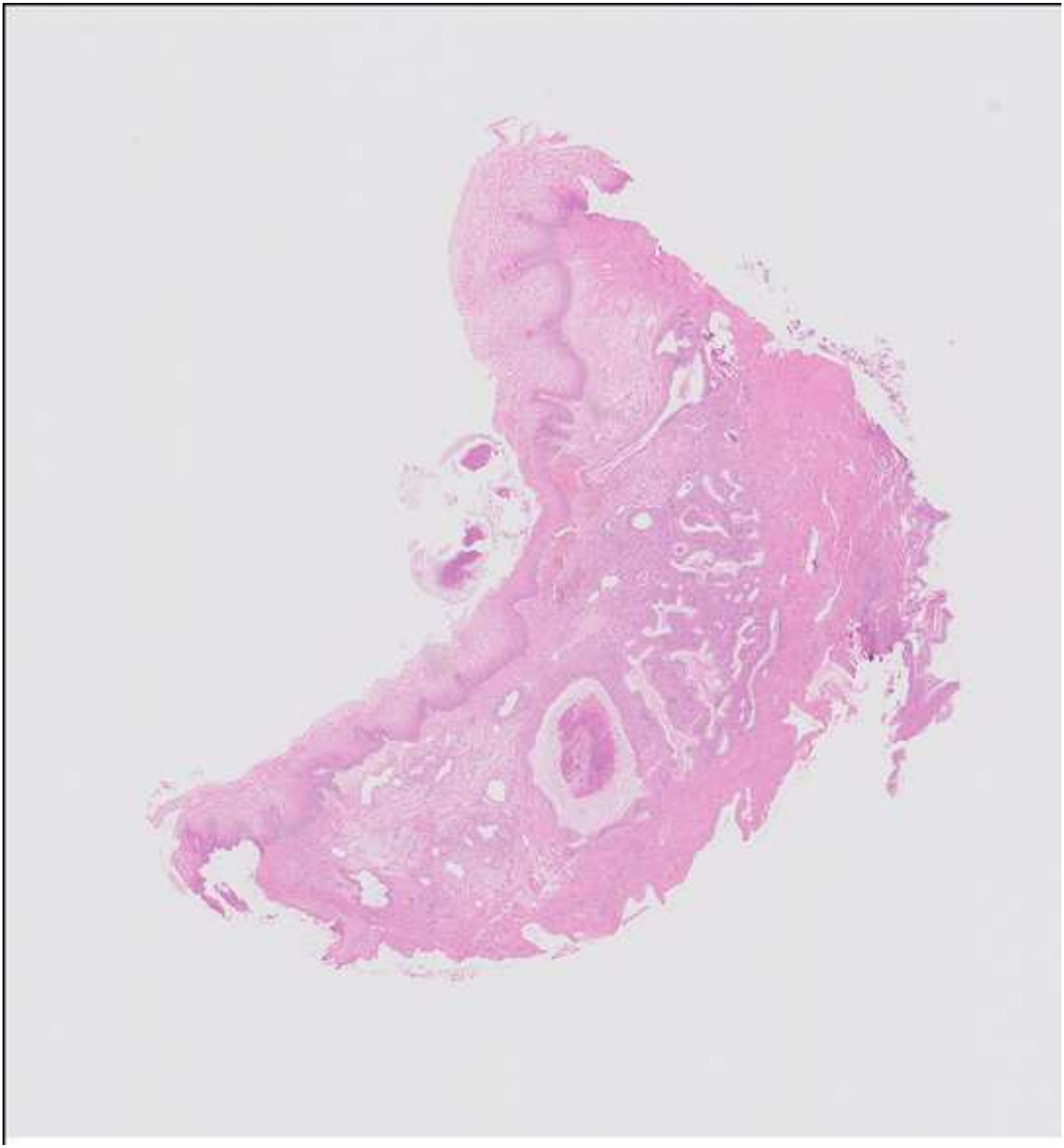

Click here to access/download;Figure;IC-CX-00029-01_mask.png

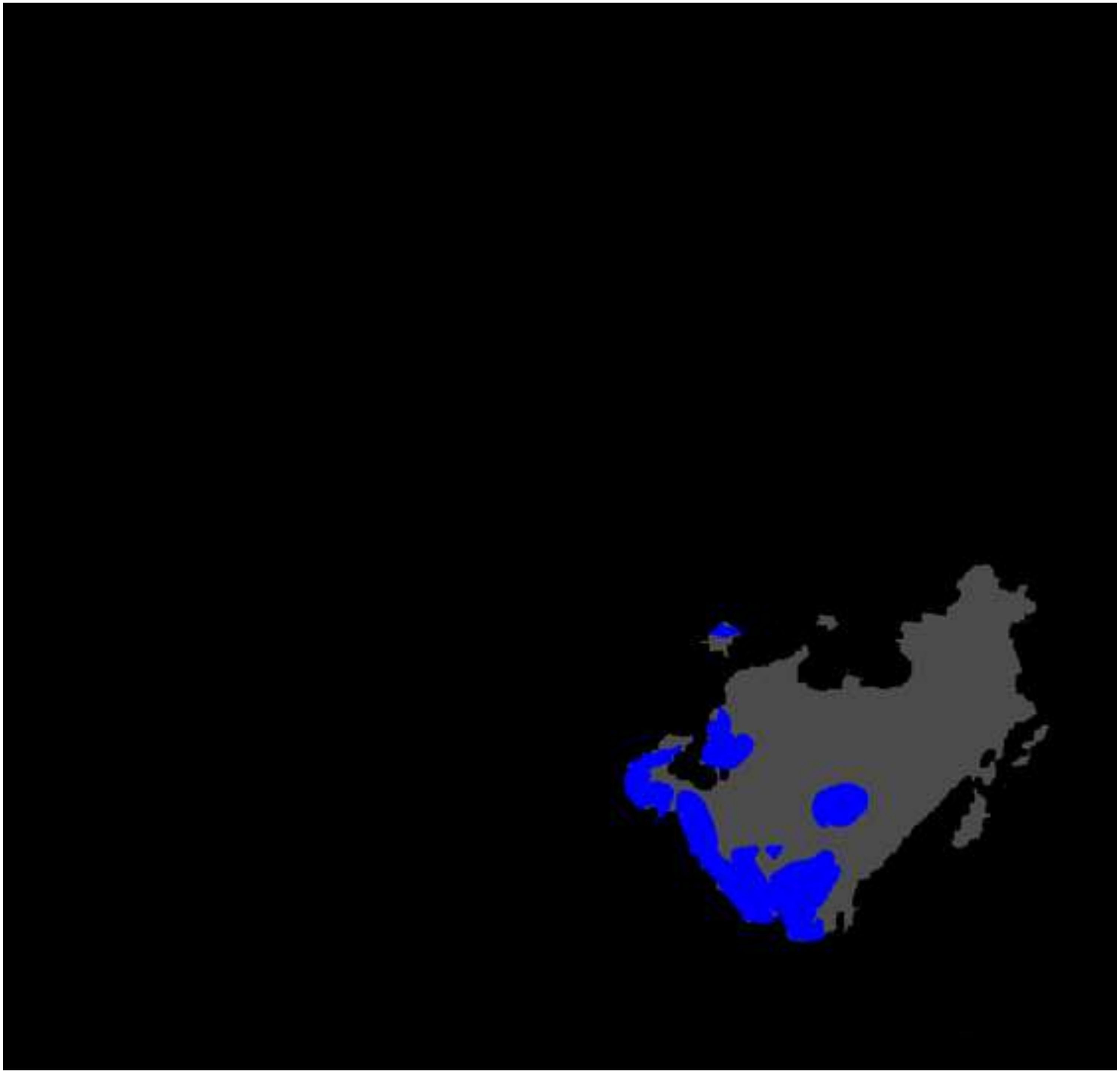

Click here to access/download;Figure;IC-CX-00029-01_thumbnail.png

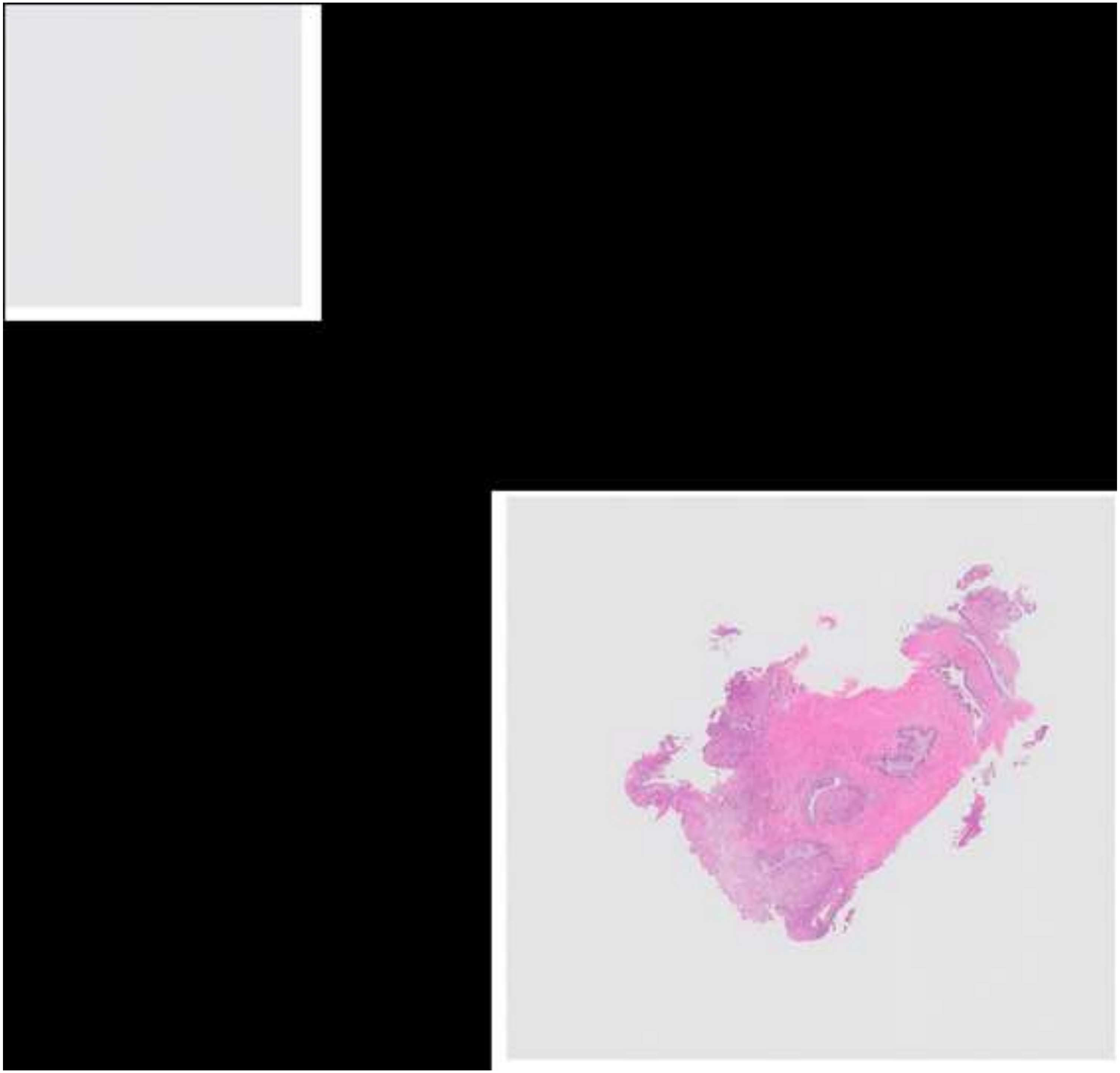

Click here to access/download;Figure;IC-CX-00049-01_mask.png

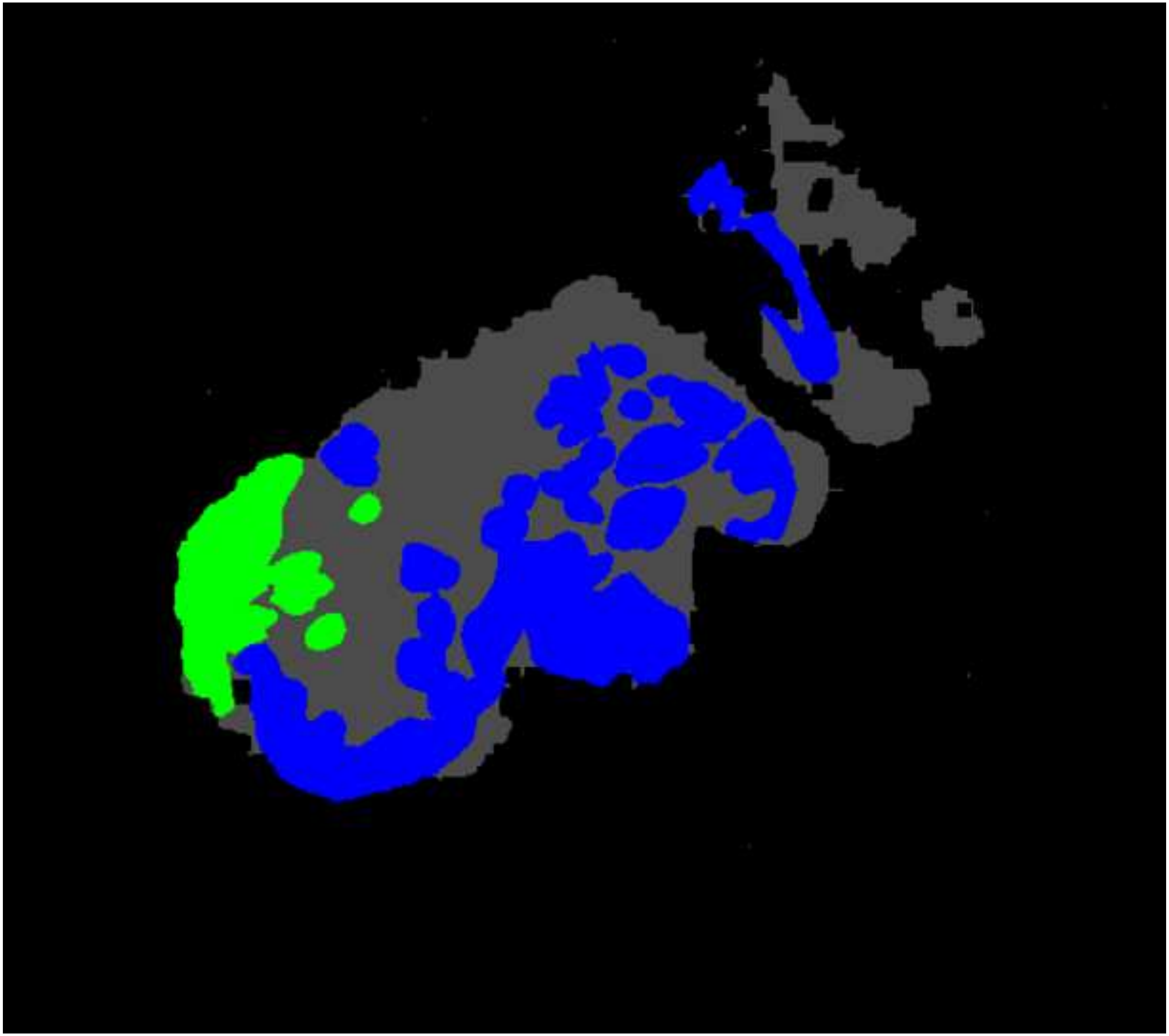

Click here to access/download;Figure;IC-CX-00049-01_thumbnail.png

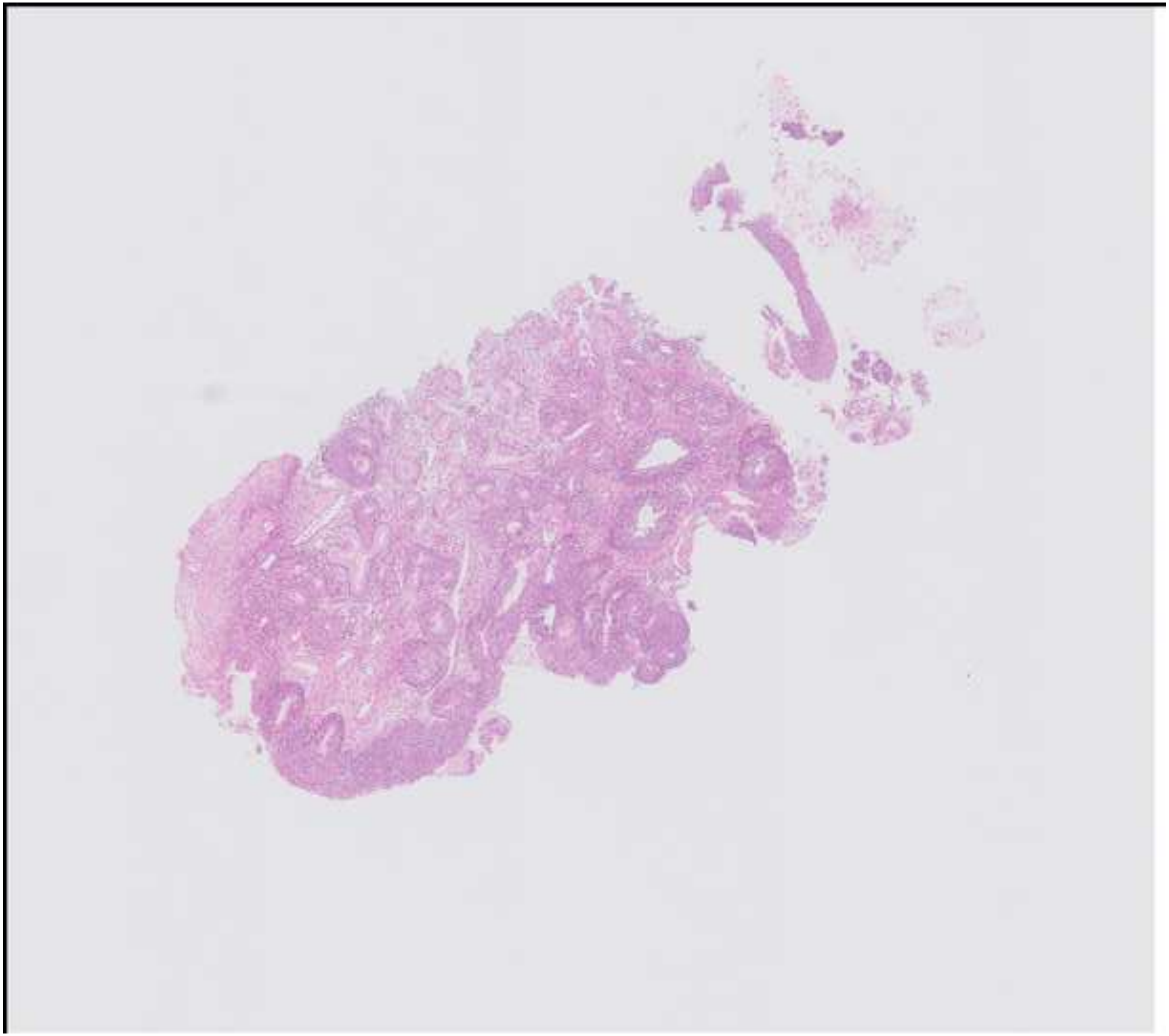

Click here to access/download;Figure;IC-CX-00053-01_mask.png

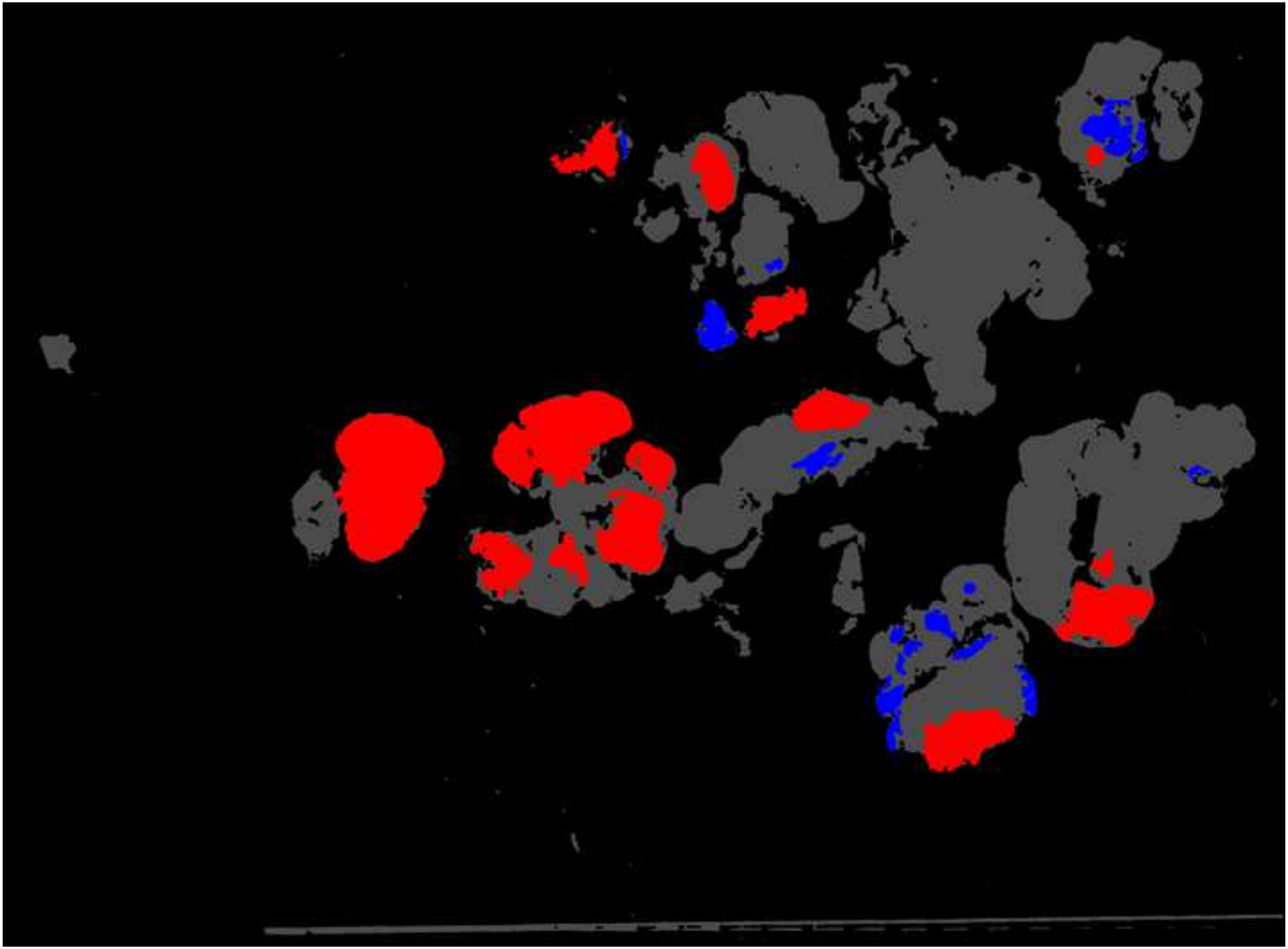

Click here to access/download;Figure;IC-CX-00053-01_thumbnail.png

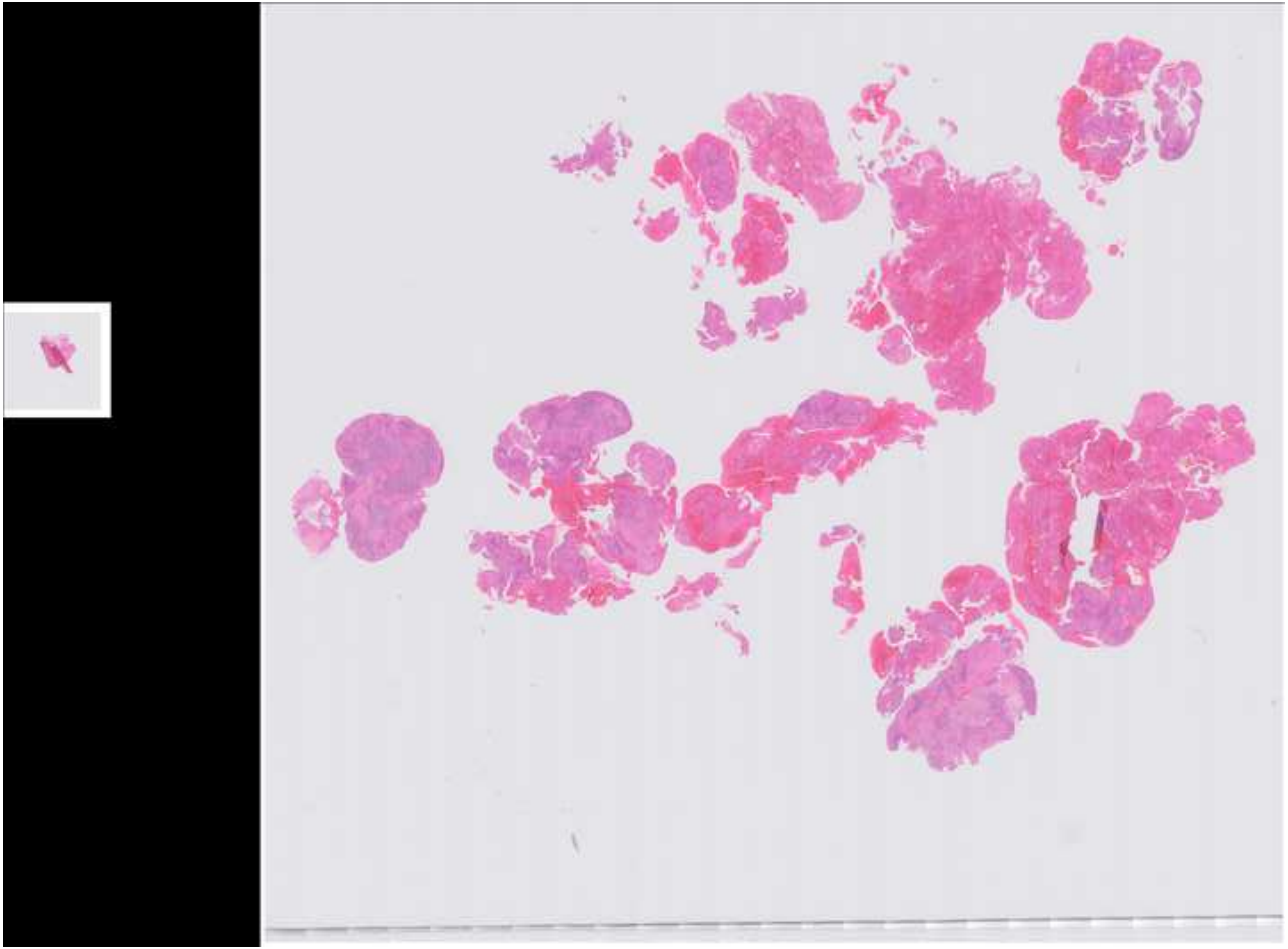

Click here to access/download;Figure;IC-CX-00129-01_mask.png

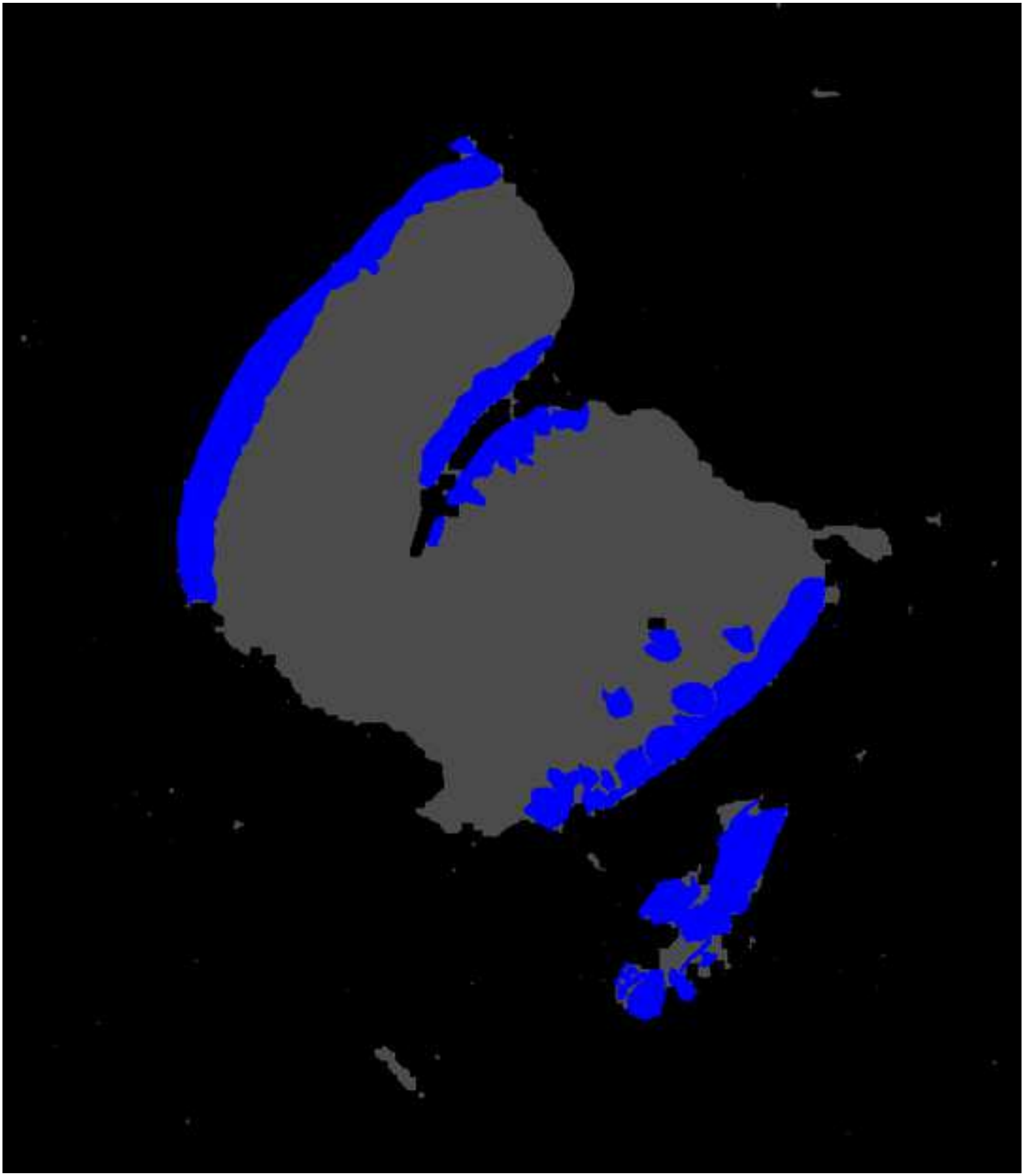

Click here to access/download;Figure;IC-CX-00129-01_thumbnail.png

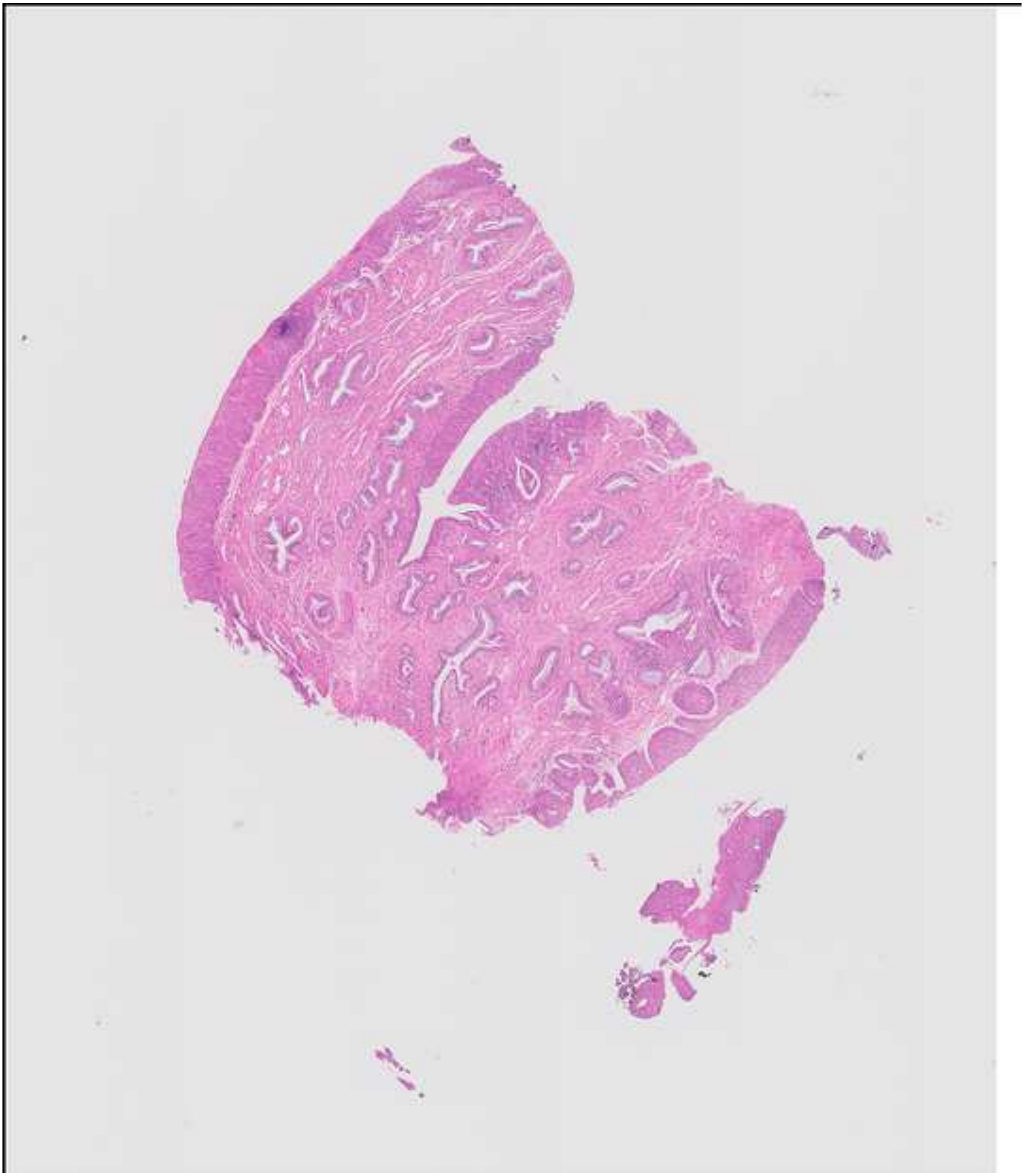

Click here to access/download;Figure;malignant_heatmap.png

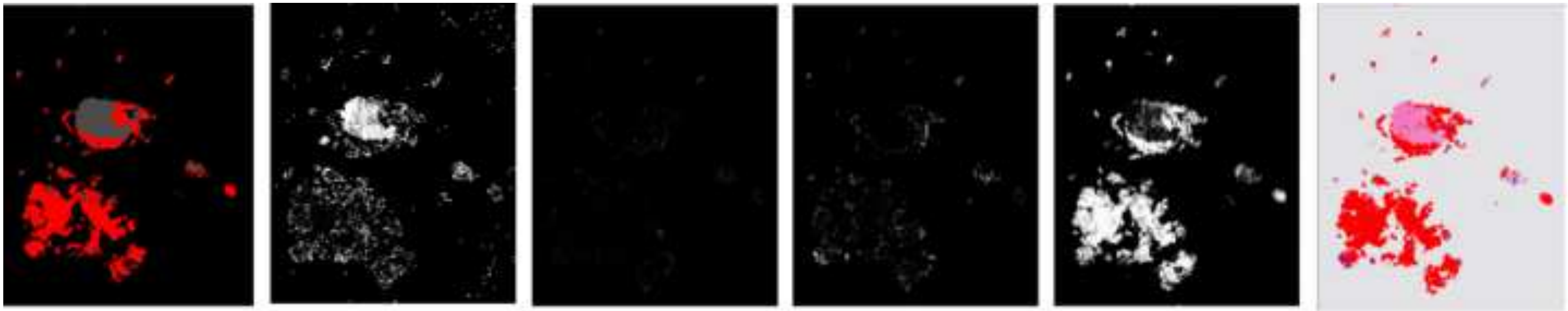

(a) (b) (c) (d) (e) (f)

Click here to access/download;Figure;architecture.png

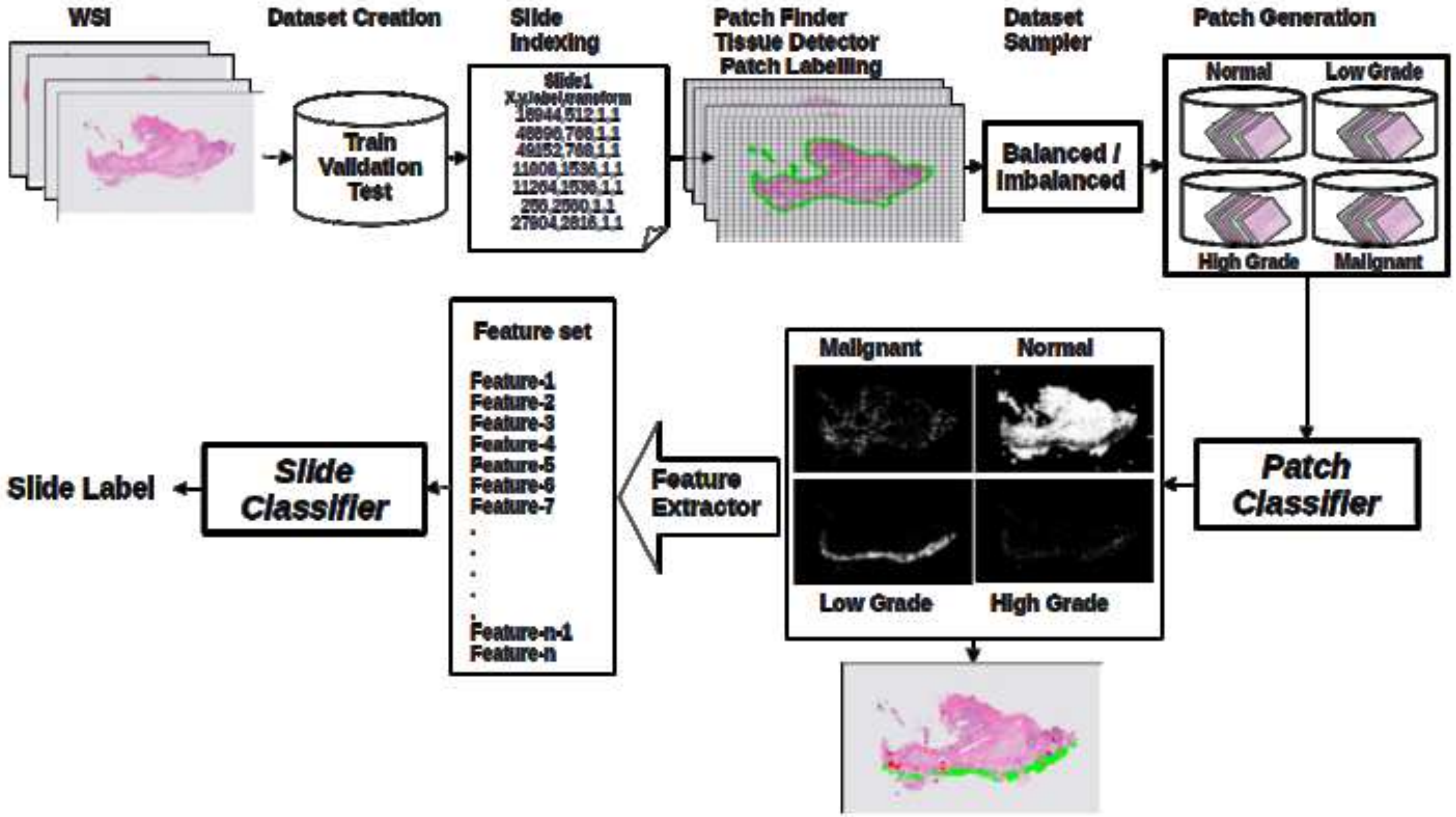

Click here to access/download;Figure;Figure_normal_LG_HG_malignant_HE

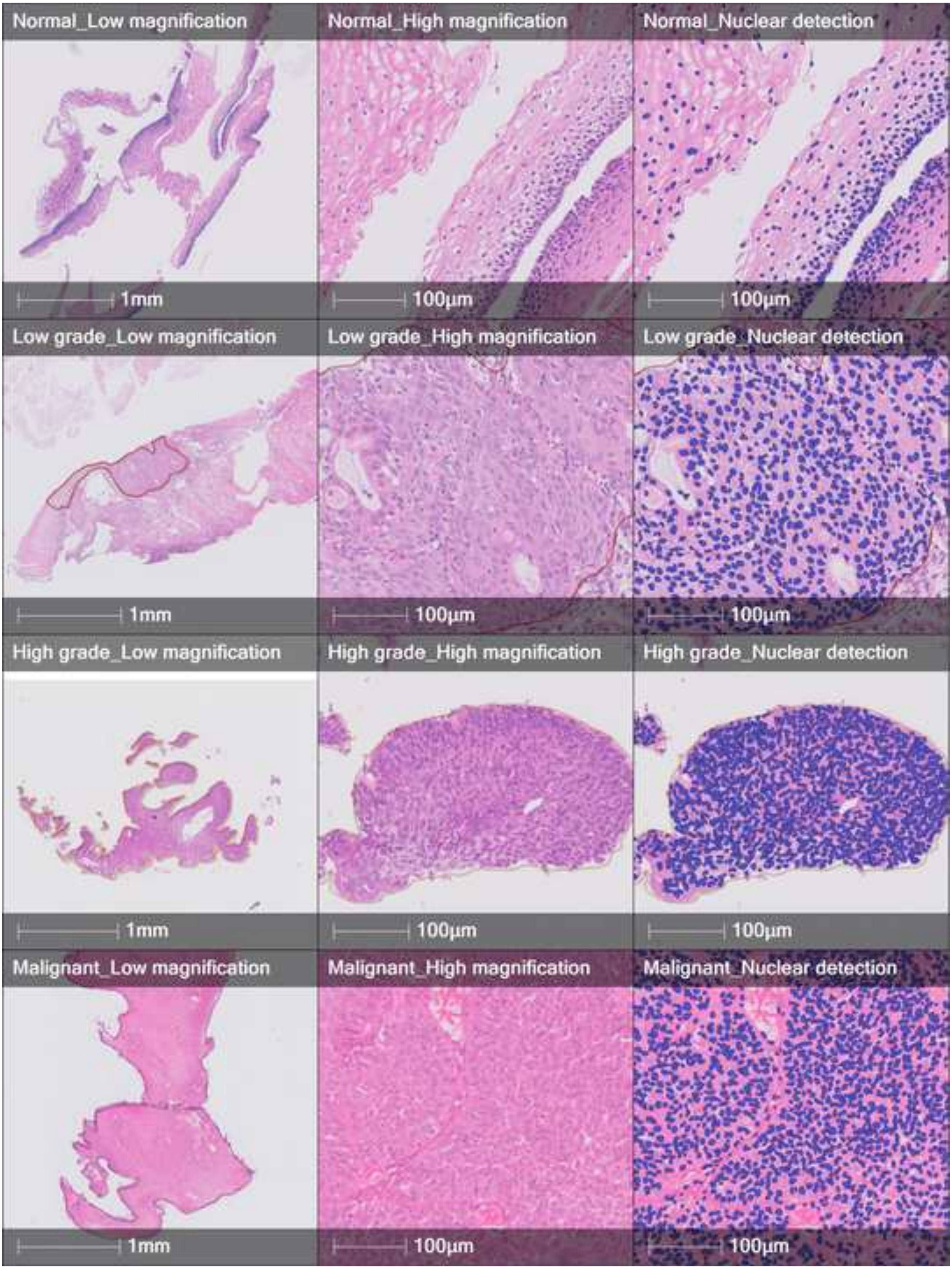

Click here to access/download;Figure;IC-CX-00012-01_mask.png

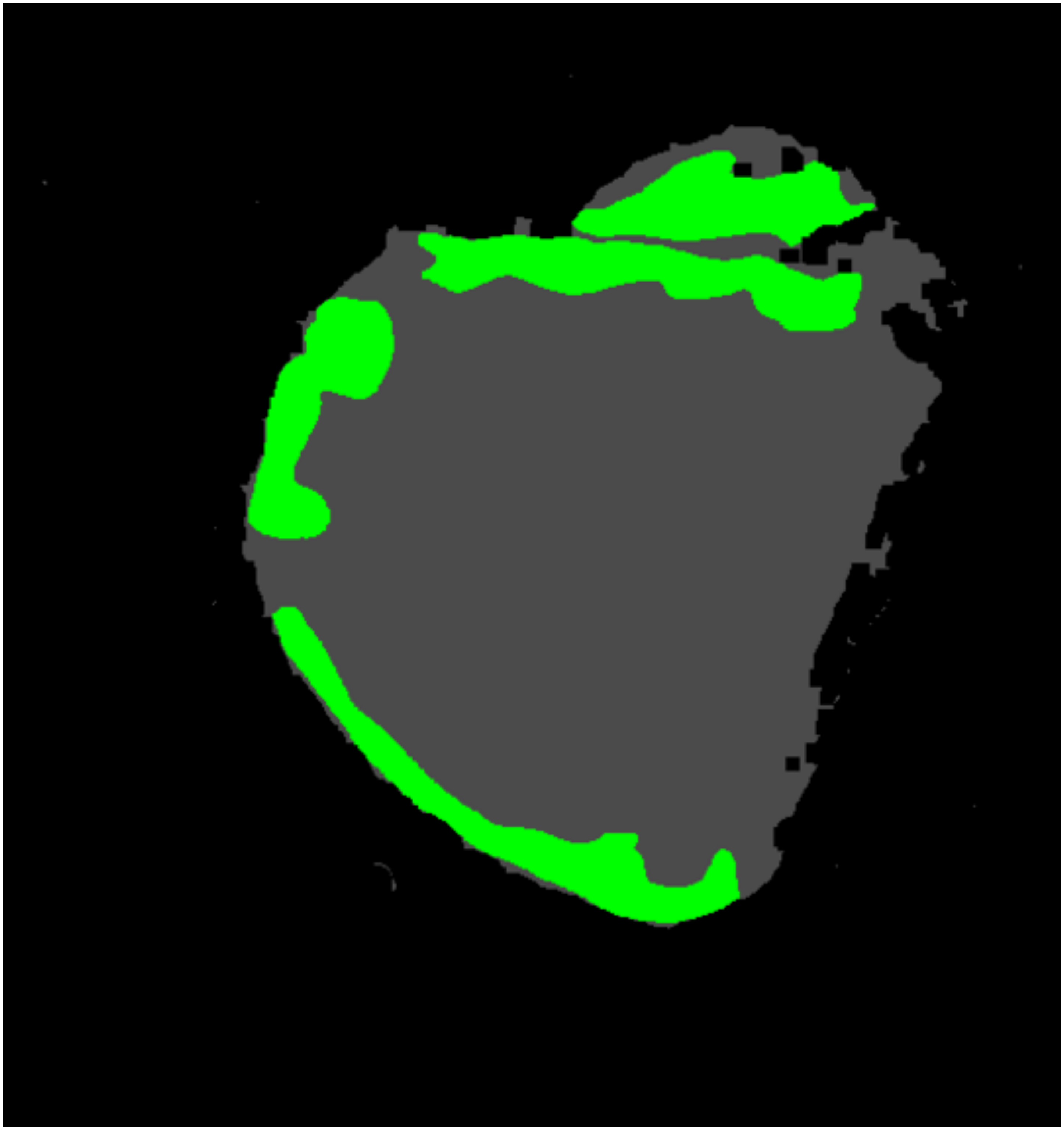

Click here to access/download;Figure;IC-CX-00012-01_thumbnail.png

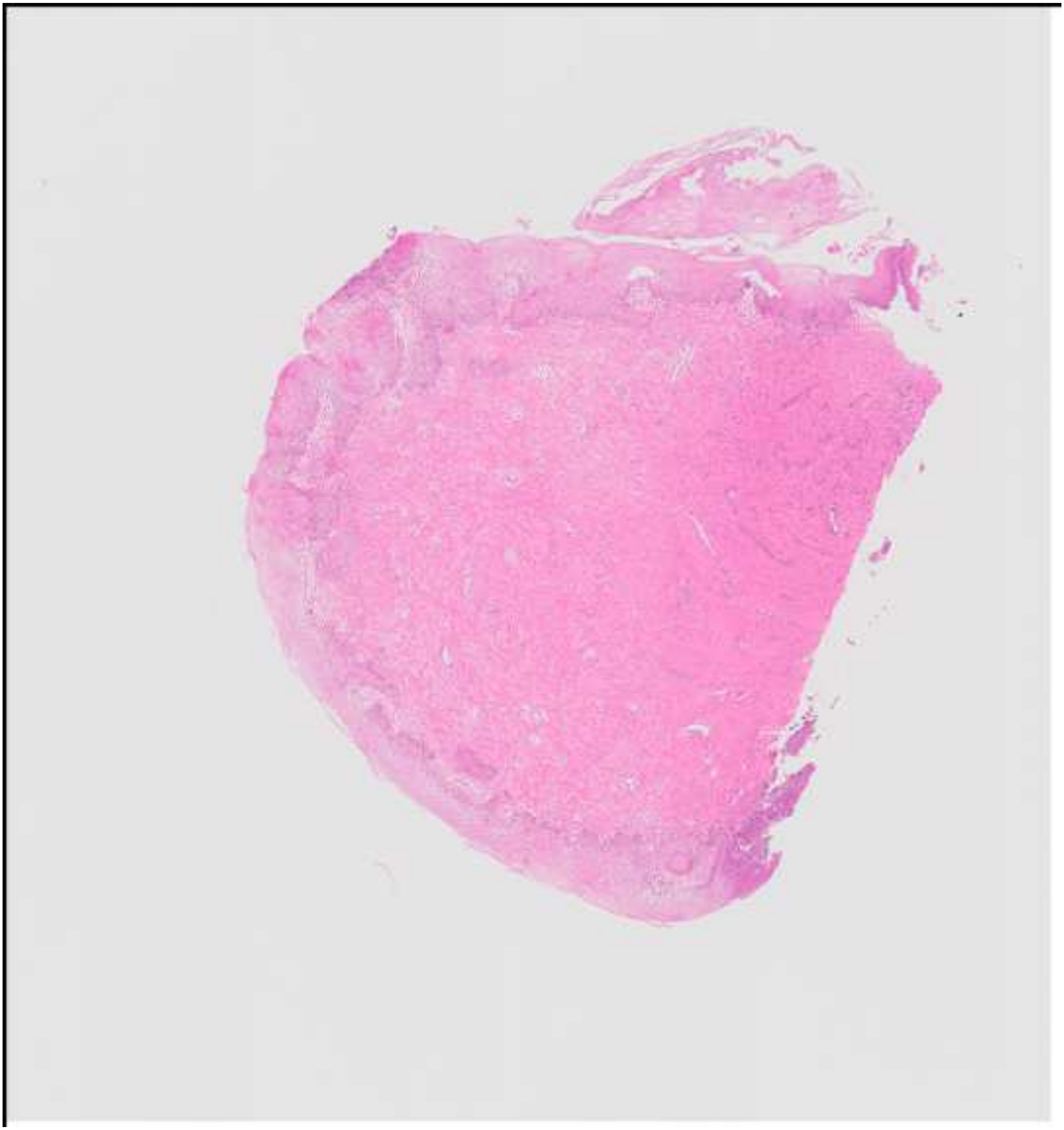

Click here to access/download;Figure;IC-CX-00020-01_mask.png

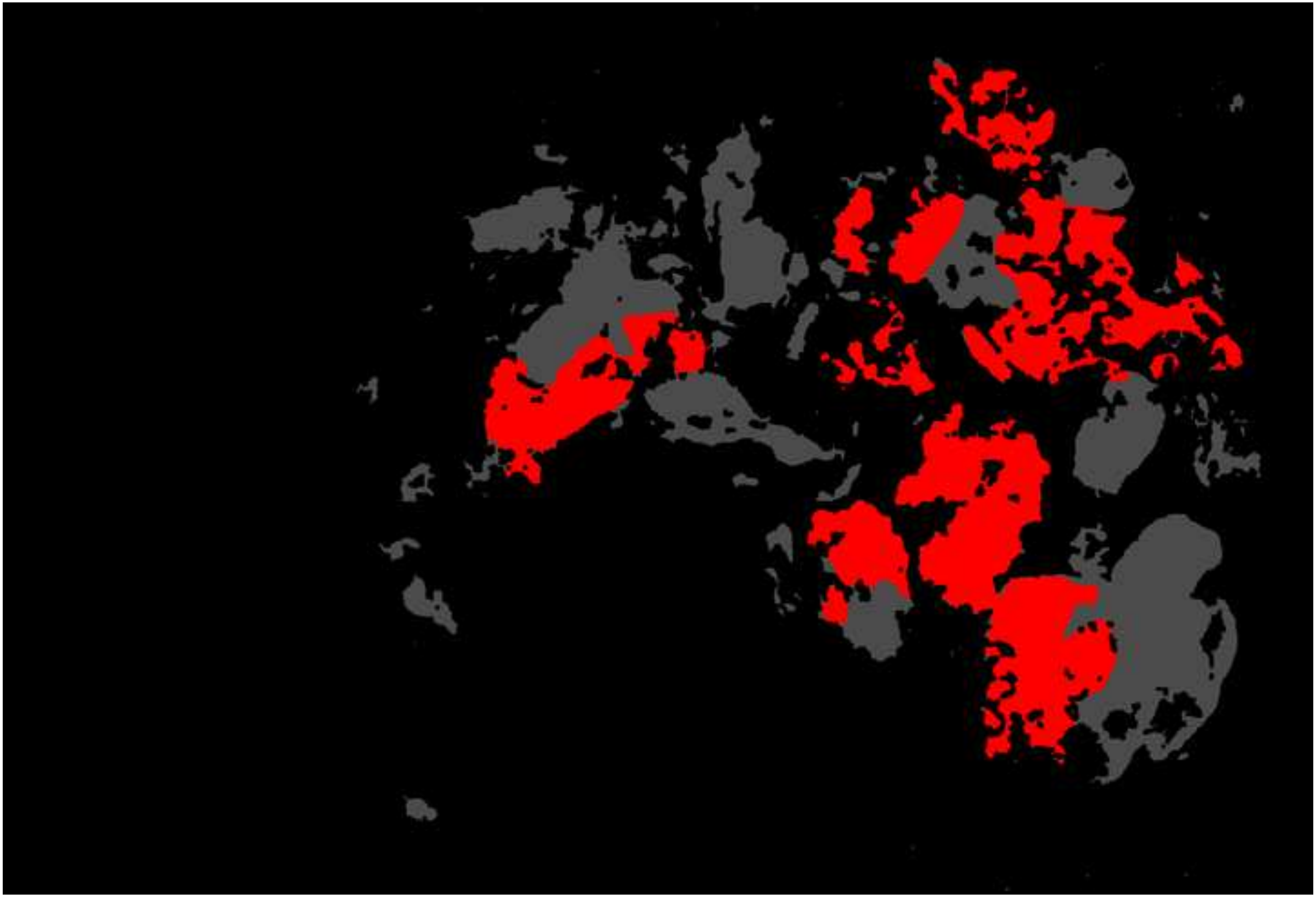

Click here to access/download;Figure;IC-CX-00020-01_thumbnail.png

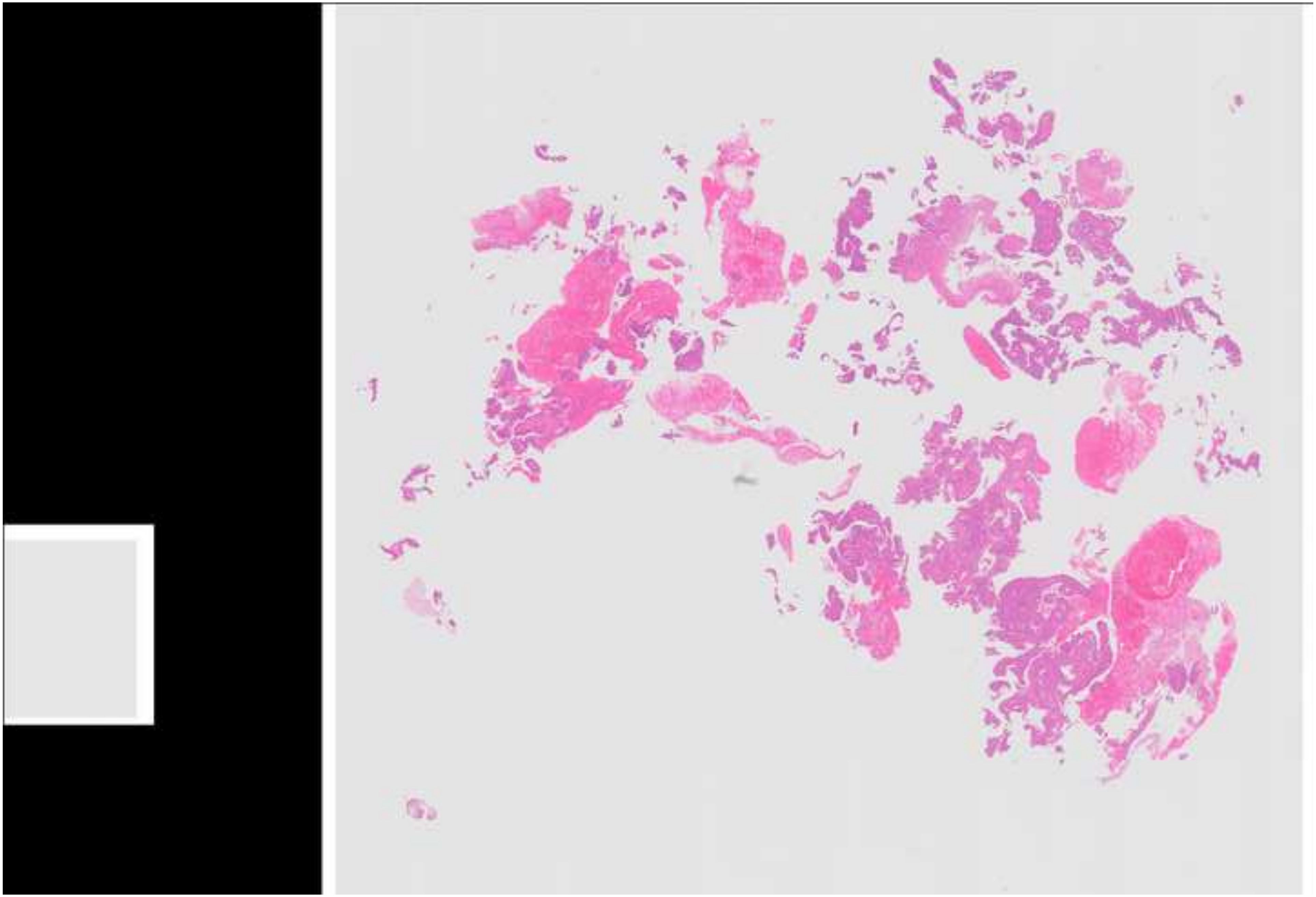

Personal Cover

Click here to access/download;Personal Cover;Final- Giga-cervical reviewers comments-07Feb25-final.docx

Editor GigaScience

School of Medicine
University of St Andrews
North Haugh
St Andrews UK
KY16 9TF

14 February 2025

Dear Editor,

We Thank you for giving us the opportunity to respond to reviewers' comments on our manuscript entitled: **"Cervical Whole Slide Images Dataset for Multi-class Classification (ID: GIGA-D-24-00162)".**
Please find the attached our detailed point-by-point response to the reviewers' comments, together with a revised version of our manuscript. In order to address some issues, we have added James D Blackwood as an author.
We hope that our revisions sufficiently address all concerns and improve the quality of our manuscript. We are grateful for the opportunity to revise and resubmit our work and look forward to your further feedback.

Yours faithfully,

In Hwa Um

Reviewer #2:

1. ***This work is pretty well-structured. Please consider summarizing repetitive information to enhance readability. The abstract is informative but can be more succinct. Focus on the core contributions and potential impacts in fewer words.***

   We have amended the abstract to be more succinct as requested.

2. ***The annotation process is crucial for the dataset's quality. However, the paper lacks detailed information on the inter-observer variability and consensus-building among pathologists. Including metrics on annotation agreement and methods to resolve discrepancies would strengthen the dataset's reliability.***

   We have added a section to explain how we dealt with interobserver variation, based on our original research paper (Mohammadi et al, "Automated reporting of cervical biopsies using artificial intelligence", https://doi.org/10.1371/journal.pdig.0000381) which presented that Cohen's kappa (p.11-12, 3.Inter observer variations) was utilised to calculate the score between three observers in discrepant cases and the higher level of agreement was selected.

3. ***The details on the selection criteria for pathologists, such years of experience and if they are the specialists, in the annotation process and any training they received to ensure consistency.***

   The manuscript summarises relevant details about the annotation team and their qualifications. All histopathologists who annotated biopsies are registered with GMC and Royal College of Pathologists, with subspecialty expertise in gynaecological pathology. Each participating pathologist has a minimum of 5-10 years of post-specialist training experience, with some having over 15 years of expertise in gynaecological pathology. To ensure high-quality and standardised annotations, pathologists reviewed a detailed annotation protocol based on internationally recognised guidelines (WHO classification of tumours, 5th Edition).

4. ***The paper mentions various staining protocols and tissue handling methods and such variations are crucial to build a robust model. Discussion on how these variations can be quantified and/or normalized or accounted for during data preprocessing.***

   The data from different laboratories were combined before selecting the training set, on the basis as we state, that mixing these slides from different sources minimises the chance of over fitting related to technical differences which are not controllable.

5. ***The paper could benefit from a detailed evaluation of the dataset's utility. Including results from preliminary experiments using the dataset to demonstrate its effectiveness in training machine learning models.***

   Preliminary results are present in the primary research paper (Mohammadi et al, “Automated reporting of cervical biopsies using artificial intelligence”, https://doi.org/10.1371/journal.pdig.0000381) in which we reported an AI model to assist in the diagnosis of cervical biopsies. The model was trained on a dataset of whole slide images (WSIs) of cervical biopsies, which were annotated by expert pathologists. The AI systems was designed to identify and classify various cervical lesions, aiming to reduce the workload of pathologists by highlighting areas of interest and potentially decreasing the time required for diagnosis. The study demonstrated that the AI model could effectively distinguish between different types of cervical tissue abnormalities, suggesting its potential utility in clinical settings to support pathologists in the diagnostic process. We believe that reference to this paper should help the user of the dataset to formulate their own plans and experiments.

6. ***Authors can compare this dataset with existing cervical image datasets. Highlight unique aspects and advantages, such as size, diversity, or annotation quality.***

   This is the only large study with images of cervical biopsies as opposed to cytological preparations: there is no comparable dataset available as far as we know. We have brought this point out.

7. ***The authors can include information on the licensing and ethical considerations for using the dataset in research and clinical applications. If this dataset can be used for commercialization purpose? If there is any legal liability of using the given dataset to build the diagnostic models.***

   As we described in the section “Data Availability”, this dataset can be freely accessed for any purpose without requirement of permission from the authors. It is ethically approved for release and is completely anonymised. As regards the question of legal liability, this is a generic issue, but it is most unlikely that a commercial developer could or would rely on a single dataset upon which to base an application for regulatory approval.

8. ***Authors can outline and discussion potential future directions for expanding the dataset or improving its annotations.***

   We are unclear what additional discussion would be valuable. For any clinically useful tool to be developed and implemented, further equivalent datasets would need to be made available.

9. ***The paper does not address the potential for inter-observer variability in annotations and quality control. This is a critical aspect that can affect the dataset's reliability and should be discussed in detail, while this does not impair the current value of the dataset.***

   Please see the answers of Q2 and Q3. We believe we have addressed these issues and that pathologists advising any future projects would be very aware of the subjective nature of pathological classification.

10. ***The technical processes, such as the conversion of image formats and the specifics of machine learning algorithms used, are not sufficiently detailed. Readers would benefit from a deeper understanding of these processes.***

    The process is described in Mohammadi et al, "Automated reporting of cervical biopsies using artificial intelligence", https://doi.org/10.1371/journal.pdig.0000381, including how to convert image formats and the specifics of machine learning algorithms. Our experience in discussion with investigators requesting access to data is that they wish to develop their own methodologies depending on the purpose for which they require the data.

11. ***Understand this is mainly a database paper, but the lack of preliminary results demonstrating the dataset's utility in machine learning applications is a significant omission. Including such results would provide concrete evidence of the dataset's value. If the author has a research paper published, in preparation or archived, can indicate in the manuscripts, which will not affect the sharing this database through a paper in the community.***

    We already have included Reference 5, which describes the original research which we believe provides a useful framework for other investigators to use this dataset. In our preliminary discussions and sharing drafts with Dr Chris Armit and colleagues, we understood that providing the largest dataset of cervical biopsies without any restrictions as to use would be seen as sufficient value.

12. ***A comprehensive guideline for researchers on how to use the dataset effectively is very much helpful to increase the impact of this database, including best practices for preprocessing and annotation usage.***

    In discussions with colleagues, it is clear there is no uniform approach to using data. We believe this is out of scope for a data note and would require consensus across pathology as a discipline. We hope that by providing completely free access to the data that a community will use the information thus inform and refine guidelines and best use principles. That is our primary reason for making these data available.

13. ***Please ensure that the dataset and any accompanying code are accessible through public repositories. Provide clear instructions for accessing and using the dataset.***

    This has been done and referenced, following guidance from Dr Chris Armit when he was working with GigaScience.

Reviewer #3:

14. ***The slides are provided in the iSyntax format, which is not compatible with the OpenSlide library - the most commonly used in the ML community. Offering the slides in a more universal format, such as TIFF, could greatly enhance the accessibility and usability of the dataset for a broader audience.***

    Glencoe software was used to convert iSyntax format to ome-tiff. https://www.glencoesoftware.com/blog/2019/12/09/converting-whole-slide-images-to-OME-TIFF.html as described in the text.
    In preliminary discussions with Dr Armit, we agreed that although iSYNTAX can be an irritation, it is possible to convert images, and some investigators want access to Philips-originated images precisely to resolve its relative difficulty of accessibility.

15. ***The proposed dataset-split deliberately places WSIs from the same lab (or at least the same preparation protocol) in both the training and testing sets. I would be interested in understanding the rationale behind this choice, as it could potentially lead to inflated test accuracy.***

    Please see the answer of reviewer2 -Q4. We believe the mixing actually reduces the chance of bias and overfitting. In addition, a key principle of the ethical approval granted for completely open access, freely available data was its complete de-identifying of images, linking it to individual pathologists or sites. The alternative was a much smaller database being made available or to restrict access. As there is no equivalent set of cases, we felt it was more important to make as large a cohort as possible freely available.

16. ***The writing in the manuscript could be improved for clarity. At times, the current phrasing makes it challenging to understand the authors' intentions. Below, I've outlined some paragraphs where clarity could be improved:***

    "Context" Section:
    The main point of this paragraph is somewhat unclear. It presents mostly WSI analysis algorithms (presenting in detail two of those works - maybe you could detail why those works are important in this context?). However, because this paper is presenting a new dataset, I would find very useful to present here the other available public datasets of cervical biopsies as well as their sizes and characteristics, for instance.

    Another dataset - "Robust whole slide image analysis for cervical cancer screening using deep learning", Nature communications, 2021 Sep 24; 12(1):1–0, https://doi.org/10.1038/s41467-021-25296-x was described in Mohammadi et al, "Automated reporting of cervical biopsies using artificial intelligence", https://doi.org/10.1371/journal.pdig.0000381.

17. ***"Data Split to Train and Test Sets" Section:
The sentence, "During the annotation process, these labels were double-checked, and in approximately 5% of the cases, the final label associated with the scanned slide was different," could be clarified. In particular, it is unclear what the initial labels.***

    We have clarified the text

18. ***The phrase, "The corrected labels post-annotation were the labels used for training and testing," would benefit from further clarification. Does this imply that the ML algorithm (« training and testing.. ») was trained before the slides were fully annotated?
The reference to "[…] described above" is somewhat vague. To which percentages are you referring to ?***

    We have clarified this section

19. ***"Annotation Process" Section:
I understand from this section that each slide is assigned both a main label and a sub-label. If pixel-level annotation masks are also available, it would be useful to provide additional details on this process also. The statement, "Primary annotation was performed either by one of the four pathologists or by a biomedical scientist specifically trained for this project," seems to conflict with the earlier mention that each slide is randomly assigned to one of the four pathologists. Additionally, it would be helpful to clarify whether the "primary annotation" is the same as the "main label" mentioned later. If so, further information on who performed the sub-category annotations would be valuable.***

    We have attempted to clarify this. First annotations were undertaken by expert biomedical scientists. Then they were allocated randomly to the pathologists to review the annotations. If there was no consensus a third review was undertaken. Histopathology is by its very essence subjective. By having multiple reviews and consensus we have tried to minimise variation.

20. ***Including the identity of the annotator (encoded as a discrete value) in the metadata could be beneficial, as it might help account for this potential source of bias.***

    Discussion of interobserver variation has been improved. As discussed above, we were encouraged by the ethics committee to release minimum information that might lead to identifiability. We made a judgement call that having a freely available large dataset outweighed more personalised information about reviewers, even though it would have been anonymous. We have attempted to reduce bias by always ensuring there is a consensus diagnosis from at least two observers, rather than relying on a single observer.

21. ***"Data Validation and Quality Control" Section:***
    This paragraph could be clearer, particularly the sentence: "The gold standard was the pathologists' diagnosis and where there was discrepancies, by consensus review. » I did not understand it.

    We have altered wording.

22. ***The relevance of the subsequent paragraphs, "Gynaecological Cancer AI Project" and "Extracting Nuclear Morphological Features Using Indica Halo AI," is not clear to me. In the former, the algorithm is described with insufficient detail for reproduction or comprehension ("The features extracted from the heatmaps generated at the patch level are used for training an ML classifier to form the final slide-level predictions for each slide." What final ML classifier is used? Which features are extracted from the heatmaps?). In the latter, while the idea of extracting nuclear morphology per pathology class is promising, it's unclear why it is described here. Do you provide this extraction? What are the performance metrics for the ML models trained on these features?***

    The specific questions are addressed in reference 5. We have attempted to clarify the text.

23. ***Other Remarks:***
    There are some minor errors in the text (e.g., "the achieves of the Glasgow Royal Infirmary," or nested parentheses errors like "(low grade (including …()") that could benefit from careful proofreading.
    Some figures, such as "architecture.png," are of very low quality and could be improved to better support the manuscript.

    Thank you for drawing our attention to these points
