# Supplementary material for: Cervical whole-slide images dataset for multiclass classification
Source: Gigascience. 2025 Nov 29;14:giaf144. doi: 10.1093/gigascience/giaf144 (PMC12751090; doi:10.1093/gigascience/giaf144)
Supplement: giaf144_GIGA-D-24-00162_Revision_2 [file giaf144_giga-d-24-00162_revision_2.pdf]

GigaScience

Cervical Whole Slide Images Dataset for Multi-class Classification

--Manuscript Draft--

**Manuscript Number:**	GIGA-D-24-00162R2
**Full Title:**	Cervical Whole Slide Images Dataset for Multi-class Classification
**Article Type:**	Data Note
**Funding Information:**	Innovate UK (104690) — Prof. David Harrison
**Abstract:**	The clinical pathway for prevention and treatment of cervical cancer depends on cytology and then the assessment of biopsies, fragments of tissue removed for histological examination. This can be a significant workload and is an obvious exemplar to explore triage based on machine learning analysis of slides. Limited access to large annotated datasets of human diseased tissue is a major obstacle to developing standards and algorithms that can assist diagnosis. We present a dataset comprising 2539 whole slide images of cervical biopsies, each annotated by several pathologists and consensus on diagnosis and individual features agreed.<br>Each whole slide image represents one slide per patient, in iSyntax format with manual annotations by pathologists in Jason format. Each whole slide image is assigned a category label which is the final diagnosis of the image, and a subcategory label which declares in which subcategory the image is found. This dataset has been used to build a model that accurately predicts diagnosis, allowing the possibility of automatically triaging biopsies, so that the most significant pathologies can be identified rapidly and those patients selected for immediate treatment. The level of annotation, at sub-slide level, and the number of cases is unique in public databases and should allow investigators to explore multiple aspects of computer vision relevant to human tissue diagnosis, with no limitation placed on access to the whole slide images.
**Corresponding Author:**	In Hwa Um, Ph.D.<br>St Andrews University<br>St Andrews, Scotland UNITED KINGDOM
**Corresponding Author Secondary Information:**	
**Corresponding Author's Institution:**	St Andrews University
**Corresponding Author's Secondary Institution:**	
**First Author:**	In Hwa Um, Ph.D.
**First Author Secondary Information:**	
**Order of Authors:**	In Hwa Um, Ph.D.
	Mahnaz Mohammadi
	Christina Fell
	David Morrison
	Sarah Bell
	Gareth Bryson
	Sheeba Syed
	Prakash Konanahalli
	Clare Orange
	Prishma Shahi
	David Harrison
	David Harris-Birtill, PhD

Powered by Editorial Manager® and ProduXion Manager® from Aries Systems Corporation

	Ognjen Arandjelovic, PhD
	James D Blackwood
**Order of Authors Secondary Information:**	
**Response to Reviewers:**	Dear Editor,<br><br>We would like to express our sincere thanks for the opportunity to revise and resubmit our manuscript entitled: “Cervical Whole Slide Images Dataset for Multi-class Classification (ID: GIGA-D-24-00162)”. We appreciate the constructive feedback provided by the reviewers and the editorial team.<br>We have carefully considered all comments and have made corresponding revisions to the manuscript. Please find the attached our detailed point-by-point response to the reviewer’s comments, along with a revised version of our manuscript.<br>We hope that our revisions and clarifications we have provided sufficiently address all concerns and further enhance the quality and clarity of our work. We are grateful for the thoughtful suggestions and for the opportunity to improve our manuscript.<br>Thank you again for your time and consideration. We look forward to your feedback.<br><br>Yours faithfully,<br>In Hwa Um<br><br>--<br><br>Reviewer #3:<br><br>14. Slide format:<br>While I appreciate the discussion on this topic, my stance remains unchanged: a significant portion of the pathology and machine learning community relies on OpenSlide-compatible formats. This discrepancy may limit the dataset's impact (I have never used OME-TIFF but from what I can read, it is compatible with QuPath but not OpenSlide. Do you confirm?)<br>To address this, could the authors consider providing instructions or a Docker environment with a script to facilitate format conversion?<br>This issue is, in my opinion, the primary obstacle to the dataset's widespread adoption.<br><br>We appreciate the reviewer’s continued attention to this important issue. We fully agree that the ISYNTAX file format poses limitations for interoperability within the digital pathology and machine learning communities, many of whom rely on OpenSlide-compatible formats. In response to this concern, we have made available a publicly accessible codebase that enables the conversion of ISYNTAX files to the widely adopted OME-TIFF format. This resource is hosted on Zenodo (Reference 8) and is now explicitly referenced in the Data collection section of the Methods.<br>To further enhance accessibility and reproducibility, we have also provided a Docker environment within the same Zenodo repository. This Docker setup includes all necessary dependencies and scripts required for the conversion process, thereby facilitating easy deployment across different computational environments. The Docker container can be accessed via the following DOI:<br>https://doi.org/10.5281/zenodo.7674764<br>We confirm that while OME-TIFF is not directly supported by OpenSlide, it is compatible with widely used open tools such as QuPath, which supports annotation and visualisation functionalities. We hope that these additional resources and clarifications address reviewer’s concerns and help ensure the dataset’s broader usability and adoption.<br><br>15. Why do you believe so? I would appreciate a clear justification for the authors' belief in this regard. Increasing the number of centers in the training set may have this effect - but if some centers effectively correlate with the output variable, then having this same center in the test set will artificially inflate test results - and with the current dataset design, we lack means to know that. Please see : Howard, Frederick M., James Dolezal, Sara Kochanny, Jefree Schulte, Heather Chen, Lara Heij, Dezheng Huo, et al. « The Impact of Site-Specific Digital Histology Signatures on Deep Learning Model Accuracy and Bias ». Nature Communications 12, n 1 (décembre 2021): 4423.<br>https://eur01.safelinks.protection.outlook.com/?url=https%3A%2F%2Fdoi.org%2F10.1

Powered by Editorial Manager® and ProduXion Manager® from Aries Systems Corporation

	038%2Fs41467-021-24698-1&data=05%7C02%7Cdjh20%40st-andrews.ac.uk%7C4e289b4df86f43b8505508dd5da84e77%7Cf85626cb0da849d3aa5864ef678ef01a%7C0%7C0%7C638769699632897702%7CUnknown%7CTWFpbGZsb3d8eyJFbXB0eU1hcGkiOnRydWUsIlYiOiIwLjAuMDAwMCIsIlAiOiJXaW4zMiIsIkFOIjoiTWFpbCIsIldUIjoyfQ%3D%3D%7C0%7C%7C%7C&sdata=%2BBnZBZ%2B67jEc0xKEJAnRb6sDG2sS1%2B0xi%2BXCZfdvIbE%3D&reserved=0<br><br>We appreciate the reviewer’s thoughtful concern and the reference to Howard et al., which provides important evidence of the risks associated with site-specific biases in histopathology datasets. In designing our dataset split, we were mindful of these challenges and adopted a strategy aimed at balancing generalizability with practical constraints around data diversity and ethical considerations.<br>The full dataset comprises whole slide images (WSIs) from eight distinct centres, each using potentially different staining protocols and preparation workflows. To construct the test set, we deliberately excluded all WSIs from two of these centres (Lab 6 and Lab 8), ensuring that approximately 75% of the test set originates from entirely unseen staining protocols. This portion of the test set allows us to meaningfully assess out-of-distribution (OOD) generalization, which we agree is essential for evaluating the robustness of machine learning models.<br>In addition to this, the remaining 25% of the test set consists of a 10% random sample of slides drawn from the six centres that are also represented in the training set. The inclusion of these in-distribution samples was intentional: it enables us to evaluate how well the model performs on data that comes from distributions similar to the training data. Moreover, restricting the test set to only two centres would have risked introducing another form of bias — namely, the potential for the model to perform well simply because it aligns more closely with the specific characteristics of those two sites. By including a smaller proportion of data from additional centres in the test set, we increase its diversity and reduce the risk of overfitting to idiosyncrasies of a limited number of protocols.<br>Finally, we ensured that the dataset was balanced across diagnostic categories, subcategories, and staining protocols across all splits — training, validation, and testing — to further reduce the likelihood of class imbalance or site-specific confounding effects. It is also important to emphasize that the primary purpose of this GigaScience Data Note is to publicly release a high-quality, well-annotated dataset for the research community. Our goal is to facilitate future work by computer scientists and biomedical engineers, enabling them to develop and benchmark novel algorithms for histopathological image analysis. As such, we have prioritized transparency, accessibility, and broad utility, while acknowledging and documenting any limitations inherent to the dataset design.
**Additional Information:**	
**Question**	**Response**
Are you submitting this manuscript to a special series or article collection?	No
**Experimental design and statistics**<br><br>Full details of the experimental design and statistical methods used should be given in the Methods section, as detailed in our Minimum Standards Reporting Checklist. Information essential to interpreting the data presented should be made available in the figure legends.<br><br>Have you included all the information requested in your manuscript?	Yes

Powered by Editorial Manager® and ProduXion Manager® from Aries Systems Corporation

**Resources**<br><br>A description of all resources used, including antibodies, cell lines, animals and software tools, with enough information to allow them to be uniquely identified, should be included in the Methods section. Authors are strongly encouraged to cite Research Resource Identifiers (RRIDs) for antibodies, model organisms and tools, where possible.<br><br>Have you included the information requested as detailed in our Minimum Standards Reporting Checklist?	Yes
**Availability of data and materials**<br><br>All datasets and code on which the conclusions of the paper rely must be either included in your submission or deposited in publicly available repositories (where available and ethically appropriate), referencing such data using a unique identifier in the references and in the “Availability of Data and Materials” section of your manuscript.<br><br>Have you have met the above requirement as detailed in our Minimum Standards Reporting Checklist?	Yes

Click here to access/download;LaTeX - Main Document (TeX file);main-cervical-30May25.tex

```
This is pdfTeX, Version 3.141592653-2.6-1.40.26 (TeX Live 2024)
(preloaded format=pdflatex 2024.8.2)  30 MAY 2025 04:40
entering extended mode
 restricted \write18 enabled.
 %&-line parsing enabled.
**main-cervical-30may25.tex
(./main-cervical-30May25.tex
LaTeX2e <2024-06-01> patch level 2
L3 programming layer <2024-05-27>

! LaTeX Error: File `oup-contemporary.cls' not found.

Type X to quit or <RETURN> to proceed,
or enter new name. (Default extension: cls)

Enter file name:
! Emergency stop.
<read *>

l.11 ^^M

*** (cannot \read from terminal in nonstop modes)


Here is how much of TeX's memory you used:
 20 strings out of 473583
 533 string characters out of 5732343
 1925908 words of memory out of 5000000
 23012 multiletter control sequences out of 15000+600000
 558069 words of font info for 36 fonts, out of 8000000 for 9000
 1141 hyphenation exceptions out of 8191
 19i,0n,29p,112b,17s stack positions out of
10000i,1000n,20000p,200000b,200000s
!  ==> Fatal error occurred, no output PDF file produced!
```

Manuscript

Click here to access/download;Manuscript;Cervical_Data_Note_GigaScienc

GigaScience, 2023, 1–7

doi: xx.xxxx/xxxx
Manuscript in Preparation
Data Note

DATA NOTE

Cervical Whole Slide Images Dataset for Multi-class Classification

Mahnaz Mohammadi[1,*,§], Christina Fell[1,*], David Morrison[1,*], Sarah Bell[2,†], Gareth Bryson[2,†], Sheeba Syed[2,†], Prakash Konanahalli[2,†], David Harris-Birtill[4,*], Ognjen Arandjelovic[4,*], Clare Orange[1,2,*], Prishma Shahi[1,*], In Hwa Um[1,3,*,§], James D Blackwood[1,*] and David J Harrison[1,3,*]

[1]School of Medicine, University of St Andrews, North Haugh, KY16 9TF, United Kingdom and [2]Department of Pathology, Queen Elizabeth University Hospital, Govan Road, G51 4TF, Glasgow, United Kingdom and [3]Pathology, Division of Laboratory Medicine, Royal Infirmary of Edinburgh, Old Dalkeith Road, EH16 4SA, United Kingdom and [4]School of Computer Science, University of St Andrews, North Haugh, KY16 9SX, United Kingdom

[*]mm459, cmf21, dm236, dcchb, oa7, celo1, ps289, ihu, jdb20, david.harrison@st-andrews.ac.uk
[†]Sarah.Bell, Gareth.Bryson, sheeba.syed, prakash.konanahalli@ggc.scot.nhs.uk
[§]Corresponding author: mahnaz.mohammadi0@gmail.com, ihu@st-andrews.ac.uk

Abstract

The clinical pathway for prevention and treatment of cervical cancer depends on cytology and then the assessment of biopsies, fragments of tissue removed for histological examination. This can be a significant workload and is an obvious exemplar to explore triage based on machine learning analysis of slides. Limited access to large annotated datasets of human diseased tissue is a major obstacle to developing standards and algorithms that can assist diagnosis. We present a dataset comprising 2539 whole slide images of cervical biopsies, each annotated by several pathologists and consensus on diagnosis and individual features agreed. Each whole slide image represents one slide per patient, in iSyntax format with manual annotations by pathologists in Jason format. Each whole slide image is assigned a category label which is the final diagnosis of the image, and a subcategory label which declares in which subcategory the image is found. This dataset has been used to build a model that accurately predicts diagnosis, allowing the possibility of automatically triaging biopsies, so that the most significant pathologies can be identified rapidly and those patients selected for immediate treatment. The level of annotation, at sub-slide level, and the number of cases is unique in public databases and should allow investigators to explore multiple aspects of computer vision relevant to human tissue diagnosis, with no limitation placed on access to the whole slide images.

Key words: Whole slide imaging; histopathology; cervix; cervical cancer; digital image database; machine learning; deep learning; healthcare dataset.

Data Description

This large collection of cervical Whole Slide Image (WSI) data serves as a vital resource to improve cervical cancer detection, diagnosis, and research, ultimately contributing to the overall goal of reducing the burden of this preventable and treatable disease on a global scale. Cervical WSI data provides detailed, high-resolution views of cervical tissue samples, enabling healthcare providers to detect abnormalities and precancerous lesions with greater precision. This improved accuracy can lead to earlier intervention and improved patient outcomes. Researchers and data scientists utilize this comprehensive dataset to develop and fine-tune Machine Learning (ML) algorithms and Artificial Intelligence (AI) tools. These tools can automate and streamline the screening process, making it more

Compiled on: May 30, 2025.
Draft manuscript prepared by the author.

accessible and cost-effective, particularly in regions with limited healthcare resources.

The dataset consists of a total of 2539 Haematoxylin and Eosin (H&E) cervical WSIs, with one slide per patient, in iSyntax format requiring 731 Giga Bytes (GB) storage. This dataset was originally created for Gynaecological Cancer AI project which is part of Industrial Centre for Artificial Intelligence Research in Digital Diagnostics (iCAIRD) [1] and was used to develope and train AI for diagnosis of cervical biopsies as either benign dysplastic (pre-cancerous), or neoplastic (cancer). For abnormal biopsies, classification was as: invasive squamous or adeno-carcinoma, intraepithelial neoplasia (low grade (including HP and CIN 1 and high grade (including CIN 2 and CIN 3).

Context

As the demand for AI tools for diagnosis continues to grow, so does the need for high-quality datasets. Datasets are a critical component of AI development as they provide the training data that enables the ML models to learn patterns, relationships, and make prediction. Datasets can be used for training, evaluation and testing different ML models. They also can serve as benchmarks for comparing different algorithms and models. Datasets contribute to the advancement of ML research by providing a foundation for exploring new algorithms, techniques, and models. Hence, quality, diversity, representativeness, size, balance, and potential biases of the dataset are factors that can significantly impact the performance and generalization of ML applications.

Digital pathology offers several benefits and addresses various challenges in traditional pathology practice, making it a valuable and increasingly essential component of modern healthcare. AI has become increasingly important in the field of digital pathology due to its potential to revolutionize the way medical professionals analyse and interpret pathology slides. AI can help identify patterns that may be difficult to spot with the human eye, leading to faster and more accurate diagnoses. It can aid in the early detection of diseases like cancer, by analysing subtle changes in images over time which can lead to earlier interventions and improved patient outcomes.

Histopathology is the microscopic examination of tissue samples to diagnose diseases and understand their underlying causes. Histopathology WSIs are indispensable resources in the field of medical image analysis and ML applications. Staining techniques are essential in histopathology to enhance the visualization of cellular structures and specific components within tissues. Different staining techniques are used to highlight various tissue elements and help pathologists differentiate between normal and abnormal structures. The digital representations of tissue samples stained with Hematoxylin and Eosin (H&E) provide a comprehensive view of cellular and tissue structures at a microscopic level. The color contrast given to different cell types and tissue components, aids pathologists in diagnosing and characterizing diseases and provides rich source of visual and contextual information, making them an ideal input for various ML tasks.

ML algorithms trained on a large dataset of annotated H&E WSIs, can be useful in diagnostic assistance, predicting disease progression, drug discovery and development, educational tools and many more. Trained ML algorithms on WSIs, can assist pathologists in diagnosing diseases, identify patterns and anomalies in tissue samples and consequently lead to more accurate and efficient diagnoses. Detecting and segmenting tumor regions within H&E WSIs is another example which aids in quantifying tumor size, density, and distribution, which are essential factors in disease prognosis and treatment planning. Meaningful features extracted from H&E WSIs, such as texture, shape, and color information can be used to characterize tissue structures, helping researchers and clinicians understand tissue composition and potentially uncover new insights.

A comprehensive survey of cervical histopathology image analysis using machine vision approaches is presented [2]. This paper reviews all the related works of cervical histopathology image analysis using machine vision techniques from 1988 to 2020. In this survey, more than 60 related works are summarized from 1988 to 2019. Authors of [3] propose a GBUL approach to describe the topological information of different tissues in the histopathological images and two stages of unsupervised leaning processes are applied to group the tissues into relevant types. A weakly supervised survival convolutional neural network approach equipped with a visual attention mechanism for predicting overall survival is presented in [4]. The inclusion of visual attention provides insights into regions of the tumor microenvironment with the pathological interpretation which may improve understanding of the disease pathomechanism. This analysis is performed on two independent, multi-center patient data sets of lung (which is publicly available data) and bladder urothelial carcinoma. The presented results highlight the significance of computational pathology algorithms for predicting prognosis using H&E stained images alone and underpin the use of computational methods to improve the efficiency of clinical trial studies.

In gynaecological cancer AI project in iCAIRD, Artificial Intelligence (AI) algorithms were trained and evaluated on cervical biopsies for automated reporting of digital diagnostics with aim is to increase overall efficiency of pathological diagnosis and to have the performance tuned to high sensitivity for malignant cases. The algorithms were trained and validated on 1738 cervical WSIs. On the independent test set of 811 WSIs, the trained algorithm achieved 93.4% malignant sensitivity for classifying slides [5].

Methods

Data collection

The cervical tissue blocks were originally collected from the achieves of Glasgow Royal Infirmary (NG), Southern General Hospital (SG), Royal Alexandria Hospital (RAH) and Queen Elizabeth University Hospital (QEUH) (all in Glasgow, Scotland) each with independent tissue handling including fixation and tissue processing. The number of tissue blocks obtained from each of the above sites were: 829 from QEUH, 729 from NG, 647 from SG and 334 from RAH. Since some of these slides were faded new tissue sections were cut from the tissue blocks at one of two different thicknesses (3 microns or 4 microns) and then stained with one of four different H&E protocols (routine H&E, muscle biopsy protocol, neuro protocol and paediatric tissues protocol). Together, these combinations gave eight different labs maximising WSI variance and thereby decrease the likelihood of overfit to any one lab (combination of tissue processing, cutting and staining protocol). All the slides were scanned at QEUH using a Phillips Ultra Fast Scanner (UFS) with resolution equivalent to 40x or more specifically 0.25 microns/pixel, and stored in the iSyntax file format. WSIs were subsequently converted to OME-Tiffs format using Glencoe Software [6] to ensure compatibility with QuPath [7] (Version v0.2.3) for annotation. The code utilised for this conversion is publicly available from Zenodo [8]. The annotation procedure involved defining the main slide category, then manually annotating any additional subcategories that could have been available on the WSI.

Data split to train and test sets

The split percentages were calculated based on the case labels associated with the samples recorded in the system and the numbers per each set were agreed on by all the data scientist team members and the pathologists. All slides from two of the labs and 10% randomly selected slides from the other six labs were set aside as test set and never used in the training and validation process. The remaining 90% of the slides, from the 6 other labs were used as training set.

Table 1. Distribution of samples in training and test sets for cervical dataset.

Category	SubCategory	Count	Training	Test	Total
Malignant	- Squamous carcinoma	268	184	81	520
	- Adenocarcinoma	107	69	38	
	- CGIN	92	60	32	
	- Other*	59	44	15	
	Total		360	166	
High Grade	- CIN 2	320	212	108	641
	- CIN 3	321	221	100	
	Total		433	208	
Low Grade	- HPV	420	293	127	782
	- CIN 1	362	253	109	
	Total		546	236	
Normal/inflammation	- Normal/inflammation	590	399	131	590
	Total		1738	801	2539

* Other subcategory in malignant are biopsies with a malignant diagnosis that don't fall under adenocarcinoma or squamous carcinoma. Examples are involvement of the cervix by endometrial tumours or metastases spread from tumours in other parts of the body or other types of malignant tumour that are not carcinoma (e.g. sarcoma).

To retain the same proportion of classes in the train and test sets that were present in the entire original dataset, the dataset was split in stratified fashion balanced over categories, subcategories and staining by different laboratories for training and test sets. During the annotation process labels were double-checked and by independent pathologists in approximately 5% of the cases the final label associated with the scanned slide differed. This was either because the new slice taken from the biopsy tissue block did not show the same pathological features as the original slide, or that the original label had been inaccurately recorded. The corrected labels, that is the consensus label agreed after annotation were used for training and testing. This means the final numbers of slides of each type in Table 1 may not match the original proportions of cases selected for study.

Annotation process

Each slide was randomly assigned to one of four participating Consultant Pathologists for annotation. Each of the participating pathologists had a sub-specialist interest in Gynaecological Pathology, and participated in the UK National Gynaecological Pathology External Quality Assurance Scheme. Primary annotation was performed either by one of the four pathologists, or by a biomedical scientist, specifically trained for this project. All annotations done by a biomedical scientist were checked and signed off by one of the study pathologists. Where there was discrepancy, a third reviewers was used and a consensus agreed.

The annotation process stratified slides into four main diagnostic categories which include malignant, high grade, low-grade and Normal/inflammation. Each diagnostic category has subcategories. The categories and their sub-categories are defined as follows:

i. Malignant: Squamous cell cervical cancer and adenocarcinoma are the most common types of cervical cancer. Both of these are capable of local spread and metastasis. CGIN is an uncommon pre-invasive dysplastic lesion of glandular cells which can develop into AC. There is histological overlap with some well differentiated AC and this lesion tends to be treated more aggressively.

ii. High Grade: CIN is graded to determine risk of development of cancer and to guide further management. Most countries have now moved to a two tier classification for CIN (high grade and low grade). In the UK, pathologists still often refer to the old three tier classification (CIN1/2/3). For the purposes of this algorithm, we classified 'high grade' lesions as those with morphological features of CIN 2 or 3.

iii. Low Grade: A slide is labelled as low grade if it contains slightly abnormal cells on the surface of the cervix (CIN 1) or low-grade changes that are usually caused by an HPV infection (HPV). CIN 1 and HPV are not cancer and usually go away on their own without treatment, but sometimes they can become cancer and spread into nearby tissue.

iv. Normal/inflammation: Cervicitis is inflammation of the cervix. Cervicitis is common and may be caused by a number of factors, including infections, chemical or physical irritation, and allergies. Both normal tissue and cervicitis fall within this category which is not malignant.

Total of 2539 whole slide images (WSIs), with only one slide per patient, in iSyntax format, an annotation file per WSI in JSON format and a metadata file containing formations about each files, such as categories, subcategories, staining sites, etc was delivered at the end of the annotation process.

Figure 1 shows examples of overlaying annotations on the thumbnail of the image (downsampled whole slide images at level 5) for different categories.

Inter-observer variations

Despite the establishment of strict guidelines by the Bethesda System 2001 (TBS 2001) for reporting cervical smears, intra- and inter-observer variations remain unavoidable. These variations are inherent to the diagnostic process and can introduce complexities in training AI models, particularly for grading Cervical Intraepithelial Neoplasia (CIN). CIN represents a morphological continuum, but biopsies are categorised into two or three distinct grades, making consistency in diagnosis challenging .

To assess inter-observer variability, 200 cervical biopsy samples were independently re-annotated by three pathologists involved in the original annotation process. The results indicate that disagreements among pathologists were more pronounced at the subcategory level, sometimes leading to disagreements in overall category classification [5].

To evaluate the reliability of annotations, Cohen's kappa statistic was used to measure agreement between observers. For multiple observers, Cohen's kappa was calculated for each pair and averaged. The analysis revealed a Cohen's kappa score of 89.56 % for categories and 87.24% for subcategories, indicating a high level of agreement among observers [5].

Data validation and quality control

The images submitted were obtained directly from cases undergoing clinical histopathological diagnosis and were subject to rigorous scrutiny by the specialist team of diagnostic histopathologists who undertook the manual annotations of selected features. The diagnosis was available from the original clinical report and so annotation was a further confirmation of features already commented upon

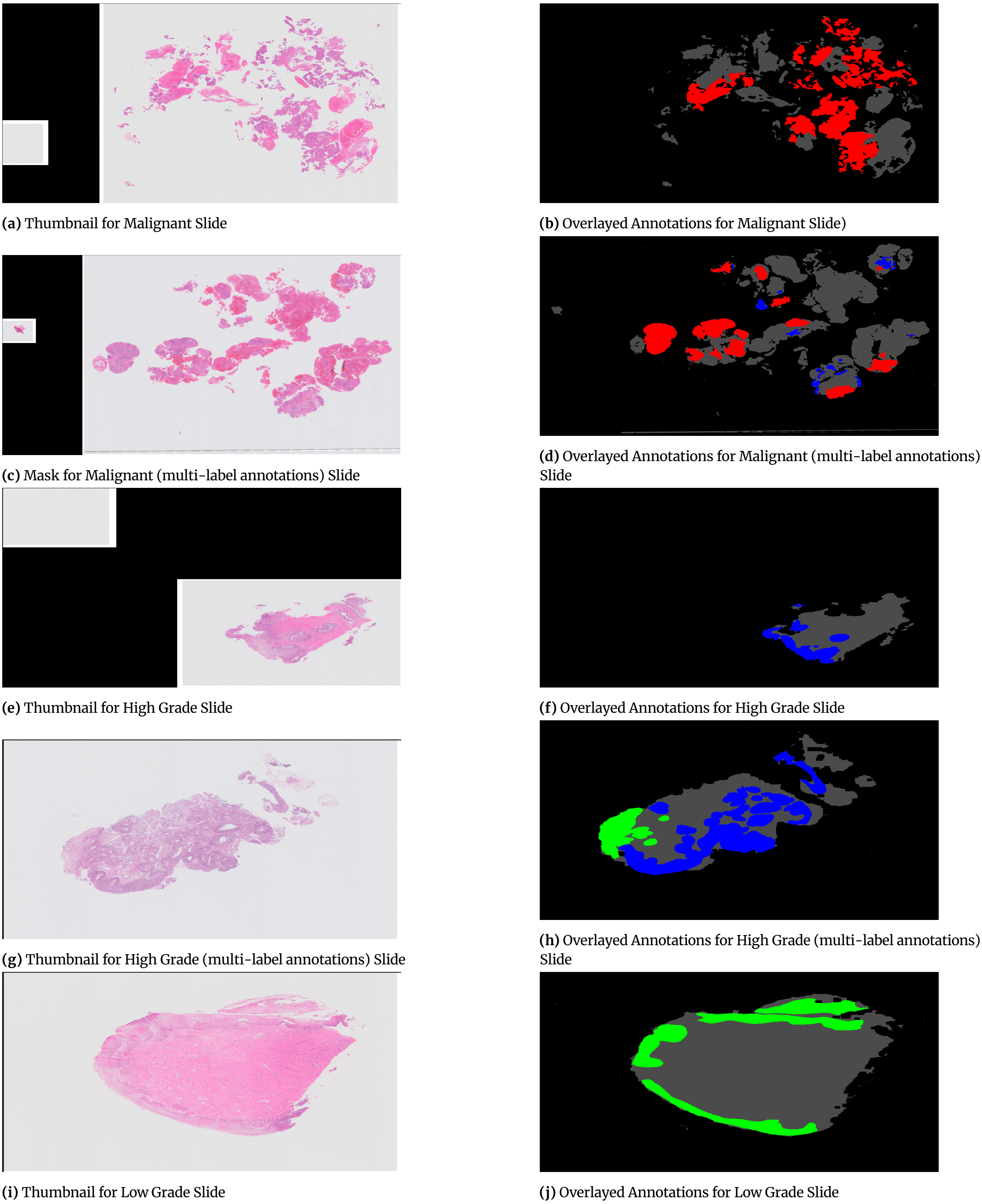

(a) Thumbnail for Malignant Slide

(b) Overlayed Annotations for Malignant Slide)

(c) Mask for Malignant (multi-label annotations) Slide

(d) Overlayed Annotations for Malignant (multi-label annotations) Slide

(e) Thumbnail for High Grade Slide

(f) Overlayed Annotations for High Grade Slide

(g) Thumbnail for High Grade (multi-label annotations) Slide

(h) Overlayed Annotations for High Grade (multi-label annotations) Slide

(i) Thumbnail for Low Grade Slide

(j) Overlayed Annotations for Low Grade Slide

Figure 1. Examples of overlaying annotations on the down-samples whole slide images

Malignant High Grade Low Grade Normal Tissue Background

by an expert specialist. Annotations were added afterwards as a separate exercise, not linked to primary clinical diagnosis. The gold standard was the pathologists' diagnosis and where there were discrepancies, by consensus review.

Using H&E cervical WSI dataset and their annotations, ML algorithms can be applied to assist in various aspects of cervical health analysis. Data collection and preprocessing is the first step in illustrating how ML algorithms can utilise this data, described in [5].

Table 2. Distribution of samples in training, validation, and test sets for cervical dataset in iCAIRD gynaecological cancer AI project.

Category	SubCategory	Count	Training	Validation	Test
Malignant	- Squamous carcinoma	268	127	60	81
	- Adenocarcinoma	107	243	23	38
	- CGIN	92	41	19	32
	- Other*	59	29	15	15
High Grade	- CIN 2	320	141	71	108
	- CIN 3	321	146	75	100
Low Grade	- HPV	420	197	96	127
	- CIN 1	362	169	84	109
Normal /inflammation	- Normal /inflammation	590	268	191	131
	Total	2539	1164	574	801

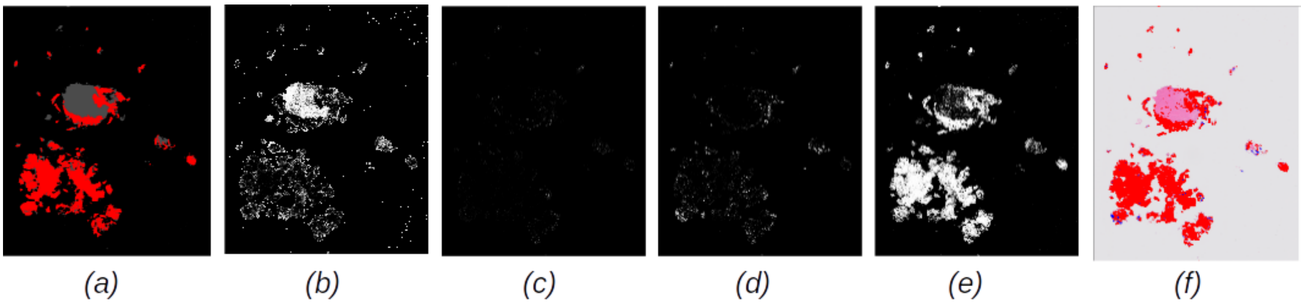

Figure 2. Patch level heatmaps for a malignant slide
(a):Truth Label (b): Normal (c):Low Grade (d): High Grade (e): Malignant (f): Prediction
Malignant High Grade Low Grade High Probability Normal Tissue Background

Gynaecological Cancer AI project

This dataset as discussed earlier was originally created for Gynaecological Cancer AI project which is part of iCAIRD. The dataset preparation, cleaning and the annotation process of this dataset is described in details in [5]. In this study two thirds of the data described in Table 1 was used as training and validation set. Table 2 illustrates the number of slides for each subcategory and category in training, validation and test sets for this study. A patch-level classifier was then trained on the patches extracted from the WSIs in the training set and evaluated on the patches in the validation set. Predictions are probabilities per category for each patch on the slide. A binary heatmap is generated per category per slide using the patch probabilities. Higher probabilities are shown as brighter pixels in a heatmap. The computed probabilities are used to compute the final prediction at patch level for each slide and to create the patch level confusion matrices for training and validation datasets. The features extracted from the heatmaps generated at patch level are used for training a ML classifier to form the final slide level predictions for each slide.

Figure 2 shows patch level generated heatmaps for a malignant slide. The code and the trained models for this project is available at [8].

Extracting nuclear morphological features using Indica Halo AI

In another experiment, WSI images were imported into Indica HALO and HALO AI (v.3.6.4134), along with corresponding annotation files. A nuclei segmentation classifier, underpinned by advanced deep learning neural network algorithms, was trained with examples from multiple different cases as shown in Figure 3 for different cases. An analysis algorithm, Multiplex IHC v.3.2.3 was utilised to segment individual nuclei to extract nuclear morphological features such as area, perimeter, and roundness within the annotation. The tabular data from the individual nuclear morphological features, along with their x and y coordinates, was exported into CSV file format. Multiplex IHC analysis algorithm was used to segment individual nucleus and to extract its morphological features in four different annotations such as normal, low grade, high grade, and malignant. The data split for this project was done based on the original data split in Table 2 with only difference that the validation set has been added to training set and we just have training and test sets for the experiment. Different machine learning algorithms (Decision Trees, SGD and Random Forest classifier) were trained on the morphological features extracted and finally the trained model was tested with the features extracted from the slides in the test set.

Re-use potential

In some areas of diagnostic histopathology large datasets have been made available, particularly in breast and colon cancer, where they have spawned competitions and collaborations to rapidly improve algorithms as well as providing real world data for researchers and students. The current dataset is unique, and includes a spectrum of histological changes in annotated slides of cervical disease, that can be used for training algorithms, workshops and validation of pre-existing algorithms with reference to cervical abnormalities, from normal, through dysplasia to carcinoma. The annotations have been applied by expert pathologists and thus can be used for training to identify particular features that are already labelled. Furthermore, inclusion of nuclear morphological features obtained by AI-enabled image analysis may augment accuracy in distinguishing between normal, low-grade, high-grade, and malignant conditions. Cervical disease remains a worldwide problem and biopsies are frequently small, poorly orientated and can be a significant part of a laboratory's clinical workload. Improving accuracy, increasing workflow and enabling selection of high risk cases for urgent attention would make a considerable difference to a pathologist's working day.

Data availability

All cervical whole slide images, their annotation files, binary masks and a metadata file (2539 images in iSyntax format, 2539 annota-

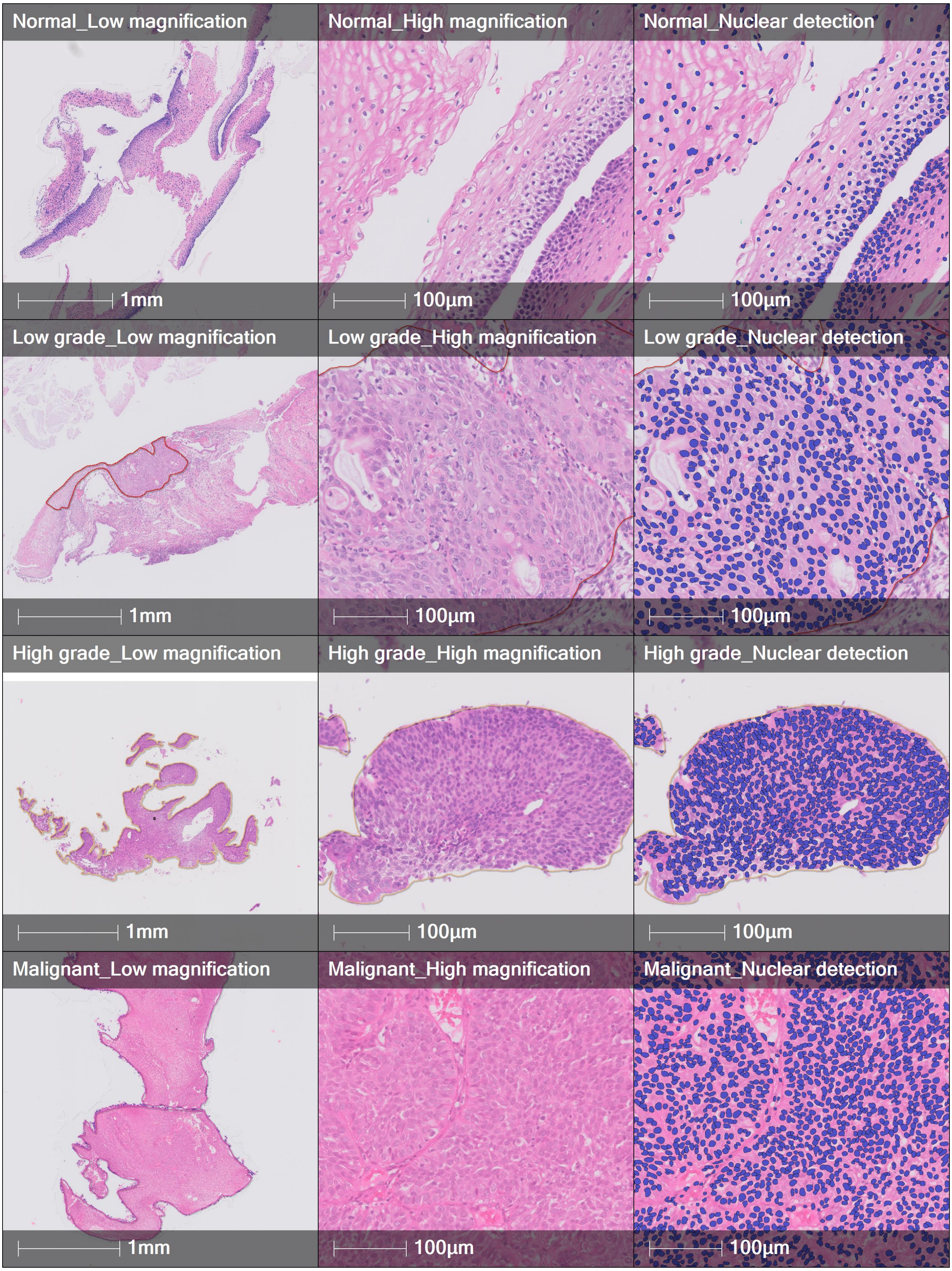

Figure 3. Examples of segmented nuclei (colored blue) of different categories using Indica HALO AI platform

tion files in JSON format, 2539 binary masks in PNG format and a metadata file in CSV format) and the morphological features extracted from them in Halo are openly available in the GigaScience repository, GigaDB [S-BIAD1168] [9].

Declarations

List of abbreviations

- Artificial Intelligence (AI)
- Cervical Intraepithelial Neoplasia (CIN)
- Cervical Glandular Intraepithelial Neoplasia (CGIN)
- Giga Byte (GB)
- Graph Based Unsupervised Learning (GBUL)
- Human Papilloma Virus (HPV)
- Industrial Centre for Artificial Intelligence Research in Digital Diagnostics (iCAIRD)
- Machine Learning (ML)
- Quantitative Pathology (QuPath)
- Whole Slide Image (WSI)

Ethical Approval

- Ethics approval for the study was granted by NHS Greater Glasgow and Clyde Biorepository and Pathology Tissue Resource (REC reference 16/WS/0207) on 4th April 2019.
- Biorepository approval was obtained (application number 511)
- Local approval was obtained from the School of Computer Science Ethics Committee, acting on behalf of the University Teaching and Research Ethics Committee (UTREC) [Approval code-CS15840].

Consent for publication

Not applicable.

Competing Interests

The authors declare that they have no competing interests.

Funding

This work is supported by the Industrial Centre for AI Research in digital Diagnostics (iCAIRD) which is funded by Innovate UK on behalf of UK Research and Innovation (UKRI) [project number: 104690], and in part by Chief Scientist Office, Scotland.

Author's Contributions

Mahnaz Mohammadi wrote the manuscript and supervised data preprocessing, together with Christina Fell and In Hwa Um.
Prishma Shahi imported annotations in Indica Halo AI platform and measured nuclear morphological features.
Gareth Bryson initiated the project, and Sarah Bell, Sheeba Syed, and Prakash Konanahalli annotated the whole slide images. David Harris Birtill and Ognjen Arandjelovic supervised machine learning experiments.
Clare Orange arranged data release from Glasgow Biorepository. James Blackwood oversaw governance procedures, established digital pathology services and supervised data de-identification and release. David Harrison is Director of iCAIRD, obtained funding, reviewed results and helped to draft the manuscript. All authors have seen and approved the manuscript.

Acknowledgements

We acknowledge the support of NHS Research Scotland (NRS) Greater Glasgow and Clyde Biorepository. We acknowledge the support of the biomedical scientists, Tim Prosser, Lucy Irving, Jennifer Campbell and Jennifer Faulkner, from the Pathology Department, NHS Greater Glasgow and Clyde for technical support.

Authors' information

MM, CF, and DM hold a PhD degree and are data scientists in the School of Medicine, University of St Andrews.
PS is a research technician at the School of Medicine, University of St Andrews.
Dr Gareth Bryson is a Consultant Pathologist and Clinical Director for Laboratory Medicine at the Queen Elizabeth University Hospital, Glasgow. Drs Sarah Bell, Prakash Konanahalli and Sheeba Syed are consultant gynaecological pathologists at Queen Elizabeth University Hospital, NHS Greater Glasgow and Clyde, UK. Ognjen Arandjelovic and David Harris Birtill are Reader and Senior Lecturer respectively in Computer Science, University of St Andrews.
In Hwa Um is a postdoctoral research fellow in pathology AI in the University of St Andrews. Clare Orange is Biorepository Manager in NHS Greater Glasgow and Clyde, and a doctoral candidate in the University of St Andrews.
James Blackwood is Chief Technical Officer of iCAIRD and Innovation Fellow in University of St Andrews. David Harrison is Professor of Pathology at the University of St Andrews, and Director of iCAIRD.

References

1. Gynaecological Cancer AI.;. https://icaird.com/wp9-gynaecological-cancers/.
2. Li C, Chen H, Li X, Xu N, Hu Z, Xue D, et al. A review for cervical histopathology image analysis using machine vision approaches. Artificial Intelligence Review 2020;53:4821–4862.
3. Li C, Hu Z, Chen H, Ai S, Zhang J, Zhang Y, et al. A cervical histopathology image clustering approach using graph based features. SN Computer Science 2021;2:1–20.
4. Qaiser T, Lee CY, Vandenberghe M, Yeh J, Gavrielides MA, Hipp J, et al. Usability of deep learning and H&E images predict disease outcome-emerging tool to optimize clinical trials. NPJ precision oncology 2022;6(1):37.
5. Mohammadi M, Fell C, Morrison D, Syed S, Konanahalli P, Bell S, et al. Automated reporting of cervical biopsies using artificial intelligence. PLOS Digital Health 2024;3(4):e0000381.
6. Mellisa Linkert, Chris Allan, Converting Whole Slide Images to OME-TIFF: A New Workflow; 2019. https://www.glencoesoftware.com/blog/2019/12/09/converting-whole-slide-images-to-OME-TIFF.html, Last accessed on 2022-08-12.
7. Bankhead P, Loughrey MB, Fernández JA, Dombrowski Y, McArt DG, Dunne PD, et al. QuPath: Open source software for digital pathology image analysis. Scientific reports 2017;7(1):1–7.
8. Fell C, Mohammadi M, Morrison D. StAndrewsMedTech/icairdpath-public: Release for publication 2023 Feburary;https://zenodo.org/record/7674764.
9. In Hwa Um CFDMSBGBSSPKCOPS Mahnaz Mohammadi, Harrison D, Cervical Whole Slide Images Dataset ;.

Click here to access/download;Figure;IC-CX-00027-01_mask.png

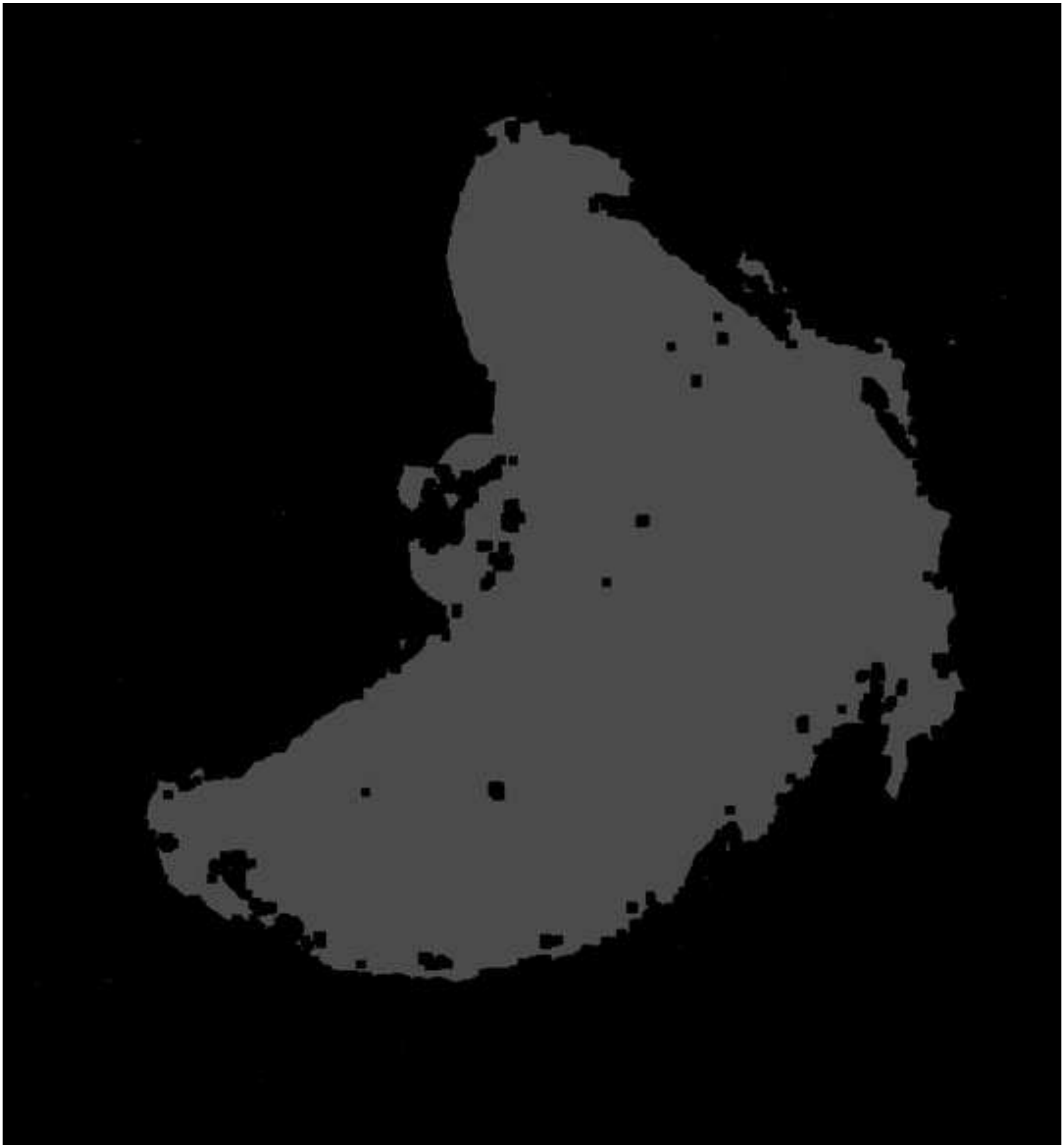

Click here to access/download;Figure;IC-CX-00027-01_thumbnail.png

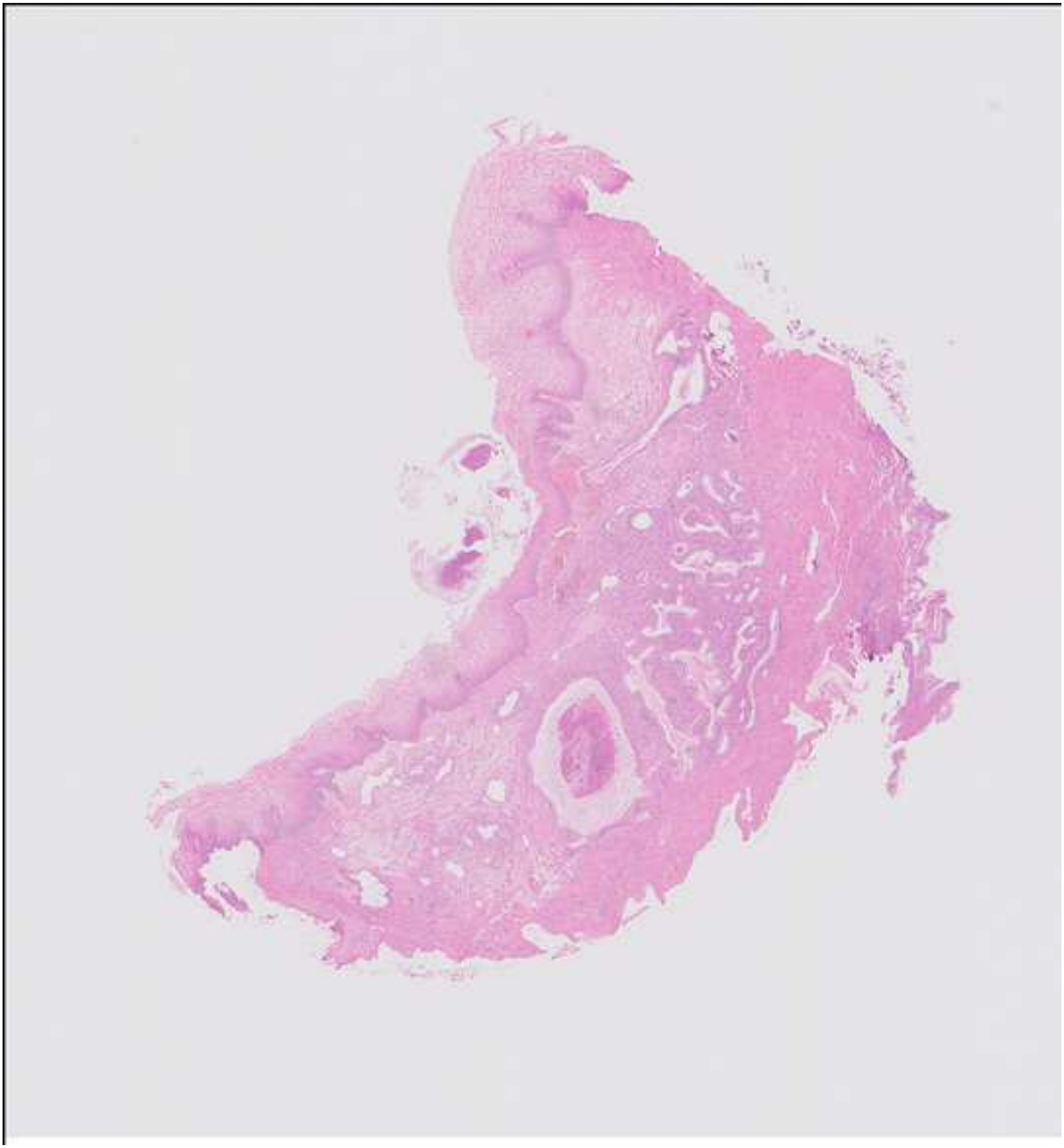

Click here to access/download;Figure;IC-CX-00029-01_mask.png

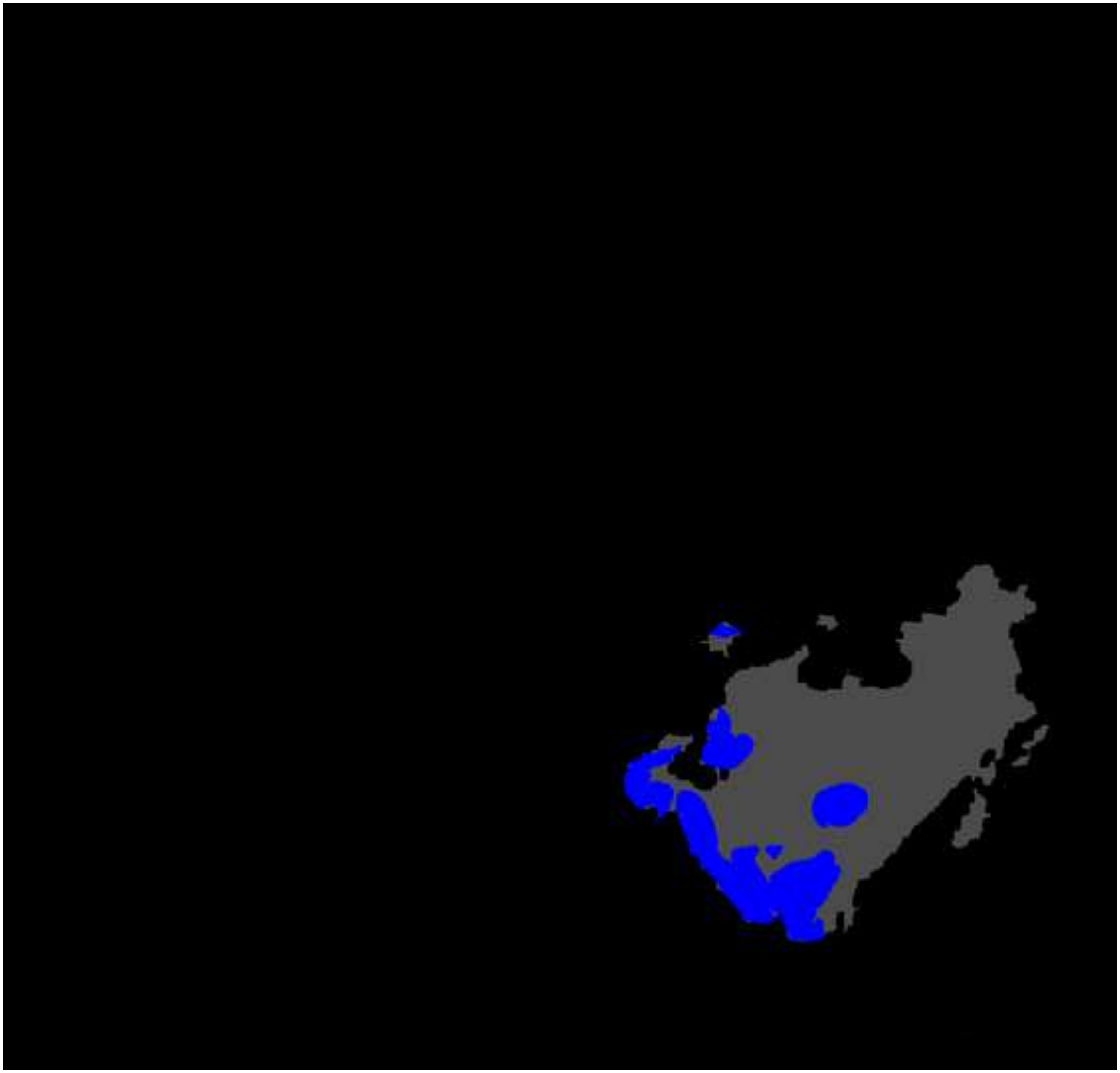

Click here to access/download;Figure;IC-CX-00029-01_thumbnail.png

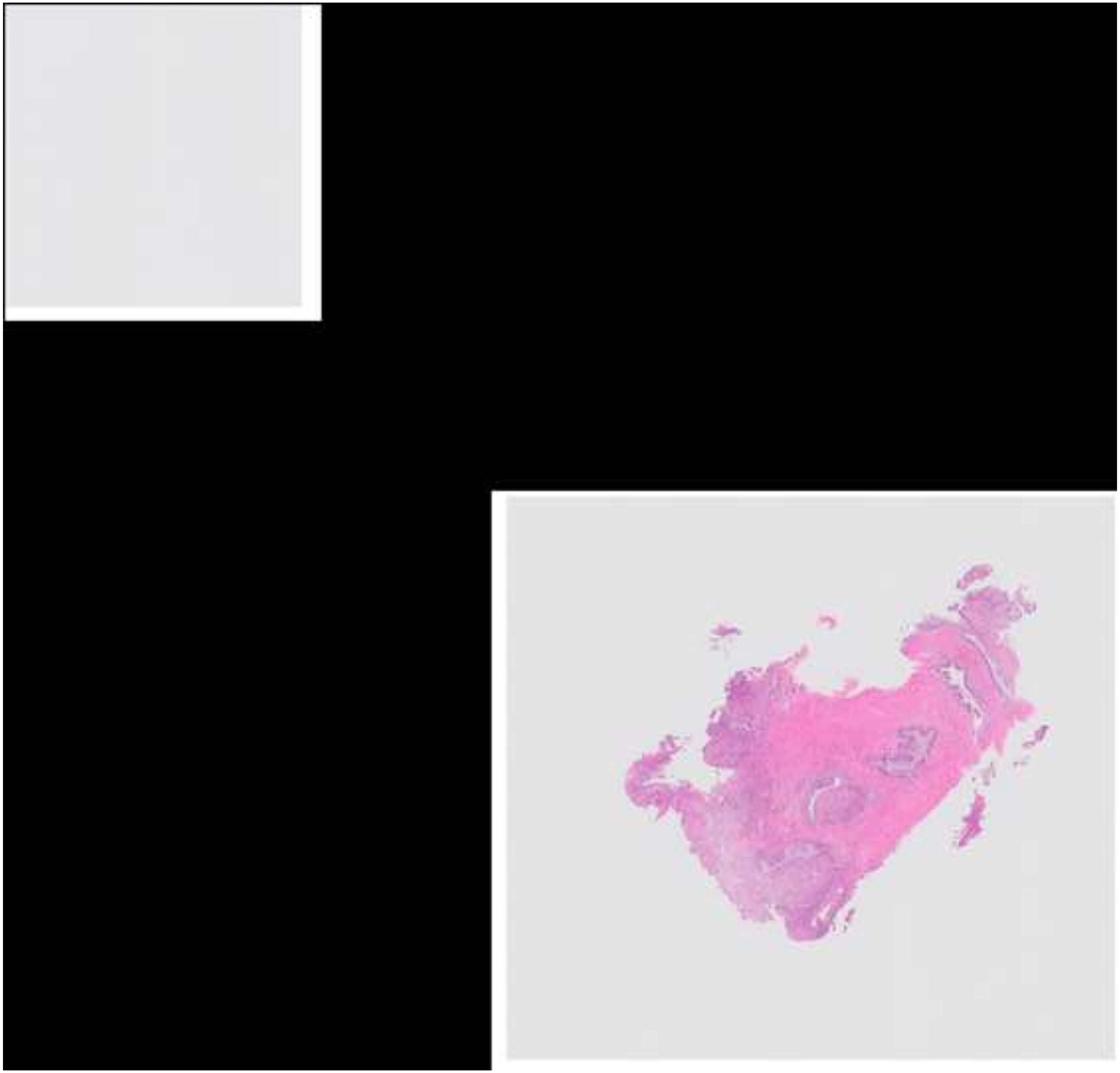

Click here to access/download;Figure;IC-CX-00049-01_mask.png

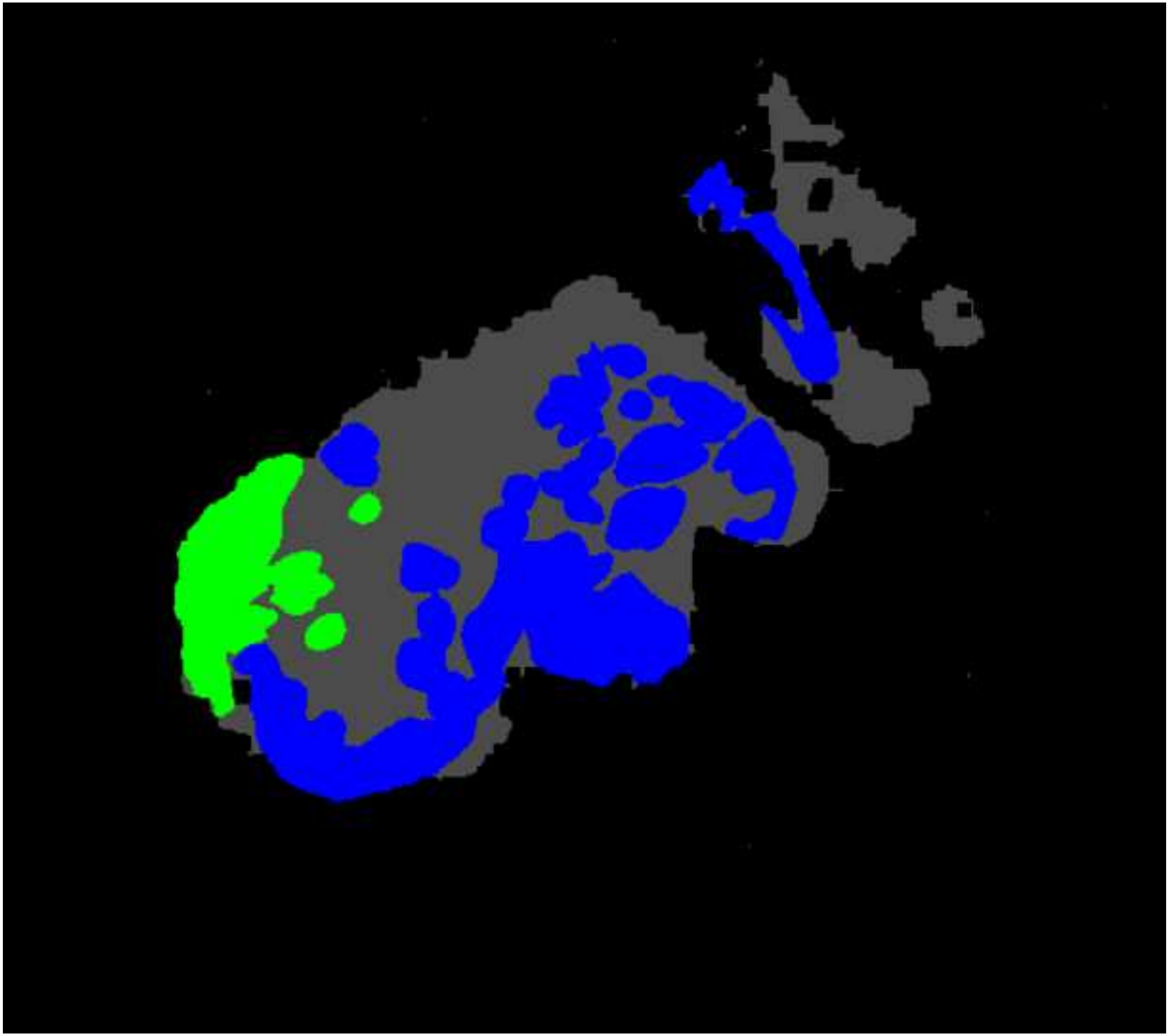

Click here to access/download;Figure;IC-CX-00049-01_thumbnail.png

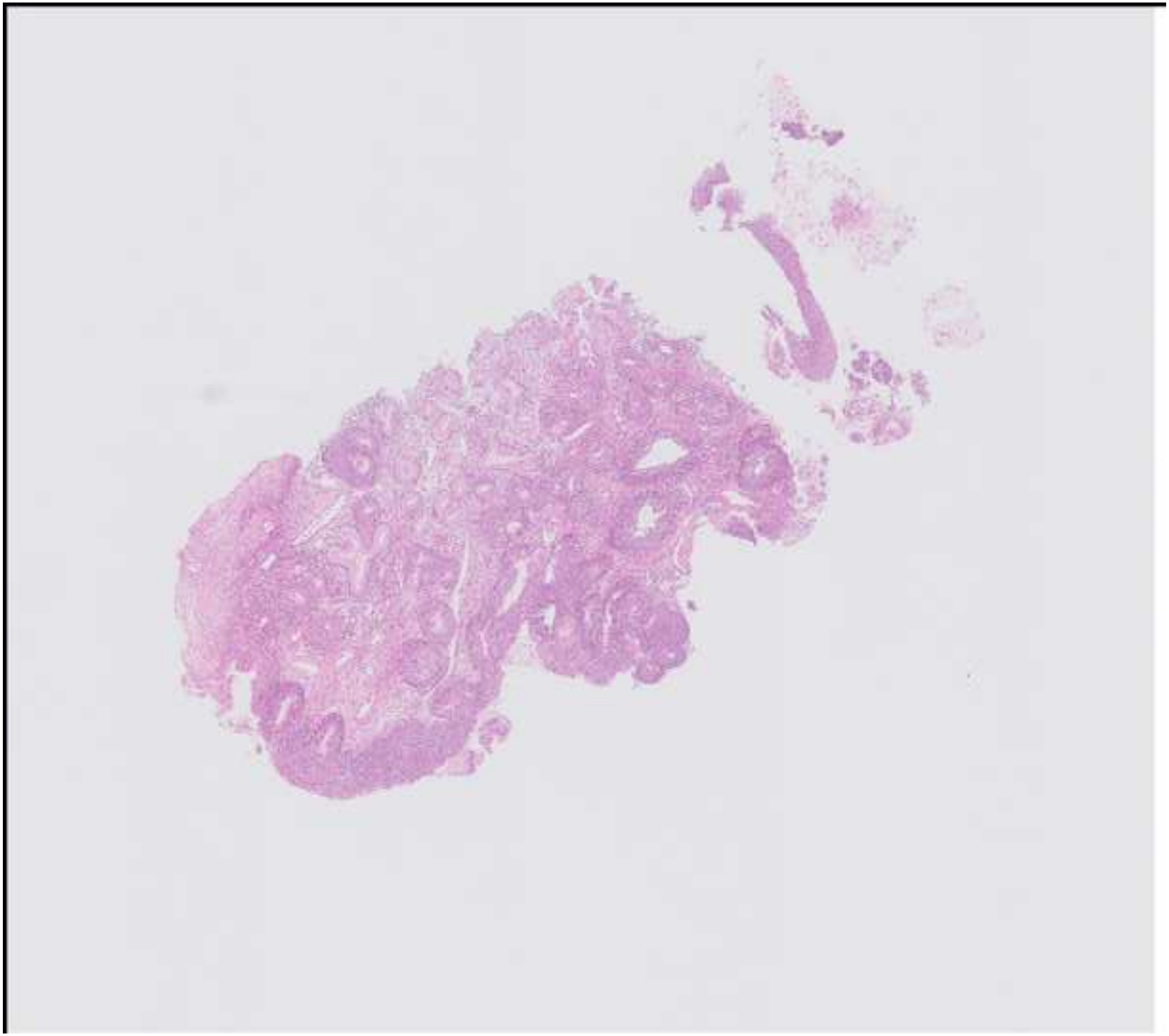

Click here to access/download;Figure;IC-CX-00053-01_mask.png

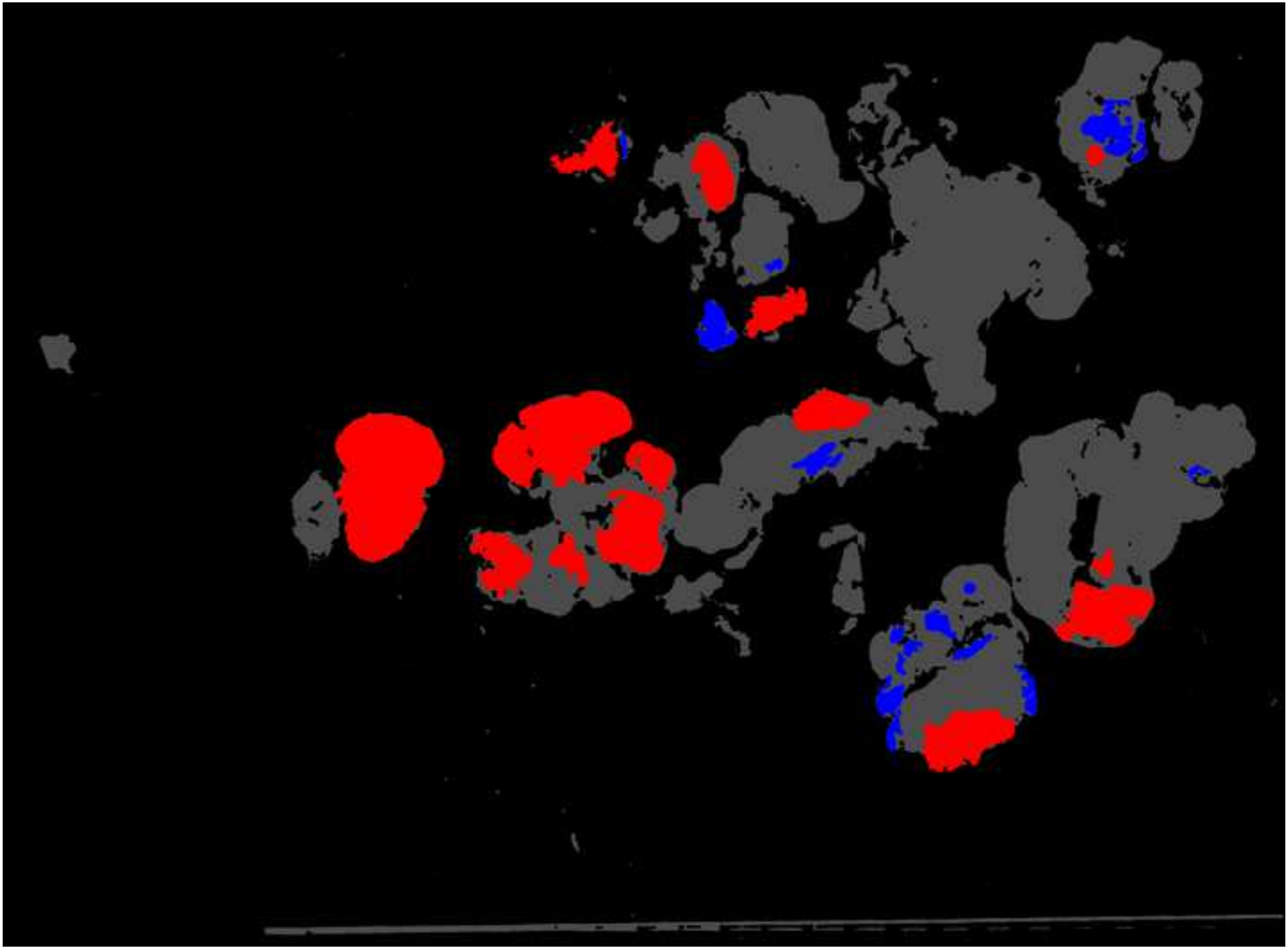

Click here to access/download;Figure;IC-CX-00053-01_thumbnail.png

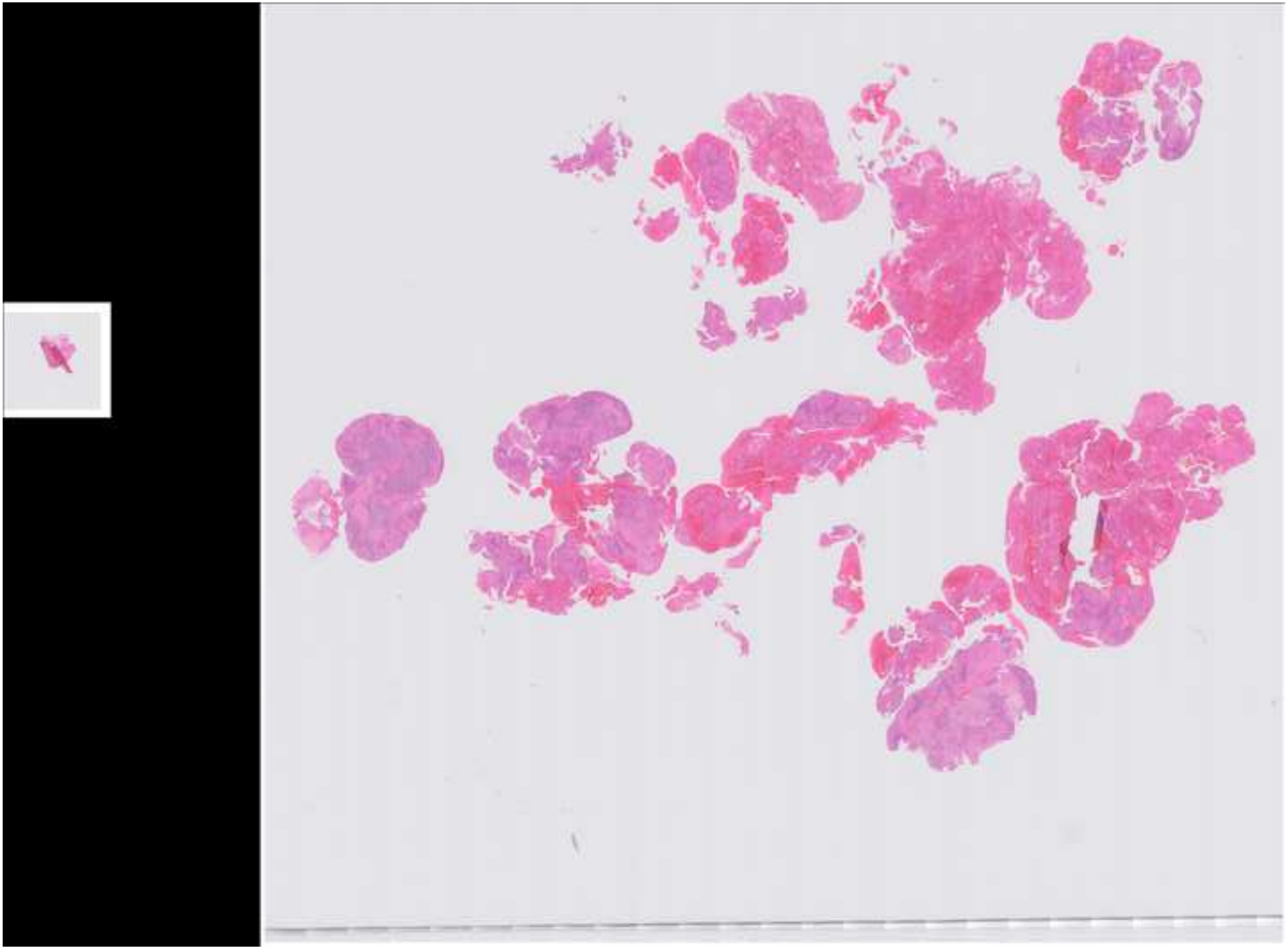

Click here to access/download;Figure;IC-CX-00129-01_mask.png

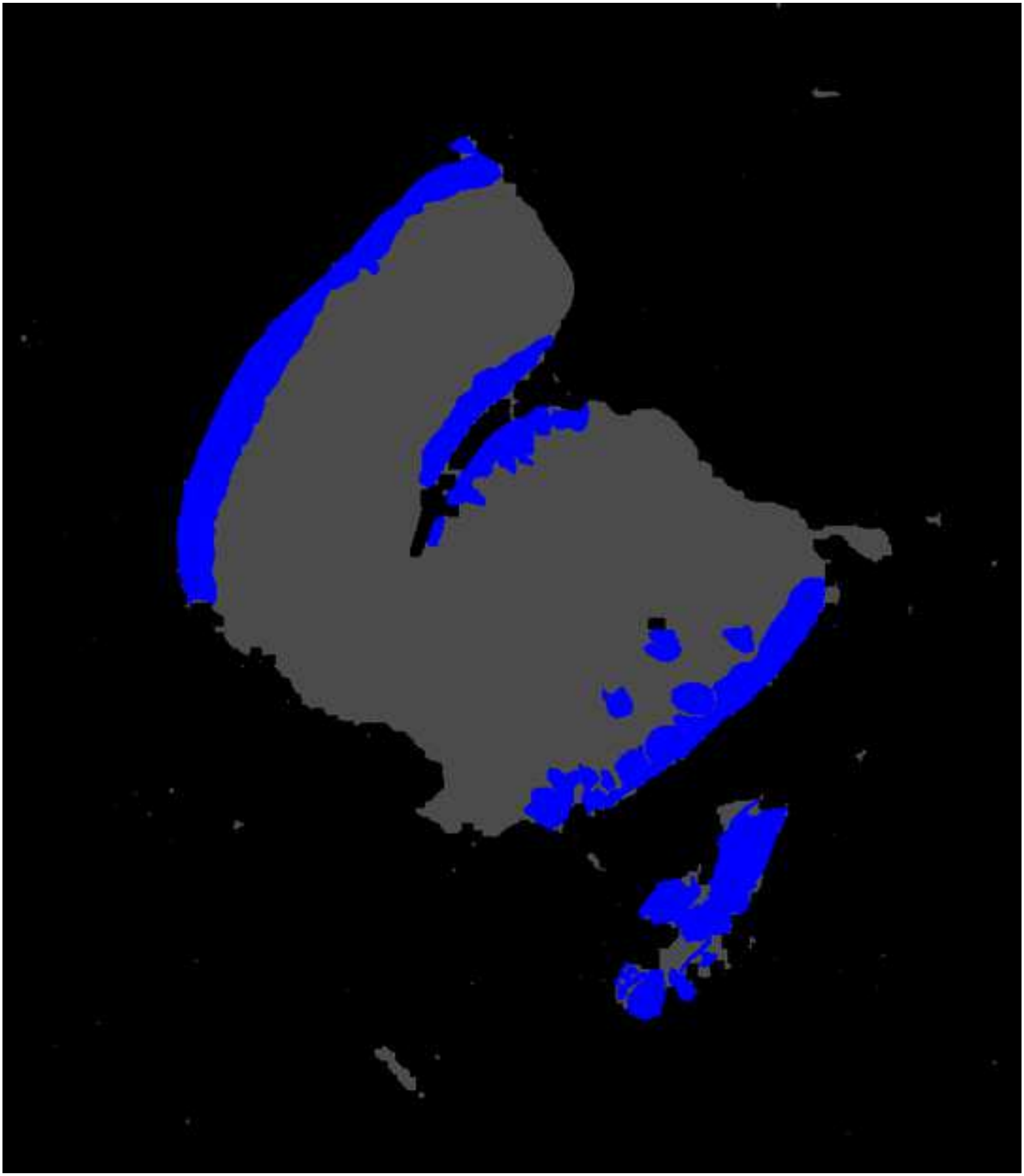

Click here to access/download;Figure;IC-CX-00129-01_thumbnail.png

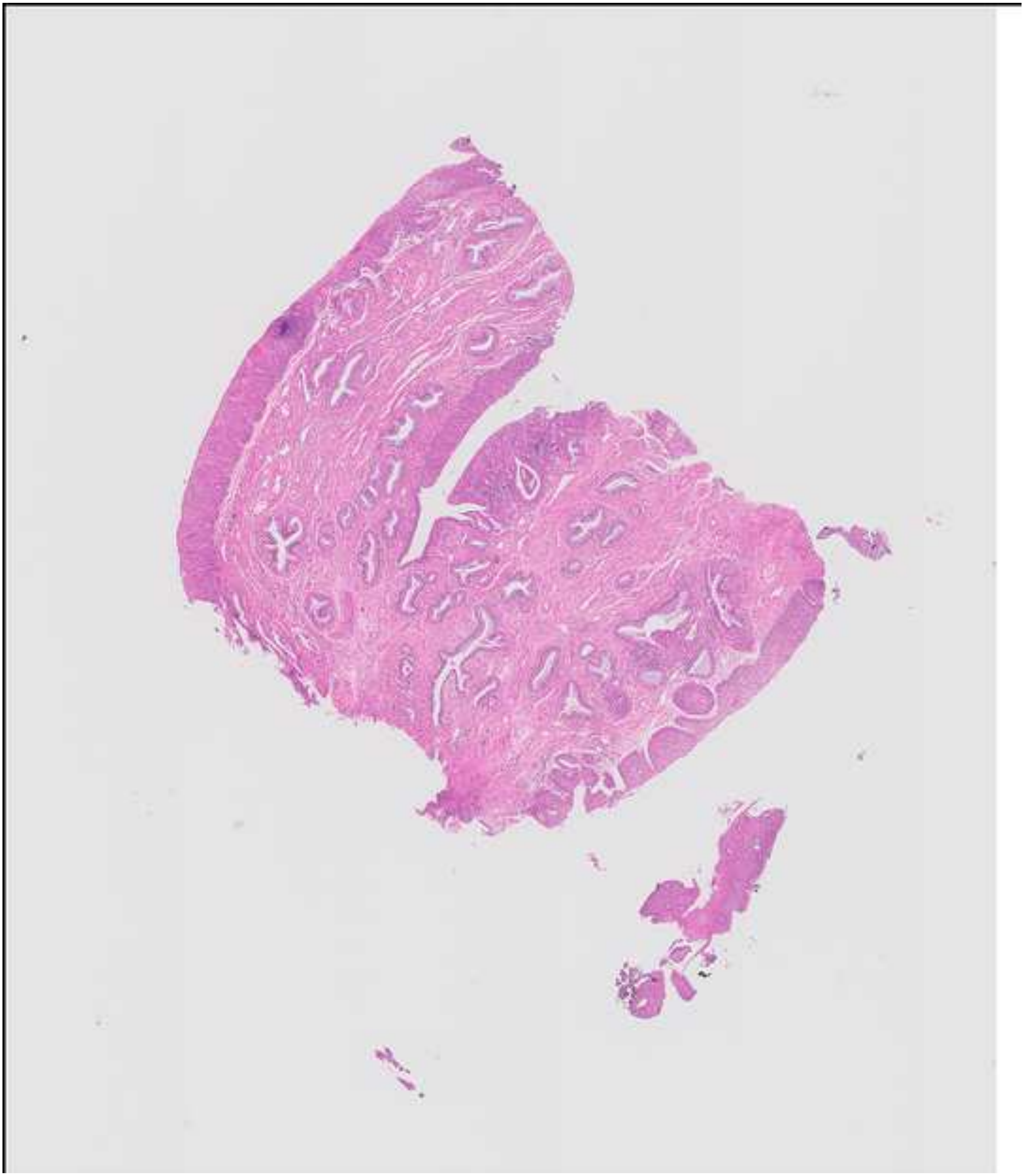

Click here to access/download;Figure;malignant_heatmap.png

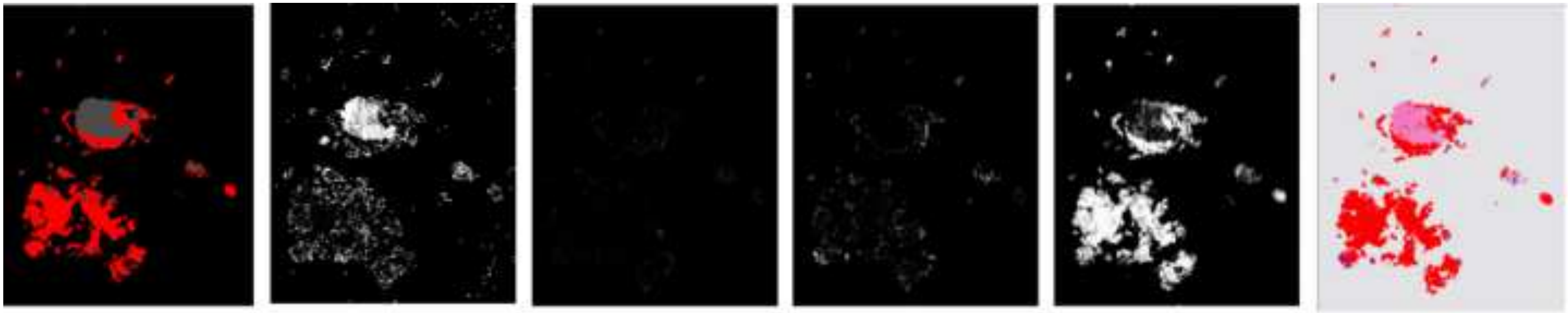

(a) (b) (c) (d) (e) (f)

Click here to access/download;Figure;architecture.png

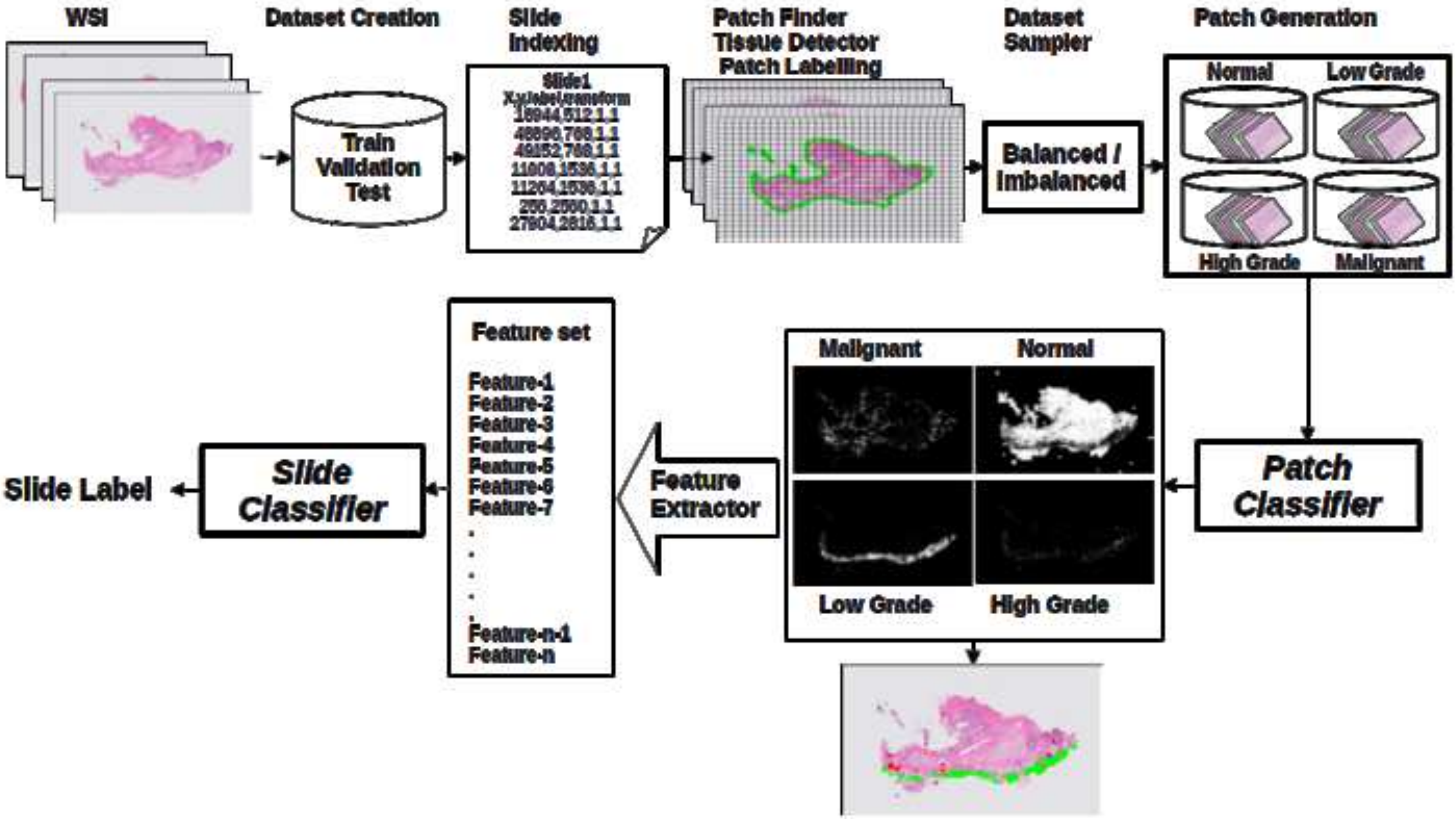

Click here to access/download;Figure;Figure_normal_LG_HG_malignant_HE

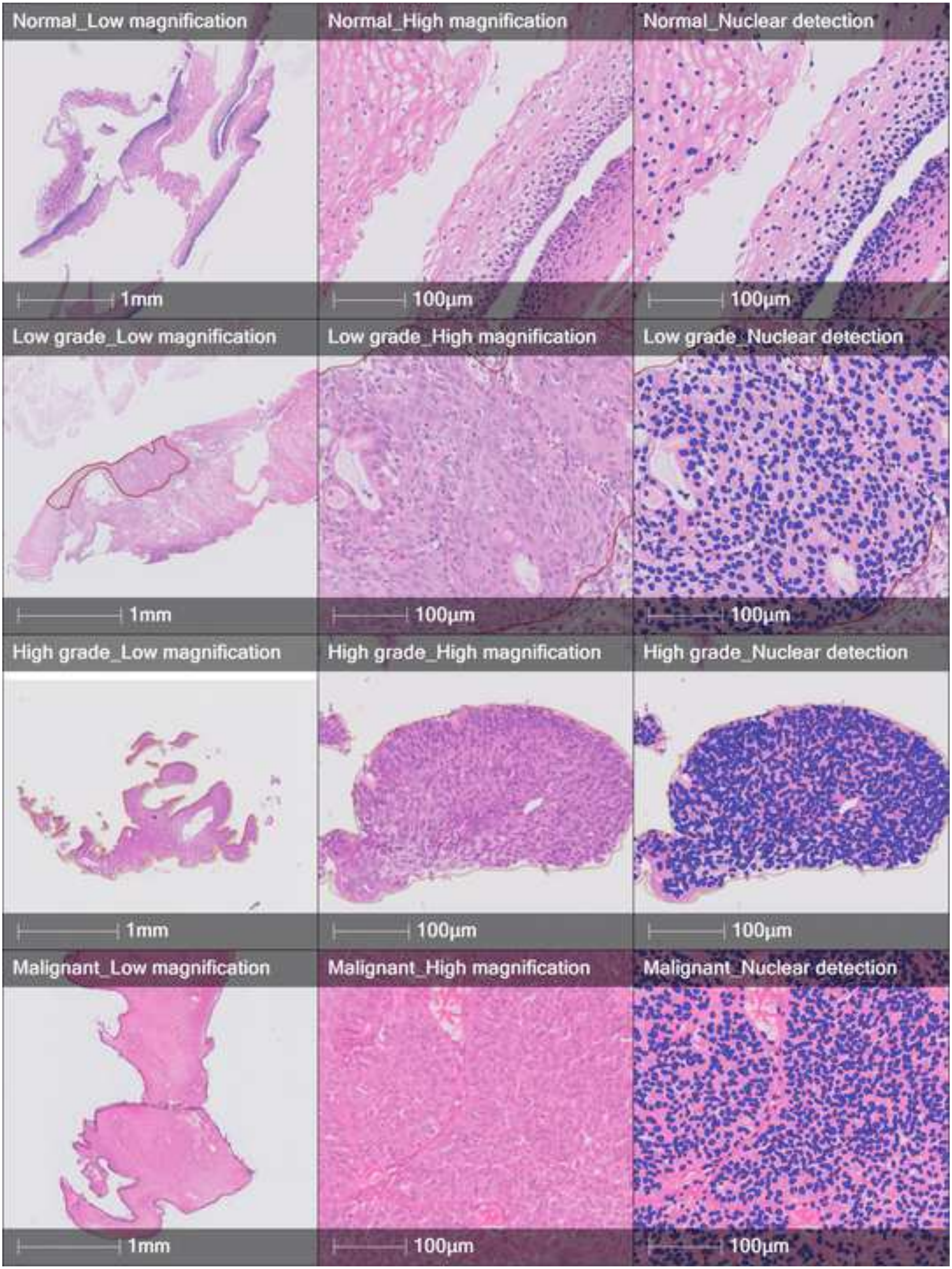

Click here to access/download;Figure;IC-CX-00012-01_mask.png

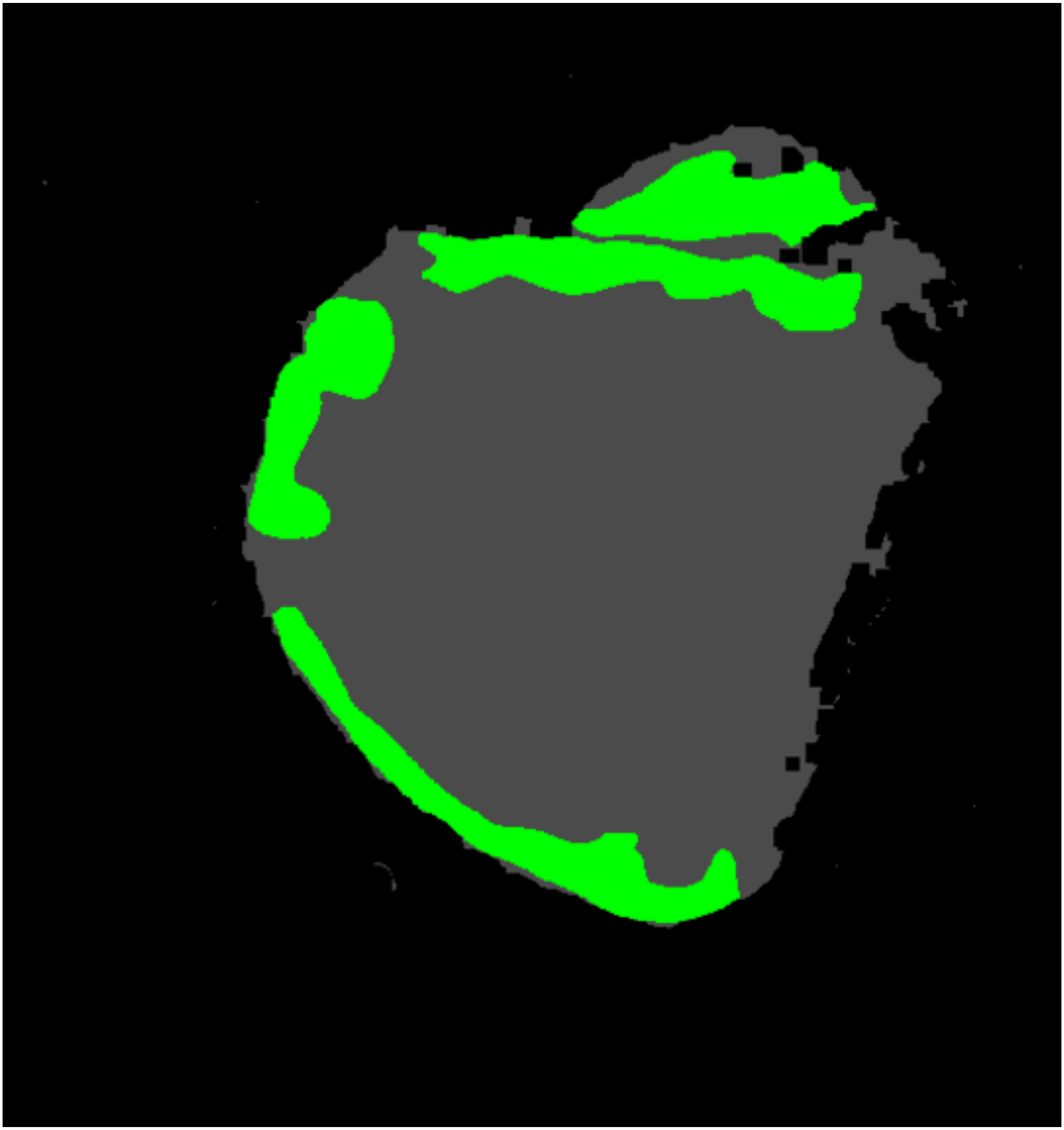

Click here to access/download;Figure;IC-CX-00012-01_thumbnail.png

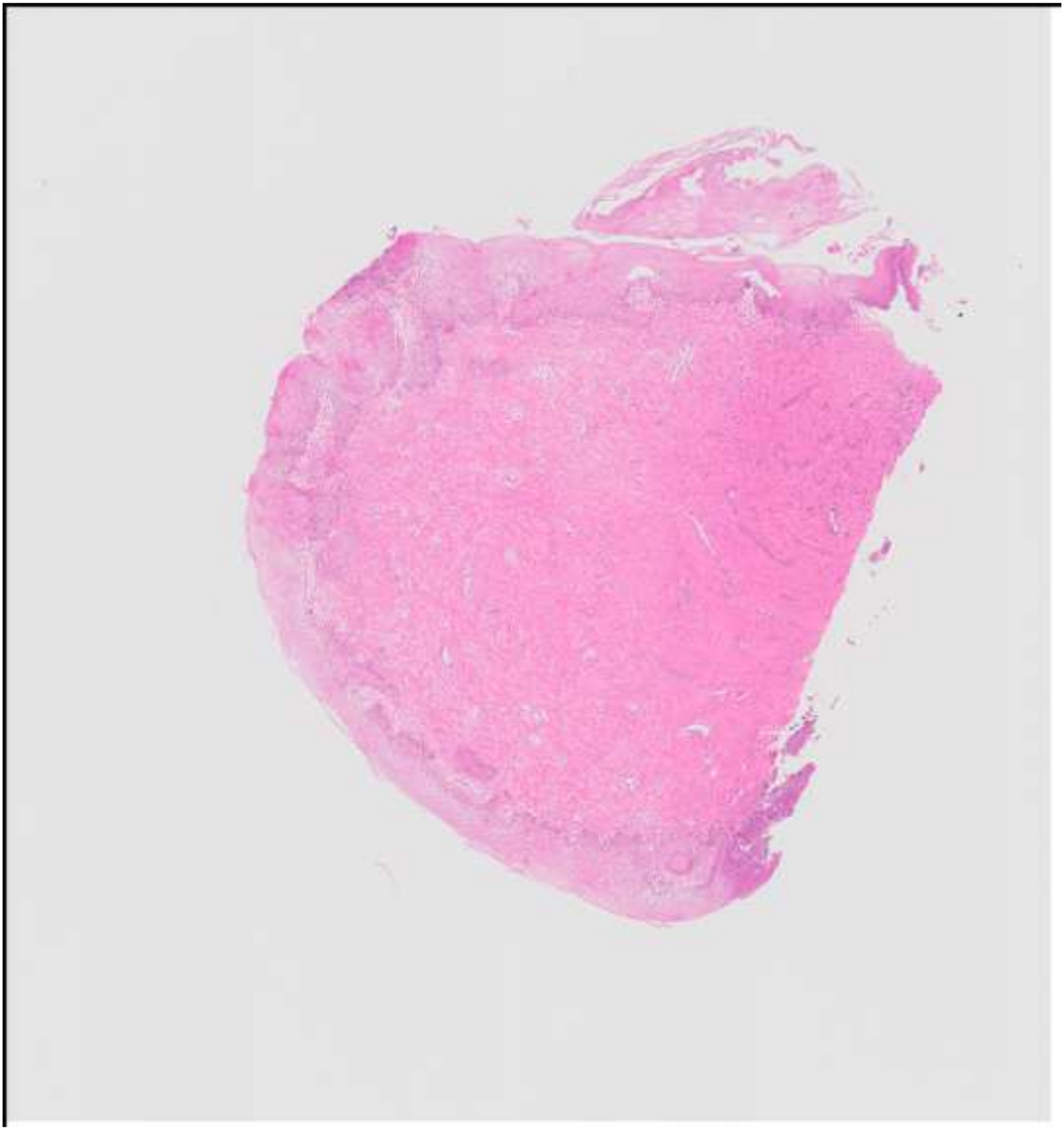

Click here to access/download;Figure;IC-CX-00020-01_mask.png

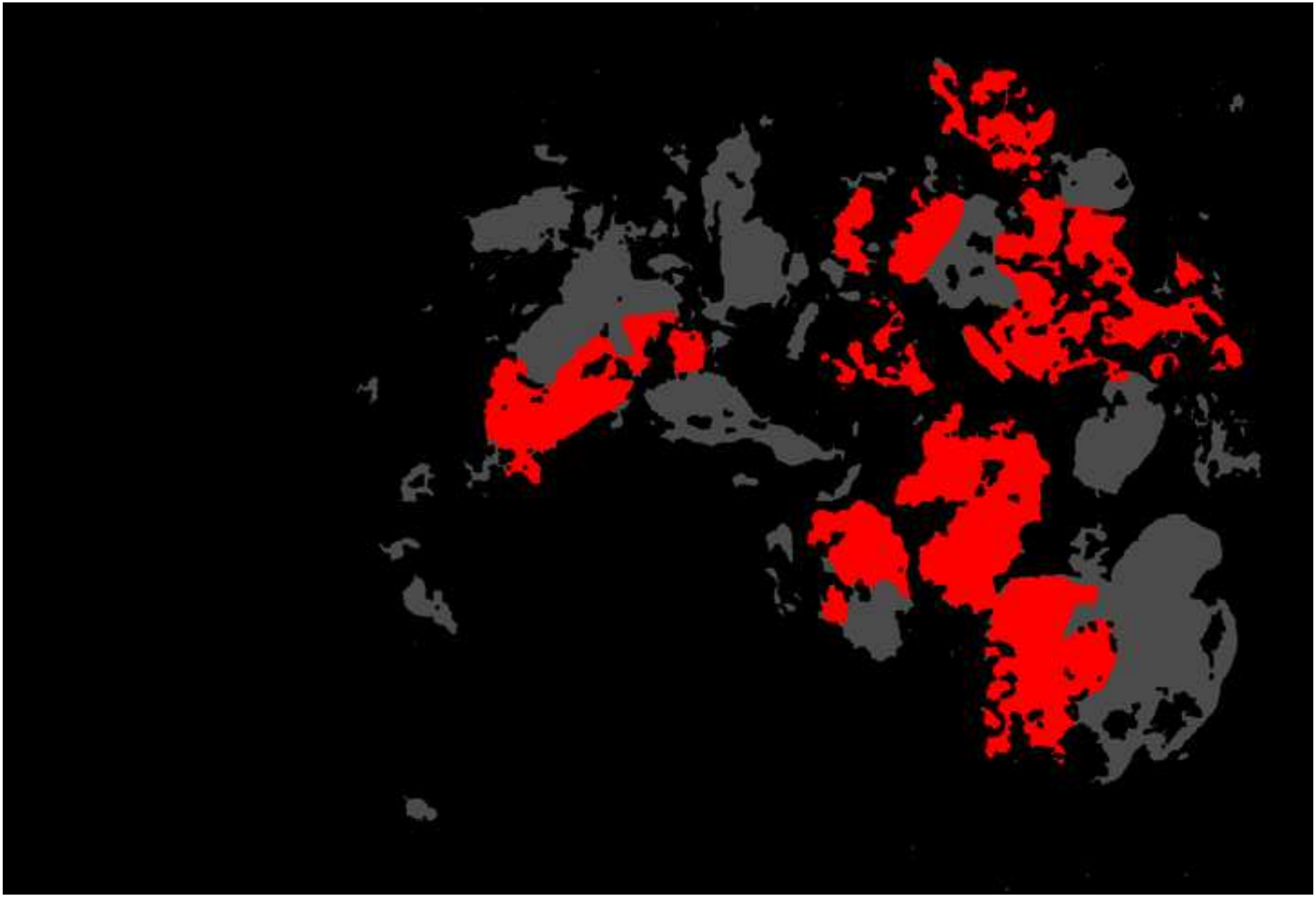

Click here to access/download;Figure;IC-CX-00020-01_thumbnail.png

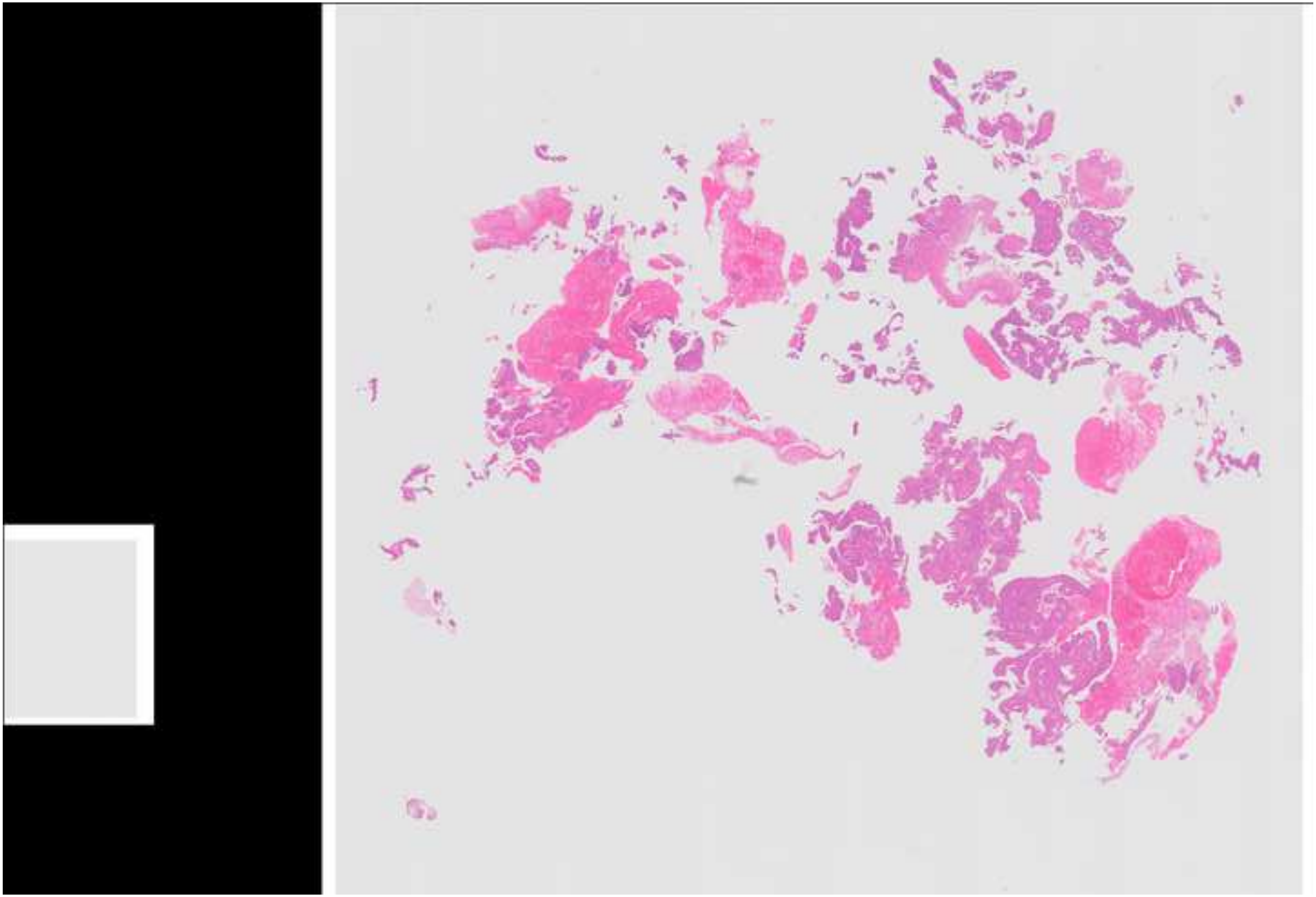

Personal Cover
Click here to access/download;Personal Cover;2nd comments Giga-cervical reviewers comments-29May25-Final.docx

Editor GigaScience

School of Medicine
University of St Andrews
North Haugh
St Andrews UK
KY16 9TF

29 May 2025

Dear Editor,

We would like to express our sincere thanks for the opportunity to revise and resubmit our manuscript entitled: **"Cervical Whole Slide Images Dataset for Multi-class Classification (ID: GIGA-D-24-00162)".** We appreciate the constructive feedback provided by the reviewers and the editorial team.

We have carefully considered all comments and have made corresponding revisions to the manuscript. Please find the attached our detailed point-by-point response to the reviewer's comments, along with a revised version of our manuscript

We hope that our revisions and clarifications we have provided sufficiently address all concerns and further enhance the quality and clarity of our work. We are grateful for the thoughtful suggestions and for the opportunity to improve our manuscript.

Thank you again for your time and consideration. We look forward to your feedback.

Yours faithfully,

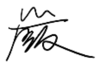

In Hwa Um

Reviewer #3:

14. ***Slide format:***
While I appreciate the discussion on this topic, my stance remains unchanged: a significant portion of the pathology and machine learning community relies on OpenSlide-compatible formats. This discrepancy may limit the dataset's impact (I have never used OME-TIFF but from what I can read, it is compatible with QuPath but not OpenSlide. Do you confirm?)
To address this, could the authors consider providing instructions or a Docker environment with a script to facilitate format conversion?
This issue is, in my opinion, the primary obstacle to the dataset's widespread adoption.

We appreciate the reviewer's continued attention to this important issue. We fully agree that the ISYNTAX file format poses limitations for interoperability within the digital pathology and machine learning communities, many of whom rely on OpenSlide-compatible formats. In response to this concern, we have made available a publicly accessible codebase that enables the conversion of ISYNTAX files to the widely adopted OME-TIFF format. This resource is hosted on Zenodo (Reference 8) and is now explicitly referenced in the *Data collection* section of the Methods.

To further enhance accessibility and reproducibility, we have also provided a Docker environment within the same Zenodo repository. This Docker setup includes all necessary dependencies and scripts required for the conversion process, thereby facilitating easy deployment across different computational environments. The Docker container can be accessed via the following DOI: https://doi.org/10.5281/zenodo.7674764

We confirm that while OME-TIFF is not directly supported by OpenSlide, it is compatible with widely used open tools such as QuPath, which supports annotation and visualisation functionalities. We hope that these additional resources and clarifications address reviewer's concerns and help ensure the dataset's broader usability and adoption.

15. ***Why do you believe so? I would appreciate a clear justification for the authors' belief in this regard. Increasing the number of centers in the training set may have this effect - but if some centers effectively correlate with the output variable, then having this same center in the test set will artificially inflate test results - and with the current dataset design, we lack means to know that. Please see : Howard, Frederick M., James Dolezal, Sara Kochanny, Jefree Schulte, Heather Chen, Lara Heij, Dezheng Huo, et al. « The Impact of Site-Specific Digital Histology Signatures on Deep Learning Model Accuracy and Bias ».*** Nature Communications 12, nº 1 (décembre 2021): 4423. https://eur01.safelinks.protection.outlook.com/?url=https%3A%2F%2Fdoi.org%2F10.1038%2Fs41467-021-24698-1&data=05%7C02%7Cdjh20%40st-andrews.ac.uk%7C4e289b4df86f43b8505508dd5da84e77%7Cf85626cb0da849d3a

a5864ef678ef01a%7C0%7C0%7C638769699632897702%7CUnknown%7CTWFpbGZsb3d8eyJFbXB0eU1hcGkiOnRydWUsIlYiOiIwLjAuMDAwMCIsIlAiOiJXaW4zMiIsIkFOIjoiTWFpbCIsIldUIjoyfQ%3D%3D%7C0%7C%7C%7C&sdata=%2BBnZBZ%2B67jEc0xKEJAnRb6sDG2sS1%2B0xi%2BXCZfdvIbE%3D&reserved=0

We appreciate the reviewer’s thoughtful concern and the reference to Howard et al., which provides important evidence of the risks associated with site-specific biases in histopathology datasets. In designing our dataset split, we were mindful of these challenges and adopted a strategy aimed at balancing generalizability with practical constraints around data diversity and ethical considerations.

The full dataset comprises whole slide images (WSIs) from eight distinct centres, each using potentially different staining protocols and preparation workflows. To construct the test set, we deliberately excluded all WSIs from two of these centres (Lab 6 and Lab 8), ensuring that approximately 75% of the test set originates from entirely unseen staining protocols. This portion of the test set allows us to meaningfully assess out-of-distribution (OOD) generalization, which we agree is essential for evaluating the robustness of machine learning models.

In addition to this, the remaining 25% of the test set consists of a 10% random sample of slides drawn from the six centres that are also represented in the training set. The inclusion of these in-distribution samples was intentional: it enables us to evaluate how well the model performs on data that comes from distributions similar to the training data. Moreover, restricting the test set to only two centres would have risked introducing another form of bias — namely, the potential for the model to perform well simply because it aligns more closely with the specific characteristics of those two sites. By including a smaller proportion of data from additional centres in the test set, we increase its diversity and reduce the risk of overfitting to idiosyncrasies of a limited number of protocols.

Finally, we ensured that the dataset was balanced across diagnostic categories, subcategories, and staining protocols across all splits — training, validation, and testing — to further reduce the likelihood of class imbalance or site-specific confounding effects. It is also important to emphasize that the primary purpose of this GigaScience Data Note is to publicly release a high-quality, well-annotated dataset for the research community. Our goal is to facilitate future work by computer scientists and biomedical engineers, enabling them to develop and benchmark novel algorithms for histopathological image analysis. As such, we have prioritized transparency, accessibility, and broad utility, while acknowledging and documenting any limitations inherent to the dataset design.
