# Supplementary material for: Cervical whole-slide images dataset for multiclass classification
Source: Gigascience. 2025 Nov 29;14:giaf144. doi: 10.1093/gigascience/giaf144 (PMC12751090; doi:10.1093/gigascience/giaf144)
Supplement: giaf144_Authors_Response_To_Reviewer_Comments_Original_Submission [file giaf144_authors_response_to_reviewer_comments_original_submission.pdf]

Author's Response To Reviewer Comments

Reviewer #2:
1. This work is pretty well-structured. Please consider summarizing repetitive information to enhance readability. The abstract is informative but can be more succinct. Focus on the core contributions and potential impacts in fewer words.

We have amended the abstract to be more succinct as requested.

2. The annotation process is crucial for the dataset's quality. However, the paper lacks detailed information on the inter-observer variability and consensus-building among pathologists. Including metrics on annotation agreement and methods to resolve discrepancies would strengthen the dataset's reliability.

We have added a section to explain how we dealt with interobserver variation, based on our original research paper (Mohammadi et al, "Automated reporting of cervical biopsies using artificial intelligence", https://doi.org/10.1371/journal.pdig.0000381) which presented that Cohen's kappa (p.11-12, 3.Inter observer variations) was utilised to calculate the score between three observers in discrepant cases and the higher level of agreement was selected.

3. The details on the selection criteria for pathologists, such years of experience and if they are the specialists, in the annotation process and any training they received to ensure consistency.

The manuscript summarises relevant details about the annotation team and their qualifications. All histopathologists who annotated biopsies are registered with GMC and Royal College of Pathologists, with subspecialty expertise in gynaecological pathology. Each participating pathologist has a minimum of 5-10 years of post-specialist training experience, with some having over 15 years of expertise in gynaecological pathology. To ensure high-quality and standardised annotations, pathologists reviewed a detailed annotation protocol based on internationally recognised guidelines (WHO classification of tumours, 5th Edition).

4. The paper mentions various staining protocols and tissue handling methods and such variations are crucial to build a robust model. Discussion on how these variations can be quantified and/or normalized or accounted for during data preprocessing.

The data from different laboratories were combined before selecting the training set, on the basis as we state, that mixing these slides from different sources minimises the chance of over fitting related to technical differences which are not controllable.

5. The paper could benefit from a detailed evaluation of the dataset's utility. Including results from preliminary experiments using the dataset to demonstrate its effectiveness in training machine learning models.

Preliminary results are present in the primary research paper (Mohammadi et al, "Automated reporting of cervical biopsies using artificial intelligence", https://doi.org/10.1371/journal.pdig.0000381) in which we reported an AI model to assist in the diagnosis of cervical biopsies. The model was trained on a dataset of whole slide images (WSIs) of cervical biopsies, which were annotated by expert pathologists. The AI systems was designed to identify and classify various cervical lesions, aiming to reduce the workload of pathologists by highlighting areas of interest and potentially decreasing the time required for diagnosis. The study demonstrated that the AI model could effectively distinguish between different types of cervical tissue abnormalities, suggesting its potential utility in clinical settings to support pathologists in the diagnostic process. We believe that reference to this paper should help the user of the dataset to formulate their own plans and experiments.

6. Authors can compare this dataset with existing cervical image datasets. Highlight unique aspects and advantages, such as size, diversity, or annotation quality.

This is the only large study with images of cervical biopsies as opposed to cytological preparations: there is no comparable dataset available as far as we know. We have brought this point out.

7. The authors can include information on the licensing and ethical considerations for using the dataset in research and clinical applications. If this dataset can be used for commercialization purpose? If there is any legal liability of using the given dataset to build the diagnostic models.

As we described in the section “Data Availability”, this dataset can be freely accessed for any purpose without requirement of permission from the authors. It is ethically approved for release and is completely anonymised. As regards the question of legal liability, this is a generic issue, but it is most unlikely that a commercial developer could or would rely on a single dataset upon which to base an application for regulatory approval.

8. Authors can outline and discussion potential future directions for expanding the dataset or improving its annotations.

We are unclear what additional discussion would be valuable. For any clinically useful tool to be developed and implemented, further equivalent datasets would need to be made available.

9. The paper does not address the potential for inter-observer variability in annotations and quality control. This is a critical aspect that can affect the dataset's reliability and should be discussed in detail, while this does not impair the current value of the dataset.

Please see the answers of Q2 and Q3. We believe we have addressed these issues and that pathologists advising any future projects would be very aware of the subjective nature of pathological classification.

10. The technical processes, such as the conversion of image formats and the specifics of machine learning algorithms used, are not sufficiently detailed. Readers would benefit from a deeper understanding of these processes.

The process is described in Mohammadi et al, “Automated reporting of cervical biopsies using artificial intelligence”, https://doi.org/10.1371/journal.pdig.0000381, including how to convert image formats and the specifics of machine learning algorithms. Our experience in discussion with investigators requesting access to data is that they wish to develop their own methodologies depending on the purpose for which they require the data.

11. Understand this is mainly a database paper, but the lack of preliminary results demonstrating the dataset's utility in machine learning applications is a significant omission. Including such results would provide concrete evidence of the dataset's value. If the author has a research paper published, in preparation or archived, can indicate in the manuscripts, which will not affect the sharing this database through a paper in the community.

We already have included Reference 5, which describes the original research which we believe provides a useful framework for other investigators to use this dataset. In our preliminary discussions and sharing drafts with Dr Chris Armit and colleagues, we understood that providing the largest dataset of cervical biopsies without any restrictions as to use would be seen as sufficient value.

12. A comprehensive guideline for researchers on how to use the dataset effectively is very much helpful to increase the impact of this database, including best practices for preprocessing and annotation usage.

In discussions with colleagues, it is clear there is no uniform approach to using data. We believe this is out of scope for a data note and would require consensus across pathology as a discipline. We hope that by providing completely free access to the data that a community will use the information thus inform and refine guidelines and best use principles. That is our primary reason for making these data available.

13. Please ensure that the dataset and any accompanying code are accessible through public repositories.

Provide clear instructions for accessing and using the dataset.

This has been done and referenced, following guidance from Dr Chris Armit when he was working with GigaScience.

Reviewer #3:
14. The slides are provided in the iSyntax format, which is not compatible with the OpenSlide library - the most commonly used in the ML community. Offering the slides in a more universal format, such as TIFF, could greatly enhance the accessibility and usability of the dataset for a broader audience.

Glencoe software was used to convert iSyntax format to ome-tiff.
https://www.glencoesoftware.com/blog/2019/12/09/converting-whole-slide-images-to-OME-TIFF.html as described in the text.
In preliminary discussions with Dr Armit, we agreed that although iSYNTAX can be an irritation, it is possible to convert images, and some investigators want access to Philips-originated images precisely to resolve its relative difficulty of accessibility.

15. The proposed dataset-split deliberately places WSIs from the same lab (or at least the same preparation protocol) in both the training and testing sets. I would be interested in understanding the rationale behind this choice, as it could potentially lead to inflated test accuracy.

Please see the answer of reviewer2 -Q4. We believe the mixing actually reduces the chance of bias and overfitting. In addition, a key principle of the ethical approval granted for completely open access, freely available data was its complete de-identifying of images, linking it to individual pathologists or sites. The alternative was a much smaller database being made available or to restrict access. As there is no equivalent set of cases, we felt it was more important to make as large a cohort as possible freely available.

16. The writing in the manuscript could be improved for clarity. At times, the current phrasing makes it challenging to understand the authors' intentions. Below, I've outlined some paragraphs where clarity could be improved:

"Context" Section:
The main point of this paragraph is somewhat unclear. It presents mostly WSI analysis algorithms (presenting in detail two of those works - maybe you could detail why those works are important in this context?). However, because this paper is presenting a new dataset, I would find very useful to present here the other available public datasets of cervical biopsies as well as their sizes and characteristics, for instance.

Another dataset - “Robust whole slide image analysis for cervical cancer screening using deep learning”, Nature communications, 2021 Sep 24; 12(1):1–0, https://doi.org/10.1038/s41467-021-25296-x was described in Mohammadi et al, “Automated reporting of cervical biopsies using artificial intelligence”, https://doi.org/10.1371/journal.pdig.0000381.
17. "Data Split to Train and Test Sets" Section:
The sentence, "During the annotation process, these labels were double-checked, and in approximately 5% of the cases, the final label associated with the scanned slide was different," could be clarified. In particular, it is unclear what the initial labels.

We have clarified the text

18. The phrase, "The corrected labels post-annotation were the labels used for training and testing," would benefit from further clarification. Does this imply that the ML algorithm (« training and testing.. ») was trained before the slides were fully annotated?
The reference to "[…] described above" is somewhat vague. To which percentages are you referring to ?

We have clarified this section

19. "Annotation Process" Section:
I understand from this section that each slide is assigned both a main label and a sub-label. If pixel-level annotation masks are also available, it would be useful to provide additional details on this process also. The statement, "Primary annotation was performed either by one of the four pathologists or by a biomedical scientist specifically trained for this project," seems to conflict with the earlier mention that each slide is randomly assigned to one of the four pathologists. Additionally, it would be helpful to clarify whether the "primary annotation" is the same as the "main label" mentioned later. If so, further information on who performed the sub-category annotations would be valuable.

We have attempted to clarify this. First annotations were undertaken by expert biomedical scientists. Then they were allocated randomly to the pathologists to review the annotations. If there was no consensus a third review was undertaken. Histopathology is by its very essence subjective. By having multiple reviews and consensus we have tried to minimise variation.

20. Including the identity of the annotator (encoded as a discrete value) in the metadata could be beneficial, as it might help account for this potential source of bias.

Discussion of interobserver variation has been improved. As discussed above, we were encouraged by the ethics committee to release minimum information that might lead to identifiability. We made a judgement call that having a freely available large dataset outweighed more personalised information about reviewers, even though it would have been anonymous. We have attempted to reduce bias by always ensuring there is a consensus diagnosis from at least two observers, rather than relying on a single observer.

21. "Data Validation and Quality Control" Section:
This paragraph could be clearer, particularly the sentence: "The gold standard was the pathologists' diagnosis and where there was discrepancies, by consensus review. » I did not understand it.

We have altered wording.

22. The relevance of the subsequent paragraphs, "Gynaecological Cancer AI Project" and "Extracting Nuclear Morphological Features Using Indica Halo AI," is not clear to me. In the former, the algorithm is described with insufficient detail for reproduction or comprehension ("The features extracted from the heatmaps generated at the patch level are used for training an ML classifier to form the final slide-level predictions for each slide." What final ML classifier is used? Which features are extracted from the heatmaps?). In the latter, while the idea of extracting nuclear morphology per pathology class is promising, it's unclear why it is described here. Do you provide this extraction? What are the performance metrics for the ML models trained on these features?

The specific questions are addressed in reference 5. We have attempted to clarify the text.

23. Other Remarks:
There are some minor errors in the text (e.g., "the achieves of the Glasgow Royal Infirmary," or nested parentheses errors like "(low grade (including …()") that could benefit from careful proofreading.
Some figures, such as "architecture.png," are of very low quality and could be improved to better support the manuscript.

Thank you for drawing our attention to these points

Editorial Manager®

Cookie Preference Center

We use cookies which are necessary to make our site work. We may also use additional cookies to analyze, improve and personalize our content and your digital experience. For more information, see our Cookie Policy.

You may choose not to allow some types of cookies. However, blocking some types may impact your experience of our site and the services we are able to offer. See the different category headings below to find out more or change your settings.

Allow all

Manage Consent Preferences

Strictly Necessary Cookies

Always active

These cookies are necessary for the website to function and cannot be switched off in our systems. They are usually only set in response to actions made by you which amount to a request for services, such as setting your privacy preferences, logging in or filling in forms. You can set your browser to block or alert you about these cookies, but some parts of the site will not then work.

Functional Cookies

These cookies enable the website to provide enhanced functionality and personalisation. They may be set by us or by third party providers whose services we have added to our pages. If you do not allow these cookies then some or all of these services may not function properly.

Performance Cookies

These cookies allow us to count visits and traffic sources so we can measure and improve the performance of our site. They help us to know which pages are the most and least popular and see how visitors move around the site.

Targeting Cookies

These cookies may be set through our site by our advertising partners. They may be used by those companies to build a profile of your interests and show you relevant adverts on other sites. If you do not allow these cookies, you will experience less targeted advertising.

Cookie List

Clear

Apply Cancel

Consent Leg.Interest

Confirm my choices

Powered by onetrust
