# Supplementary material for: Cervical whole-slide images dataset for multiclass classification
Source: Gigascience. 2025 Nov 29;14:giaf144. doi: 10.1093/gigascience/giaf144 (PMC12751090; doi:10.1093/gigascience/giaf144)
Supplement: giaf144_Authors_Response_To_Reviewer_Comments_Revision_1 [file giaf144_authors_response_to_reviewer_comments_revision_1.pdf]

Author's Response To Reviewer Comments

Dear Editor,

We would like to express our sincere thanks for the opportunity to revise and resubmit our manuscript entitled: “Cervical Wh
We have carefully considered all comments and have made corresponding revisions to the manuscript. Please find the attach
We hope that our revisions and clarifications we have provided sufficiently address all concerns and further enhance the quali
Thank you again for your time and consideration. We look forward to your feedback.

Yours faithfully,
In Hwa Um

Reviewer #3:

14. Slide format:
While I appreciate the discussion on this topic, my stance remains unchanged: a significant portion of the pathology and mac
To address this, could the authors consider providing instructions or a Docker environment with a script to facilitate format co
This issue is, in my opinion, the primary obstacle to the dataset's widespread adoption.

We appreciate the reviewer’s continued attention to this important issue. We fully agree that the ISYNTAX file format poses li
enables the conversion of ISYNTAX files to the widely adopted OME-TIFF format. This resource is hosted on Zenodo (Referenc
To further enhance accessibility and reproducibility, we have also provided a Docker environment within the same Zenodo rep
following DOI: https://doi.org/10.5281/zenodo.7674764
We confirm that while OME-TIFF is not directly supported by OpenSlide, it is compatible with widely used open tools such as

15. Why do you believe so? I would appreciate a clear justification for the authors' belief in this regard. Increasing the numbe
lack means to know that. Please see : Howard, Frederick M., James Dolezal, Sara Kochanny, Jefree Schulte, Heather Chen, L
https://eur01.safelinks.protection.outlook.com/?url=https%3A%2F%2Fdoi.org%2F10.1038%2Fs41467-021-24698-1&data=
andrews.ac.uk%7C4e289b4df86f43b8505508dd5da84e77%7Cf85626cb0da849d3aa5864ef678ef01a%7C0%7C0%7C638769
XCZfdvIbE%3D&reserved=0

We appreciate the reviewer’s thoughtful concern and the reference to Howard et al., which provides important evidence of th
diversity and ethical considerations.
The full dataset comprises whole slide images (WSIs) from eight distinct centres, each using potentially different staining pro
This portion of the test set allows us to meaningfully assess out-of-distribution (OOD) generalization, which we agree is esse
In addition to this, the remaining 25% of the test set consists of a 10% random sample of slides drawn from the six centres t
restricting the test set to only two centres would have risked introducing another form of bias — namely, the potential for the
overfitting to idiosyncrasies of a limited number of protocols.
Finally, we ensured that the dataset was balanced across diagnostic categories, subcategories, and staining protocols across
release a high-quality, well-annotated dataset for the research community. Our goal is to facilitate future work by computer s
documenting any limitations inherent to the dataset design.

Cookie Preference Center

We use cookies which are necessary to make our site work. We may also use additional cookies to analyze, improve and personalize our content and your digital experience. For more information, see our Cookie Policy.

You may choose not to allow some types of cookies. However, blocking some types may impact your experience of our site and the services we are able to offer. See the different category headings below to find out more or change your settings.

Allow all

Manage Consent Preferences

Strictly Necessary Cookies

Always active

These cookies are necessary for the website to function and cannot be switched off in our systems. They are usually only set in response to actions made by you which amount to a request for services, such as setting your privacy preferences, logging in or filling in forms. You can set your browser to block or alert you about these cookies, but some parts of the site will not then work.

Functional Cookies

These cookies enable the website to provide enhanced functionality and personalisation. They may be set by us or by third party providers whose services we have added to our pages. If you do not allow these cookies then some or all of these services may not function properly.

Performance Cookies

These cookies allow us to count visits and traffic sources so we can measure and improve the performance of our site. They help us to know which pages are the most and least popular and see how visitors move around the site.

Targeting Cookies

These cookies may be set through our site by our advertising partners. They may be used by those companies to build a profile of your interests and show you relevant adverts on other sites. If you do not allow these cookies, you will experience less targeted advertising.

Cookie List

Clear

Apply Cancel
Consent Leg.Interest

Confirm my choices

Powered by onetrust
