# Supplementary material for: Cervical whole-slide images dataset for multiclass classification
Source: Gigascience. 2025 Nov 29;14:giaf144. doi: 10.1093/gigascience/giaf144 (PMC12751090; doi:10.1093/gigascience/giaf144)
Supplement: giaf144_Reviewer_2_Report_Original_Submission — Weimiao Yu -- 8/3/2024 [file giaf144_reviewer_2_report_original_submission.pdf]

Reviewer Report

Title: Cervical Whole Slide Images Dataset for Multi-class Classification

Version: Original Submission **Date:** 8/3/2024

Reviewer name: Weimiao Yu

Reviewer Comments to Author:

Review Comments for "Cervical Whole Slide Images Dataset for Multi-class Classification"The availability of high-quality database is always a bottleneck in this AI Digital Pathology field. The authors presented a newly constructed large-scale dataset of cervical whole slide images (WSIs) intended to facilitate research into the detection, diagnosis, and treatment of cervical cancer. It contains 2539 WSI, each representing a unique patient, stored in iSyntax format. The images are manually annotated by pathologists and categorized into ~10 diagnostic classes and subcategories. The data is stratified into training and test sets to maintain balanced proportions across categories. The dataset is valuable for training machine learning algorithms to aid in automated detection of cervical abnormalities, benefiting medical professionals in making more accurate and timely diagnoses. The dataset can be used for training and evaluating machine learning models, educational purposes, and advancing research in cervical cancer diagnosis.1. This work is pretty well-structured. Please consider summarizing repetitive information to enhance readability.The abstract is informative but can be more succinct. Focus on the core contributions and potential impacts in fewer words.2. The annotation process is crucial for the dataset's quality. However, the paper lacks detailed information on the inter-observer variability and consensus-building among pathologists. Including metrics on annotation agreement and methods to resolve discrepancies would strengthen the dataset's reliability.3. The details on the selection criteria for pathologists, such years of experience and if they are the specialists, in the annotation process and any training they received to ensure consistency.4. The paper mentions various staining protocols and tissue handling methods and such variations are crucial to build a robust model. Discussion on how these variations can be quantified and/or normalized or accounted for during data preprocessing.5. The paper could benefit from a detailed evaluation of the dataset's utility. Including results from preliminary experiments using the dataset to demonstrate its effectiveness in training machine learning models.6. Authors can compare this dataset with existing cervical image datasets. Highlight unique aspects and advantages, such as size, diversity, or annotation quality.7. The authors can include information on the licensing and ethical considerations for using the dataset in research and clinical applications. If this dataset can be used for commercialization purpose? If there is any legal liability of using the given dataset to build the diagnostic models.8. Authors can outline and discussion potential future directions for expanding the dataset or improving its annotations.Some other comments:* The paper does not address the potential for inter-observer variability in annotations and quality control. This is a critical aspect that can affect the dataset's reliability and should be discussed in detail, while this does not impair the current value of the dataset.* The technical processes, such as the conversion of image formats and the specifics of machine learning algorithms used, are not sufficiently detailed. Readers would benefit from a deeper understanding of these processes.* Understand this is mainly a database

paper, but the lack of preliminary results demonstrating the dataset's utility in machine learning applications is a significant omission. Including such results would provide concrete evidence of the dataset's value. If the author has a research paper published, in preparation or archived, can indicate in the manuscripts, which will not affect the sharing this database through a paper in the community.* A comprehensive guideline for researchers on how to use the dataset effectively is very much helpful to increase the impact of this database, including best practices for preprocessing and annotation usage.* Please ensure that the dataset and any accompanying code are accessible through public repositories. Provide clear instructions for accessing and using the dataset.

Level of Interest

Please indicate how interesting you found the manuscript: Choose an item.

Quality of Written English

Please indicate the quality of language in the manuscript: Choose an item.

Declaration of Competing Interests

Please complete a declaration of competing interests, considering the following questions:

- Have you in the past five years received reimbursements, fees, funding, or salary from an organisation that may in any way gain or lose financially from the publication of this manuscript, either now or in the future?
- Do you hold any stocks or shares in an organisation that may in any way gain or lose financially from the publication of this manuscript, either now or in the future?
- Do you hold or are you currently applying for any patents relating to the content of the manuscript?
- Have you received reimbursements, fees, funding, or salary from an organization that holds or has applied for patents relating to the content of the manuscript?
- Do you have any other financial competing interests?
- Do you have any non-financial competing interests in relation to this paper?

If you can answer no to all of the above, write 'I declare that I have no competing interests' below. If your reply is yes to any, please give details below.

N.A

I agree to the open peer review policy of the journal. I understand that my name will be included on my report to the authors and, if the manuscript is accepted for publication, my named report including any attachments I upload will be posted on the website along with the authors' responses. I agree for my report to be made available under an Open Access Creative Commons CC-BY license

(http://creativecommons.org/licenses/by/4.0/). I understand that any comments which I do not wish to be included in my named report can be included as confidential comments to the editors, which will not be published.

Choose an item.

To further support our reviewers, we have joined with Publons, where you can gain additional credit to further highlight your hard work (see: https://publons.com/journal/530/gigascience). On publication of this paper, your review will be automatically added to Publons, you can then choose whether or not to claim your Publons credit. I understand this statement.

Yes Choose an item.
