# Supplementary material for: Cervical whole-slide images dataset for multiclass classification
Source: Gigascience. 2025 Nov 29;14:giaf144. doi: 10.1093/gigascience/giaf144 (PMC12751090; doi:10.1093/gigascience/giaf144)
Supplement: giaf144_Reviewer_3_Report_Original_Submission — Tristan Lazard -- 8/14/2024 [file giaf144_reviewer_3_report_original_submission.pdf]

Reviewer Report

Title: Cervical Whole Slide Images Dataset for Multi-class Classification

Version: Original Submission **Date:** 8/14/2024

Reviewer name: Tristan Lazard

Reviewer Comments to Author:

Mohammadi et al. propose the release of a large dataset of stained cervical biopsy WSIs, which was previously utilized in the iCAIRD project. Because of its size, diversity, and accompanying annotations, this dataset has a great potential and could be valuable to the community.Strengths:The dataset is large.The metadata reportedly includes detailed information related to slide preparation. In my opinion, this participates in the high value of the dataset. This rather rare information could be used to study in detail the impact of different slide preparation processes on image processing algorithms, this source of bias being one of the major barrier to the clinical adoption of ML algorithms.Each slide is accompanied by a detailed diagnosis annotation, as well as a discrete mask detailing the precise location of the lesions.Weaknesses:The slides are provided in the iSyntax format, which is not compatible with the OpenSlide library - the most commonly used in the ML community. Offering the slides in a more universal format, such as TIFF, could greatly enhance the accessibility and usability of the dataset for a broader audience.The proposed dataset-split deliberately places WSIs from the same lab (or at least the same preparation protocole) in both the training and testing sets. I would be interested in understanding the rationale behind this choice, as it could potentially lead to inflated test accuracy.The writing in the manuscript could be improved for clarity. At times, the current phrasing makes it challenging to understand the authors' intentions. Below, I've outlined some paragraphs where clarity could be improved:"Context" Section:The main point of this paragraph is somewhat unclear. It presents mostly WSI analysis algorithms (presenting in detail two of those works - maybe you could detail why those works are important in this context ?). However, because this paper is presenting a new dataset, I would find very useful to present here the other available public datasets of cervical biopsies as well as their sizes and characteristics, for instance."Data Split to Train and Test Sets" Section:The sentence, "During the annotation process, these labels were double-checked, and in approximately 5% of the cases, the final label associated with the scanned slide was different," could be clarified. In particular, it is unclear what the initial labels.The phrase, "The corrected labels post-annotation were the labels used for training and testing," would benefit from further clarification. Does this imply that the ML algorithm (Â« training and testing.. Â») was trained before the slides were fully annotated?The reference to "[…] described above" is somewhat vague. To which percentages are you referring to ?"Annotation Process" Section:I understand from this section that each slide is assigned both a main label and a sub-label. If pixel-level annotation masks are also available, it would be useful to provide additional details on this process also.The statement, "Primary annotation was performed either by one of the four pathologists or by a biomedical scientist specifically trained for this project," seems to conflict with the earlier mention that each slide is randomly assigned to one of the four pathologists. Additionally, it would be helpful to clarify whether the "primary annotation" is the same as the "main label" mentioned later. If

so, further information on who performed the sub-category annotations would be valuable.Including the identity of the annotator (encoded as a discrete value) in the metadata could be beneficial, as it might help account for this potential source of bias."Data Validation and Quality Control" Section:This paragraph could be clearer, particularly the sentence: "The gold standard was the pathologists' diagnosis and where there was discrepancies, by consensus review. Â» I did not understand it.The relevance of the subsequent paragraphs, "Gynaecological Cancer AI Project" and "Extracting Nuclear Morphological Features Using Indica Halo AI," is not clear to me. In the former, the algorithm is described with insufficient detail for reproduction or comprehension ("The features extracted from the heatmaps generated at the patch level are used for training an ML classifier to form the final slide-level predictions for each slide." What final ML classifier is used? Which features are extracted from the heatmaps?). In the latter, while the idea of extracting nuclear morphology per pathology class is promising, it's unclear why it is described here. Do you provide this extraction? What are the performance metrics for the ML models trained on these features?Other Remarks:There are some minor errors in the text (e.g., "the achieves of the Glasgow Royal Infirmary," or nested parentheses errors like "(low grade (including …()") that could benefit from careful proofreading.Some figures, such as "architecture.png," are of very low quality and could be improved to better support the manuscript.Conclusion:Overall, this dataset holds significant potential and could benefit the research community. However, I believe that the manuscript would benefit from a thorough revision to enhance clarity and address the issues mentioned above.

Level of Interest

Please indicate how interesting you found the manuscript: Choose an item.

Quality of Written English

Please indicate the quality of language in the manuscript: Choose an item.

Declaration of Competing Interests

Please complete a declaration of competing interests, considering the following questions:

- Have you in the past five years received reimbursements, fees, funding, or salary from an organisation that may in any way gain or lose financially from the publication of this manuscript, either now or in the future?
- Do you hold any stocks or shares in an organisation that may in any way gain or lose financially from the publication of this manuscript, either now or in the future?
- Do you hold or are you currently applying for any patents relating to the content of the manuscript?
- Have you received reimbursements, fees, funding, or salary from an organization that holds or has applied for patents relating to the content of the manuscript?
- Do you have any other financial competing interests?
- Do you have any non-financial competing interests in relation to this paper?

If you can answer no to all of the above, write 'I declare that I have no competing interests' below. If your reply is yes to any, please give details below.

I declare that I have no competing interests

I agree to the open peer review policy of the journal. I understand that my name will be included on my report to the authors and, if the manuscript is accepted for publication, my named report including any attachments I upload will be posted on the website along with the authors' responses. I agree for my report to be made available under an Open Access Creative Commons CC-BY license (http://creativecommons.org/licenses/by/4.0/). I understand that any comments which I do not wish to be included in my named report can be included as confidential comments to the editors, which will not be published.

Choose an item.

To further support our reviewers, we have joined with Publons, where you can gain additional credit to further highlight your hard work (see: https://publons.com/journal/530/gigascience). On publication of this paper, your review will be automatically added to Publons, you can then choose whether or not to claim your Publons credit. I understand this statement.

Yes Choose an item.
