# Supplementary material for: Cervical whole-slide images dataset for multiclass classification
Source: Gigascience. 2025 Nov 29;14:giaf144. doi: 10.1093/gigascience/giaf144 (PMC12751090; doi:10.1093/gigascience/giaf144)
Supplement: giaf144_Reviewer_3_Report_Revision_1 — Tristan Lazard -- 2/27/2025 [file giaf144_reviewer_3_report_revision_1.pdf]

Reviewer Report

Title: Cervical Whole Slide Images Dataset for Multi-class Classification

Version: Revision 1 **Date:** 2/27/2025

Reviewer name: Tristan Lazard

Reviewer Comments to Author:

14. Slides format:While I appreciate the discussion on this topic, my stance remains unchanged: a significant portion of the pathology and machine learning community relies on OpenSlide-compatible formats.This discrepancy may limit the dataset's impact (I have never used OME-TIFF but from what I can read, it is compatible with QuPath but not OpenSlide. Do you confirm?)To address this, could the authors consider providing instructions or a Docker environment with a script to facilitate format conversion?This issue is, in my opinion, the primary obstacle to the dataset's widespread adoption.15. Why do you believe so ? I would appreciate a clear justification for the authors' belief in this regard. Increasing the number of centers in the training set may have this effect - but if some centers effectively correlate with the output variable, then having this same center in the test set will artificially inflate test results - and with the current dataset design, we lack means to know that. Please see : Howard, Frederick M., James Dolezal, Sara Kochanny, Jefree Schulte, Heather Chen, Lara Heij, Dezheng Huo, et al. Â« The Impact of Site-Specific Digital Histology Signatures on Deep Learning Model Accuracy and Bias Â». Nature Communications 12, nÂµ’ 1 (dÃ©cembre 2021): 4423. https://doi.org/10.1038/s41467-021-24698-1.I understand the privacy concerns involved. Could the authors provide a measure of the mutual information between the variable "center" (where the slide was prepared) and the output variable? A low mutual information value would be reassuring, as it would indicate minimal correlation between the center and the labels.This point is important: if test values can be optimized by enhancing the model's ability to recognize the center, and if this dataset becomes a standard benchmark in the field, it could negatively impact the development of new algorithms.Thank you for adding the paragraph regarding inter-observer variability.Overall however I believe this dataset would be a useful addition to the public WSIs datasets, and a nice contribution to open research

Level of Interest

Please indicate how interesting you found the manuscript: Choose an item.

Quality of Written English

Please indicate the quality of language in the manuscript: Choose an item.

Declaration of Competing Interests

Please complete a declaration of competing interests, considering the following questions:

- Have you in the past five years received reimbursements, fees, funding, or salary from an organisation that may in any way gain or lose financially from the publication of this manuscript, either now or in the future?
- Do you hold any stocks or shares in an organisation that may in any way gain or lose financially from the publication of this manuscript, either now or in the future?
- Do you hold or are you currently applying for any patents relating to the content of the manuscript?
- Have you received reimbursements, fees, funding, or salary from an organization that holds or has applied for patents relating to the content of the manuscript?
- Do you have any other financial competing interests?
- Do you have any non-financial competing interests in relation to this paper?

If you can answer no to all of the above, write 'I declare that I have no competing interests' below. If your reply is yes to any, please give details below.

I declare that I have no competing interests

I agree to the open peer review policy of the journal. I understand that my name will be included on my report to the authors and, if the manuscript is accepted for publication, my named report including any attachments I upload will be posted on the website along with the authors' responses. I agree for my report to be made available under an Open Access Creative Commons CC-BY license (http://creativecommons.org/licenses/by/4.0/). I understand that any comments which I do not wish to be included in my named report can be included as confidential comments to the editors, which will not be published.

Choose an item.

To further support our reviewers, we have joined with Publons, where you can gain additional credit to further highlight your hard work (see: https://publons.com/journal/530/gigascience). On publication of this paper, your review will be automatically added to Publons, you can then choose whether or not to claim your Publons credit. I understand this statement.

Yes Choose an item.
